# Stereoselective synthesis of an advanced *trans*-decalin intermediate towards the total synthesis of anthracimycin†

**DOI:** 10.1039/d4cc01738b

**Published:** 2024-05-07

**Authors:** Laksamee Jeanmard, Giacomo Lodovici, Ian George, Joshua T. W. Bray, Adrian C. Whitwood, Gavin H. Thomas, Ian J. S. Fairlamb, William P. Unsworth, Paul A. Clarke

**Affiliations:** a Department of Chemistry, University of York Heslington York YO10 5DD UK ian.fairlamb@york.ac.uk william.unsworth@york.ac.uk; b Department of Biology, University of York Heslington York YO10 5DD UK

## Abstract

Progress towards the total synthesis of the macrolide natural product anthracimycin is described. This new approach utilises an intermolecular Diels–Alder strategy followed by epimeirsation to form the key *trans*-decalin framework. The route culminates in the stereoselective synthesis of an advanced tricyclic lactone intermediate, containing five contiguous sterogenic centres with the correct relative and absolute stereochemistry required for the anthracimycin core motif.

## Abstract 2

A stereoselective synthesis of an advanced *trans*-decalin intermediate towards the total synthesis of anthracimycin is described.

## Graphical abstract

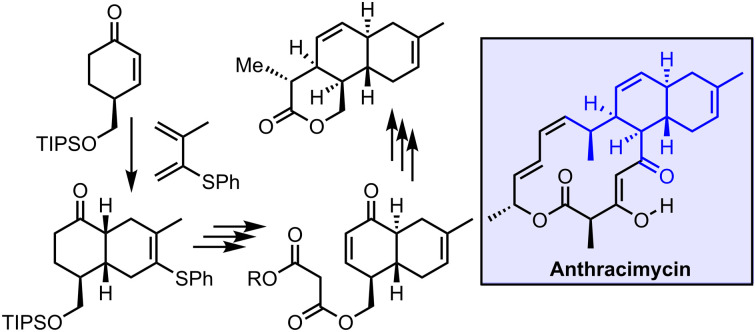

Anthracimycin 1 is a 14-membered ring macrolide antibiotic isolated from a marine microorganism, first reported by Fenical and co-workers in 2013.^1–3^ It was found to have potent antibacterial activity against several bacterial pathogens, including various MRSA strains, the anthrax-causing bioterrorism agent *B. anthracis* and tuberculosis.^1,4^ In view of the urgent need to find new antibiotics to tackle ever-growing resistance,^5^ it is therefore an important target for total synthesis.

The first total synthesis of anthracimycin 1 was completed by Brimble and co-workers in 2020^6*a*,*b*^ who reported an elegant 20-step synthesis, with macrocyclisation *via* ring-closing metathesis using a Grubbs-type catalyst in the final step. This was followed by a remarkable convergent approach developed by Qian, Tong and co-workers. Macrolactonisation was first attempted to form the macrocycle, but again it was ring-closing metathesis that was successful. Both methods make use of intramolecular Diels–Alder reactions as a key step, to assemble the *trans*-decalin motif, a strategy that was informed by biosynthetic studies.^7^

In this communication we describe work towards the enantioselective total synthesis of anthracimycin 1, culminating in the synthesis of an advanced, stereochemically-rich tricyclic lactone intermediate 2. A key step in our retrosynthesis of 2 is an intramolecular Michael addition, starting from enone 3 to form a δ-lactone. Enone 3 was envisioned to be assembled from *cis*-decalin 4, including an epimerisation step to access the required *trans*-decalin stereochemistry. The synthesis is completed with an intermolecular Diels–Alder reaction between enone 5 and diene 6 was planned. Notably, this strategy is complementary to the intramolecular Diels–Alder approach used in the previous total syntheses, reported independently by Brimble^6*a*,*b*^ and Qian/Tong (Scheme 1).^6*c*^

**Scheme 1:**
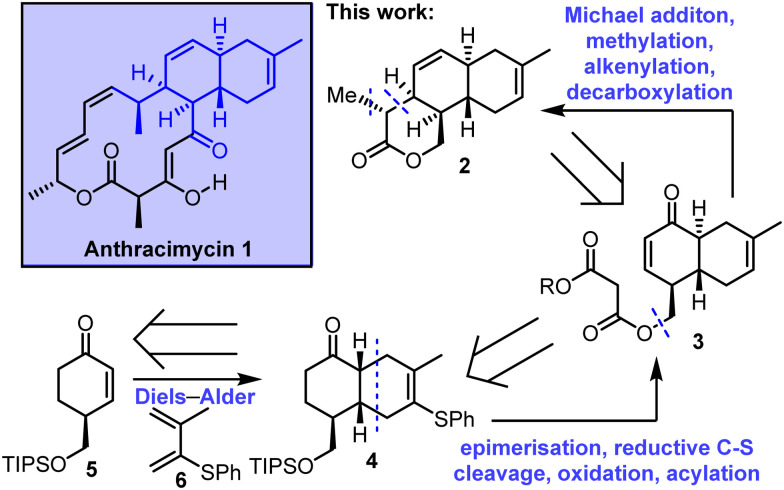
Stereoselective synthesis of an advanced tricyclic lactone intermediate 2 towards the total synthesis of anthracimycin.

Different methods to prepare the key enone starting material 5 were developed, both in racemic and in enantioenriched forms (Scheme 2). First, ketoester 7 was converted into TIPS-protected diol 8*via* LiAlH_4_ reduction and silylation of the more reactive primary alcohol. Subsequent Swern oxidation afforded ketone 9 in high yield, over the 3-step sequence. Classical Ito–Saegusa oxidation^8^ of ketone 9 was first attempted but was discounted following reproducibility problems associated with silyl enol ether stability (not shown). Direct oxidation conditions were instead used, inspired by the work of Stahl and coworkers (Scheme 2A).^9^ Various additives were explored for this transformation,^10^ with details of the conditions tested included in the ESI† (see Tables S1–S3). The inclusion of bipyridine ligands was found to improve conversion markedly, with its combination with Pd(OAc)_2_ {formally Pd_3_(OAc)_6_} under an oxygen atmosphere at 120 °C enabling enone 5 to be formed in 70% yield (Scheme 2A, conditions A). The inclusion KNO_3_ as an additive enabled a further improvement (85% yield, Scheme 2A, conditions B). We believe that positive Pd–NO_*x*_ (*x* = 1–3) interactions could assist the oxidative process, akin to other Pd^II^-catalysed reactions.^11^ Mechanistic studies into these effects are ongoing within our laboratories.

**Scheme 2:**
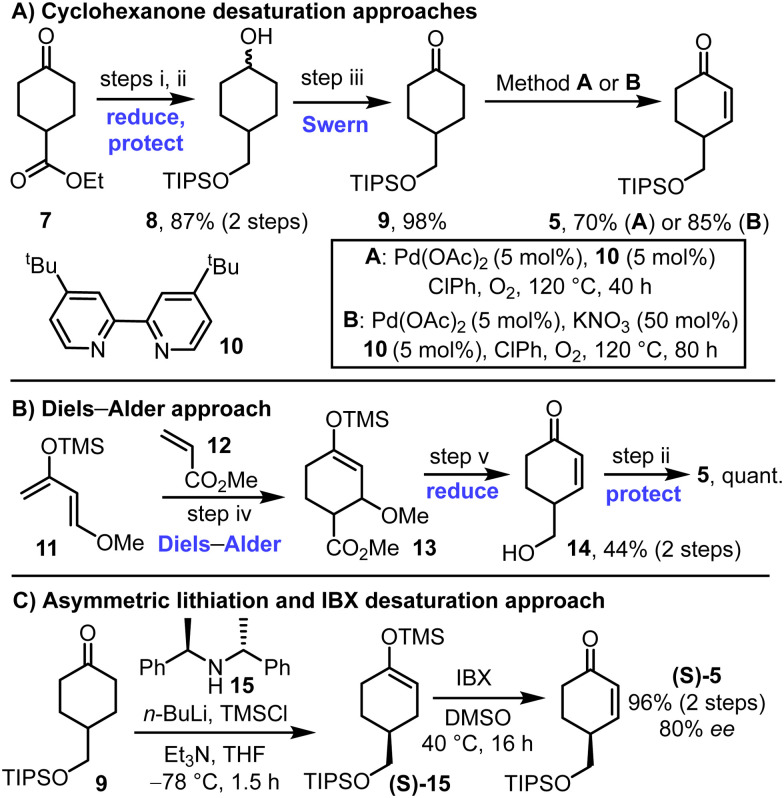
Approaches for the synthesis of enone 5. (i) LiAlH_4_, THF, 0 °C to RT, 18 h; (ii) TIPS-Cl, imidazole, DMAP, CH_2_Cl_2_, RT, 16 h; (iii) oxalyl chloride, DMSO, CH_2_Cl_2_, −78 °C, 1 h, then NEt_3_, RT, 1.5 h; (iv) diene 11, alkene 12, toluene, 80 °C, 45 h, (v) LiAlH_4_, Et_2_O, −78 °C to RT over 4 h.

An alternative approach to racemic enone 5 is summarised in Scheme 2B; this method makes use of a Diels–Alder reaction between diene 11 and dienophile 12, followed by LiAlH_4_ reduction and TIPs protection to form the same enone 5.^12^

To prepare enone 5 in enantioenriched form, an asymmetric lithiation strategy was used to form silyl enol ether (*S*)-15, followed by IBX oxidation. This approach, which was inspired by a method developed by Smith and coworkers, enabled the formation enone (*S*)-5 in 96% yield and 80% ee.^13,14^ The absolute stereochemistry of the major enantiomer is assumed to be the *S*-isomer shown by analogy to the enantioselectivity observed in Smith's method for closely related compounds.^13^ Note that while racemic enone 5 was useful during the route development phase of this study, the remaining synthetic schemes in this manuscript, and characterisation data described in the ESI,† all relate to chemistry performed on precursors derived from the enantioenriched enone (*S*)-5.

Enantioenriched enone (*S*)-5 was reacted with diene 6 in the key intermolecular Diels–Alder reaction. The reaction was promoted by catalytic EtAlCl_2_, and afforded *cis*-decalin 4 as a single diastereoisomer.^15^ Approach of the diene on the opposite face to the bulky OTIPS group likely accounts for the observed diastereoselectivity, with the stereochemical assignment of 4 supported by nOe data (see Fig. S1–S3, ESI†). Prolonged treatment of *cis*-decalin 4 with catalytic EtAlCl_2_ (3 days) then enabled smooth epimerisation to give the required *trans*-decalin scaffold, with *trans*-decalin 16 isolated in 81% yield. Cleavage of the C–S bond was then performed *via* hydrogenolysis with RANEY®-Nickel, to afford alkene 17 in 86% yield; importantly, this reaction proceeded well without competing alkene reduction. At this stage, the relative stereochemistry of 17 was confirmed using X-ray crystallography *via* hydrazone 19,^16^ which was formed by a condensation reaction between ketone 17 and hydrazine 18 (Scheme 3 and Fig. S4, ESI†).

**Scheme 3:**
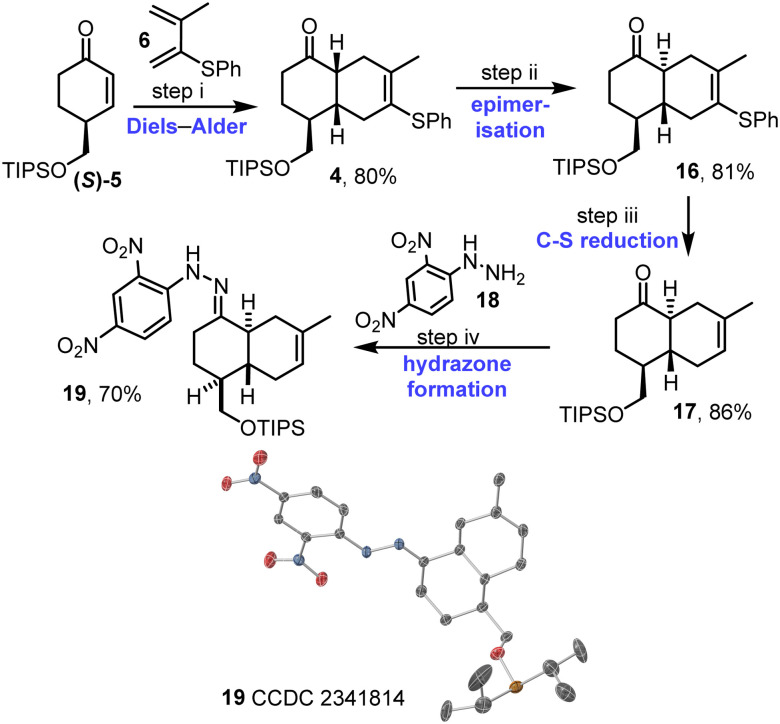
Synthesis of *trans*-decalin 17. (i) (*S*)-5, diene 6, EtAlCl_2_, CH_2_Cl_2_, RT, 1 h; (ii) EtAlCl_2_, CH_2_Cl_2_, RT, 3 d; (iii) RANEY®-Ni, H_2_, acetone, RT, 1 h; (iv) 18, AcOH, 3 Å mol sieves, MeOH, 50 °C, 16 h. Single crystal X-ray diffraction structure visualised in CrystalMaker v11.0.02: thermal ellipsoids set at 50% probability level, H-atoms omitted for clarity; oxygen atoms in red, carbon in grey, nitrogen in blue and silicon in light orange. The disorder in the OTIPS group is not shown.

The synthesis continued with the desaturation of ketone 17; this was done *via* the formation of silyl enol ether 20 and IBX oxidation to afford enone 21, which proceeded in good overall yield (Scheme 4). Following cleavage of the TIPS protecting group, acylation of the resulting alcohol 22 with ethyl malonyl chloride then afforded malonate derivative 23. The key intramolecular Michael addition step was first attempted under various basic conditions,^17^ but these attempts were affected by problems associated with unwanted elimination of the malonate group (see Table S4, ESI†). Instead, a procedure combining La(O-iPr)_3_ and Hünig's base was tested, inspired by a report from Shibasaki and coworkers.^18^ This approach was successful, with the desired tricycle 24 obtained in 75% yield as a single diastereoisomer. The assigned stereochemistry of 24 matches that required for anthracimycin and is supported both by nOe studies (see Fig. S5–S7, ESI†) and X-ray crystallographic data^16^ obtained for tricycle 24 (Scheme 4 and Fig. S8, ESI†).

**Scheme 4:**
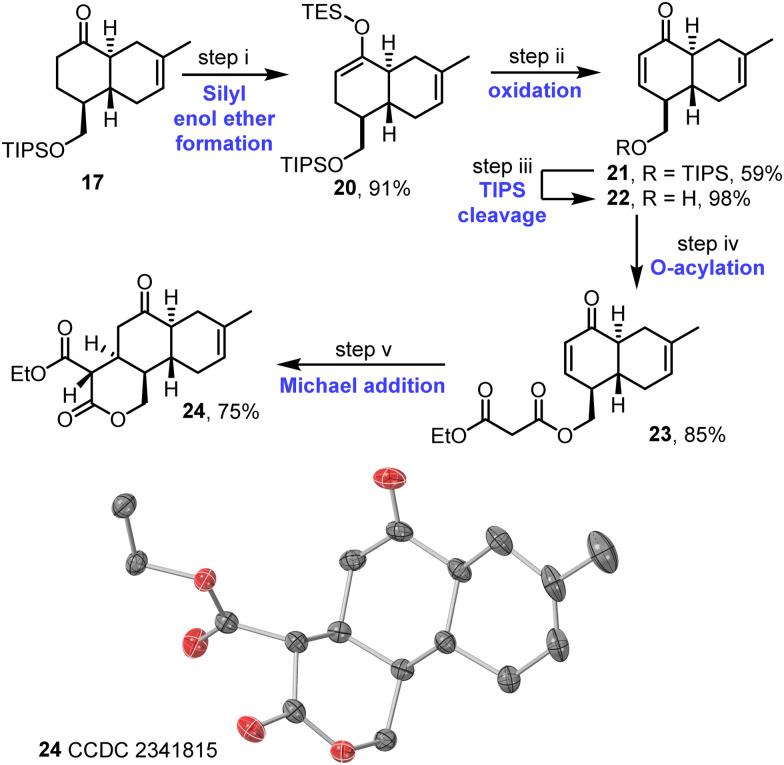
Synthesis of tricycle 24. (i) LDA, ketone 17, THF, then TESCl, −78 °C to RT, 1 h; (ii) IBX, DMSO, 60 °C, 3 d; (iii) TBAF, THF, RT, 2 h; (iv) ethyl malonyl chloride, NEt_3_, CH_2_Cl_2_, 0 °C to RT, 2 h; (v) La(O-i-Pr)_3_, THF, i-Pr_2_NEt, 40 °C, 7 h. Single crystal X-ray diffraction structure visualised in CrystalMaker v11.0.02: thermal ellipsoids set at 50% probability level, H-atoms omitted for clarity; oxygen atoms in red and carbon in grey.

Methylation of tricycle 24 proceeded smoothly using sodium hydride and methyl iodide in THF (Scheme 5). This furnished methylated product 25 in 71% yield, as a single diastereoisomer, with the assigned relative stereochemistry supported by nOe studies (see Fig. S9–S12, ESI†). Next, ketone 25 was converted into vinyl triflate 26 using Comins’ reagent.^19^ Then, reduction of triflate 26 in the presence of the Pd^II^ pre-catalyst system Pd(OAc)_2_/PPh_3_,^20^ formic acid, and tributylamine afforded alkene 27 in 75% yield. Finally, concomitant ester hydrolysis and decarboxylation was carried out using LiOH and H_2_O_2_ in THF/water at 60 °C. This afforded diastereomeric tricyclic lactones 2 and 28 in 40% and 20% yields respectively, with the diastereomeric products epimeric at the methyl-substituted position. Pleasingly, lactone 2, the major of the two diastereomers formed, was found to have the required relative stereochemistry for anthracimycin; this assignment is supported by nOe studies performed on both isomers 2 and 28 (see Fig. S13–S21, ESI†).

**Scheme 5:**
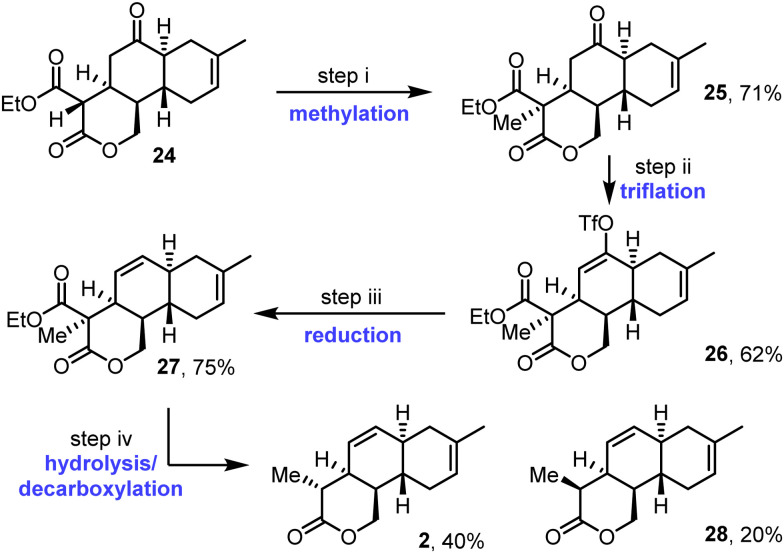
Synthesis of tricycles 2 and 28. (i) NaH, MeI, THF, RT, 6 h; (ii) LDA, *N*-(5-chloro-2-pyridyl)triflimide, THF, −78 °C to RT; (iii) *N*Bu_3_, Pd(OAc)_2_(PPh_3_)_2_, formic acid, DMF, 50 °C, 2.5 h; (iv) LiOH, H_2_O_2_, THF/H_2_O, 60 °C, 6.5 h.

In conclusion, significant progress has been made towards the total synthesis of anthracimycin, through the stereoselective synthesis of an advanced tricyclic lactone intermediate 2. Lactone 2 contains five contiguous stereogenic centres, with the correct relative and absolute configurations required for the natural product. A key feature of the new retrosynthetic strategy is the use of an intermolecular Diels–Alder approach to form the decalin framework, which contrasts with the biomimetic intramolecular Diels–Alder strategy used in Brimble's^6*a*,*b*^ and Qian/Tong's synthetic routes.^6*c*^

With 5 of the 7 stereogenic centres of anthracimycin installed, and with synthetic handles in place primed for further elaboration, the completion of the total synthesis from 2 is a realistic proposition.¶¶Unfortunately, work to complete the total synthesis of anthracimycin will not continue in York as Prof Paul A. Clarke passed away in November 2023. This manuscript is therefore presented for the scientific record, and to disclose the viability of this new approach. Other researchers interested in completing the total synthesis of anthracimycin based on the results described herein, are strongly encouraged. For example, a sequence of reduction, Wittig olefination, oxidation and thioester formation could be used to convert lactone 2 into thioester 29,^21^ an advanced intermediate in Brimble's total synthesis (Scheme 6).^6*a*,*b*^

**Scheme 6:**
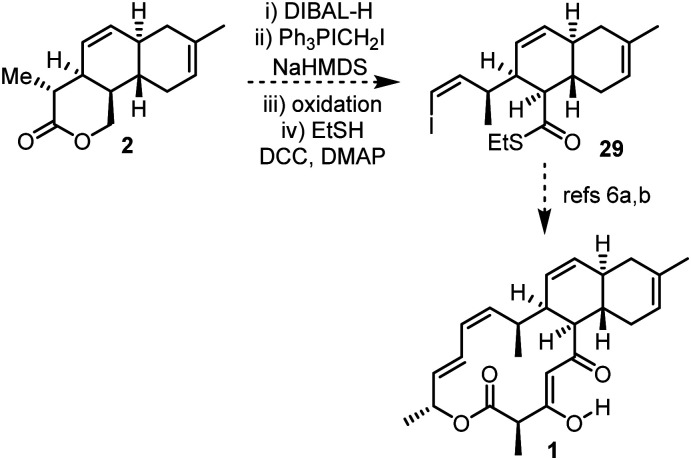
Proposed route to complete the total synthesis of anthracimycin 1*via* Brimble's intermediate 29.

Synthetic studies were done by L. J., G. L. and I. G. The project on anthracimycin was conceived, designed and led exclusively by P. A. C. Collaborative work between P. A. C. and I. J. S. F. on Pd–NO_*x*_ interactions (Ito–Saegusa chemistry) was on-going at the time of him passing away. J. T. W. B. was involved in assisting experiments exploring the Pd–NO_*x*_ interactions involved in the Ito–Saegusa oxidation chemistry. The paper was written by W. P. U. and I. J. S. F., with contributions from L. J., G. L., I. G and J. T. W. B.

We are very grateful for financial support from The Development and Promotion of Science and Technology Talents Project (DPST), Royal Thai Government (L. J.), the University of York (G. L.), the Biotechnology and Biological Sciences Research Council [EP/M008401/1, I. G.]. I.J.S.F. thanks the Royal Society for an Industry Fellowship (2021–25) and the EPSRC IAA scheme for funding. We are grateful to Niels Koning and Connor Prior for performing related anthracimycin studies within the Clarke group that contributed to some of the ideas and direction of the work described in this manuscript.

## Conflicts of interest

There are no conflicts to declare.

## Supplementary Material

CC-060-D4CC01738B-s001

CC-060-D4CC01738B-s002
