# Supplementary material for: Stereoselective synthesis of an advanced trans-decalin intermediate towards the total synthesis of anthracimycin
Source: Chem Commun (Camb). 2024 May 7;60(44):5699–702. doi: 10.1039/d4cc01738b (PMC11131352; doi:10.1039/d4cc01738b)
Supplement: CC-060-D4CC01738B-s001 [file CC-060-D4CC01738B-s001.pdf]

Electronic Supplementary Material (ESI) for ChemComm.
This journal is © The Royal Society of Chemistry 2024

Table of Contents

1) General Information	2
2) Synthetic procedures and characterisation data	3–35
3) Additional optimisation tables	36–38
4) ^{1}H and ^{13}C NMR spectra	39–57
5) References	57

1) General Information

Except where stated, all reagents were purchased from commercial sources and used without further purification. Anhydrous CH_2Cl_2 and THF were obtained from an Innovative Technology Inc. PureSolv® solvent purification system. ^1H NMR and ^{13}C NMR spectra were recorded on a JEOL ECX400 or JEOL ECS400 spectrometer (operating at 400 MHz and 100 MHz). All Spectroscopic data was acquired at 295 K unless stated otherwise. Chemical shifts (δ) are quoted in parts per million (ppm). The residual solvent peaks, δ_H 7.26 and δ_C 77.16 for $CDCl_3$ were used as a reference. Coupling constants (*J*) are reported in Hertz (Hz) to the nearest 0.1 Hz. The multiplicity abbreviations used are: br s broad singlet, s singlet, d doublet, br d broad doublet, t triplet, br t broad triplet, q quartet, p pentet, dd doublet of doublets, ddd doublet of doublet of doublets, dddd doublet of doublet of doublet of doublets, dt doublet of triplets, ddt doublet of doublet of triplets, td triplet of doublets, m multiplet. Signal assignment was achieved by analysis of DEPT, COSY, HMBC and HSQC experiments where required. In cases where products were formed as a mixture of rotamers, their ratio was determined by integration of signals in the ^1H NMR spectrum. Infrared (IR) spectra were recorded on a PerkinElmer UATR 2 spectrometer as a thin film dispersed from either CH_2Cl_2 or $CDCl_3$. Mass spectra (high-resolution) were obtained by the University of York Mass Spectrometry Service, using Electrospray Ionisation (ESI) on a Bruker Daltonics, Micro-tof spectrometer. Melting points were determined using Gallenkamp apparatus. Thin layer chromatography was carried out on Merck silica gel $60F_{254}$ pre-coated aluminium foil sheets and were visualised using UV light (254 nm) and stained with basic aqueous potassium permanganate or ceric ammonium nitrate. Flash column chromatography was carried out using slurry packed Fluka silica gel (SiO_2), 35–70 µm, 60 Å, under a light positive pressure, eluting with the specified solvent system.

2) Synthetic procedures and characterisation data

4-(((Triisopropylsilyl)oxy)methyl)cyclohexan-1-ol (8)

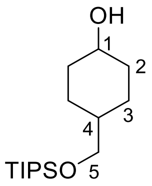

To a suspension of $LiAlH_4$ (3.60 g, 94.8 mmol, 2.0 equiv) in THF (150 mL) at 0 °C was slowly added ethyl 4-oxocyclohexanecarboxylate **7** (7.50 mL, 47.4 mmol). The reaction mixture was stirred under N_2 atmosphere at 0 °C and allowed to reach room temperature for 18 hours. The reaction mixture was then cooled back to 0 °C and quenched with H_2O (3.6 mL), 15% NaOH (3.6 mL), followed by H_2O (10.8 mL). The mixture was stirred for a further 15 minutes and allowed to reach room temperature. Anhydrous $MgSO_4$ was added, and the mixture was filtered through Celite, which was washed with Et_2O. The combined organic layers were concentrated *in vacuo* to give a diol as a white sticky residue (6.17 g) which was directly used for the next step without further purification. To a solution of the crude diol (6.17 g, assumed to be 47.4 mmol) in CH_2Cl_2 (190 mL) was added imidazole (4.84 g, 71.1 mmol, 1.5 equiv), DMAP (590 mg, 4.74 mmol, 0.1 equiv), followed by triisopropylsilyl chloride (11.2 mL, 52.1 mmol, 1.1 equiv). The reaction mixture was stirred under N_2 atmosphere at room temperature for 16 hours. After this time, an aqueous solution of 2 M HCl (75 mL) was added, and the organic layer was separated. The aqueous layer was extracted with CH_2Cl_2 (3 × 50 mL). The combined organic layers were washed with H_2O (50 mL) and brine (50 mL), dried over anhydrous Na_2SO_4 and concentrated *in vacuo*. The crude product was purified by silica gel flash column chromatography (10−30% EtOAc/hexane) to yield the title compound **8** as a 2:1 mixture of diastereoisomers, as colourless oil (11.8 g, 87% over two steps). **Minor isomer 8**: $\boldsymbol{R_f}$ = 0.36 (20% EtOAc/hexane); **IR** (ATR) ν_{max} 3350 (O-H), 2925, 2865, 1463, 1197, 1064, 882, 783, 680 cm^{-1}; **^{1}H NMR** (400 MHz, $CDCl_3$) δ 4.01−3.98 (1H, m, H-1), 3.50 (2H, dd, *J* = 18.2, 6.1 Hz, H-5), 1.75−1.69 (2H, m, H-2 + H-3), 1.60−1.51 (5H, m, H-2 + H-3 + H-4) 1.45−1.37 (3H, m, H-2 + H-3 and OH), 1.11−0.97 (21H, m, $OSi(CH(CH_3)_2)_3$) ppm; **^{13}C NMR** (100 MHz, $CDCl_3$) δ 68.1 (C-1), 67.2 (C-5), 39.5 (C-4), 32.2 (C-2), 23.6 (C-3), 18.2 ($OSi(CH(\underline{C}H_3)_2)_3$), 12.1 ($OSi(\underline{C}H(CH_3)_2)_3$) ppm; **HRMS** (ESI) *m/z* cald for $C_{16}H_{34}NaO_2Si$ ($M+Na^+$) 309.2220, found 309.2211. **Major isomer 8**: $\boldsymbol{R_f}$ = 0.25 (20% EtOAc/hexane); **IR** (ATR) ν_{max} 3339 (O-H), 2928, 2865, 1463, 1115, 1067, 882, 802, 681 cm^{-1}; **^{1}H NMR** (400 MHz, $CDCl_3$) δ 3.55 (1H, dd, *J* = 10.8, 10.8, 4.2, 4.2 Hz, H-1), 3.46

(2H, dd, *J* = 18.9, 6.2 Hz, H-5), 2.02−1.96 (2H, m, H-2), 1.85−1.79 (2H, m, H-2) 1.51−1.37 (3H, m, H-3 + H-4 and OH), 1.30−1.19 (3H, m, H-3), 1.12−0.95 (21H, m, $OSi(CH(CH_3)_2)_3$) ppm; **^{13}C NMR** (100 MHz, $CDCl_3$) δ 71.4 (C-1), 68.5 (C-5), 39.9 (C-4), 35.3 (C-2), 27.9 (C-3), 18.2 ($OSi(CH(\underline{C}H_3)_2)_3$), 12.1 ($OSi(\underline{C}H(CH_3)_2)_3$) ppm; **HRMS** (ESI) *m/z* cald for $C_{16}H_{34}NaO_2Si$ ($M+Na^+$) 309.2220, found 309.2214.

4-(((Triisopropylsilyl)oxy)methyl)cyclohexan-1-one (9)

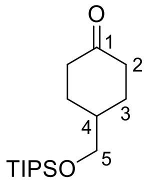

To a solution of oxalyl chloride (2.73 mL, 32.2 mmol, 1.20 equiv) in CH_2Cl_2 (250 mL) at −78 °C was added DMSO (2.38 mL, 33.6 mmol, 1.25 equiv) dropwise. The mixture was stirred under N_2 atmosphere at −78 °C for 1 hour before being added a solution of alcohol **8** (7.69 g, 26.8 mmol) in CH_2Cl_2 (30 mL). The reaction mixture was stirred at −78 °C for 1 hour before being added Et_3N (18.7 mL, 134 mmol, 5.0 equiv). The mixture was allowed to reach room temperature for 1.5 hours. After this time, the mixture was quenched with H_2O (16 mL) and 2 M HCl (78 mL). The organic layer was separated, and the aqueous layer was extracted with CH_2Cl_2 (3 x 60 mL). The combined organic layers were washed with 2M HCl (50 mL) and brine (50 mL), dried over anhydrous Na_2SO_4 and concentrated *in vacuo*. The crude residue was purified by silica gel flash column chromatography (5−10% EtOAc/hexane) to afford the title compound **9** as a colourless oil (7.49 g, 98% yield): $\boldsymbol{R_f}$ = 0.27 (10% EtOAc/hexane); **IR** (ATR) ν_{max} 2943, 2866, 1717 (C=O), 1463, 1121, 1104, 1067, 882, 682 cm^{-1}; **^{1}H NMR** (400 MHz, $CDCl_3$) δ 3.60 (2H, d, *J* = 6.2 Hz, H-5), 2.41 (2H, ddd, *J* = 14.3, 4.8, 3.1 Hz, H-2), 2.34 (2H, ddd, *J* = 14.3, 12.6, 6.2 Hz, H-2), 2.11 (2H, dddd, *J* = 13.2, 6.2, 6.2, 3.1 Hz, H-3), 1.95 (1H, tttd, *J* = 12.6, 6.2, 6.2, 3.6 Hz, H-4), 1.46 (2H, dddd, *J* = 13.2, 12.6, 12.6, 4.8 Hz, H-3), 1.14−1.03 (21H, m, $OSi(CH(CH_3)_2)_3$) ppm; **^{13}C NMR** (100 MHz, $CDCl_3$) δ 212.6 (C-1), 67.4 (C-5), 40.7 (C-2), 39.2 (C-4), 29.4 (C-3), 18.2 ($OSi(CH(\underline{C}H_3)_2)_3$), 12.1 ($OSi(\underline{C}H(CH_3)_2)_3$) ppm; **HRMS** (ESI) *m/z* cald for $C_{16}H_{32}NaO_2Si$ ($M+Na^+$) 307.2064, found 307.2063. Elemental analysis: Found: C, 66.96, H, 11.34, N, 0. $C_{16}H_{32}O_2Si$ requires C, 67.55, H, 11.34, N, 0.

6-(((Triisopropylsilyl)oxy)methyl)cyclohex-13-enone (5)

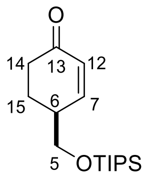

Using Method A in Scheme 2A

To a solution of cyclohexanone **9** (1.67 g, 5.88 mmol) in chlorobenzene (60.0 mL) were added $Pd(OAc)_2$ (66.0 mg, 0.290 mmol) and 4,4′-di-tert-butyl-2,2′-dipyridyl (79.0 mg, 0.290 mmol) and the reaction was stirred at 120 °C under a O_2 balloon. After 72 hours, additional $Pd(OAc)_2$ (66.0 mg, 0.290 mmol) and 4,4′-di-tert-butyl-2,2′-dipyridyl (79.0 mg, 0.290 mmol) were added and the O_2 balloon refreshed. After a further 44 hours, additional $Pd(OAc)_2$ (66.0 mg, 0.290 mmol) and 4,4′-di-tert-butyl-2,2′-dipyridyl (79.0 mg, 0.290 mmol) were added and the O_2 balloon refreshed. The reaction was stirred for a further 45 hours, then concentrated *in vacuo* to give a brown oil (2.50 g). The crude product was purified by silica gel flash column chromatography (3%–10% EtOAc in hexane) and by Kugelrohr distillation to yield the title compound **5** as a pale yellow oil (1.17 g, 70% yield).

Using Method B in Scheme 2A

To a solution of cyclohexanone **9** (10.6 g, 37.5 mmol) in chlorobenzene (375 mL), were added $Pd(OAc)_2$ (420 mg, 1.88 mmol), 4,4′-di-tert-butyl-2,2′-dipyridyl (510 mg, 1.88 mmol) and KNO_3 (1.90 g, 18.8 mmol) and the reaction was stirred at 120 °C under a balloon of O_2. After 48 hours, additional $Pd(OAc)_2$ (420 mg, 1.88 mmol), 4,4′-di-tert-butyl-2,2′-dipyridyl (510 mg, 1.88 mmol) were added and the O_2 balloon refreshed. The O_2 balloon was refreshed every 24 hours and after a further 5 days, the reaction was cooled to room temperature and concentrated *in vacuo*. The crude product was purified by silica gel flash column chromatography (3%–10% EtOAc in hexane) and by Kugelrohr distillation to yield the title compound **5** as a pale yellow oil (9.0 g, 85% yield).

From silyl enol ether rac-15

To a solution of crude TMS-enol ether (0.75 g, 2.1 mmol, 1.0 equiv) in DMSO (30 mL) was added IBX (2.94 g, 10.5 mmol, 5.0 equiv). The reaction mixture was stirred at 40 °C under N_2 atmosphere overnight before being cooled to room temperature. The reaction mixture was quenched with a saturated aqueous solution of $NaHCO_3$ and diluted with hexane (30 mL). The organic layer was separated, and the aqueous layer was extracted with hexane (3 x 30 mL).

The combined organic layers were washed with brine, dried over anhydrous Na_2SO_4 and concentrated *in vacuo*. The crude residue was purified by silica gel flash column chromatography (5−10% EtOAc/hexane) to the title compound **5** as a light yellow oil (515 mg, 87% yield over two steps)

Data for **5**: B.P. 147 °C @ 0.6 mbar; $\boldsymbol{R_f}$ = 0.38 (10% EtOAc/hexane) **IR** (ATR): ν_{max} 2942, 2891, 2865, 1682 (C=O), 1462, 1389, 1110, 881, 783, 681 cm^{-1}.**^{1}H NMR** (400 MHz, $CDCl_3$): δ 7.00 (1H, ddd, *J* = 10.2, 2.7, 1.4 Hz, H-7), 6.05 (1H, dd, *J* = 10.2, 2.5 Hz, H-12), 3.76 (1H, dd, *J* = 9.6, 6.4 Hz, H-5), 3.68 (1H, dd, *J* = 9.6, 6.9 Hz, H-5), 2.63 (1H, dddddd, *J* = 9.6, 6.9, 6.4, 4.6, 2.7, 2.5 Hz, H-6), 2.54 (1H, ddd, *J* = 16.5, 4.6, 4.6 Hz, H-14), 2.39 (1H, ddd, *J* = 16.5, 12.8, 5.0 Hz, H-14), 2.10 (1H, ddddd, *J* = 13.3, 5.0, 4.6, 4.6, 1.4 Hz, H-15), 1.79 (1H, dddd, *J* = 13.3, 12.8, 9.6, 4.6 Hz, H-15), 1.15–1.00 (21H, m, $OSiCH(CH_3)_2$) ppm. **^{13}C NMR** (400 MHz, $CDCl_3$): δ 199.9 (C-13), 152.1 (C-7), 129.8 (C-12), 65.8 (C-5), 29.4 (C-14), 36.7 (C-6), 25.4 (C-15), 17.9 ($OSiCH(\underline{C}H_3)_2$), 11.8 ($OSi\underline{C}H(CH_3)_2$) ppm. **MS** (ESI): m/z 305 ($M+Na^+$); HRMS: Found ($M+Na^+$), 305.1899 $C_{16}H_{30}NaO_2Si$ requires ($M+Na^+$) 305.1907. m/z 283 ($M+H^+$); Found ($M+H^+$), 283.2083. $C_{16}H_{31}O_2Si$ requires ($M+H^+$) 283.2088.

Methoxy-3-trimethylsilyloxy-1,3-butadiene (11)

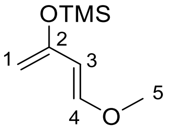

A solution of $ZnCl_2$ (0.17 g, 1.2 mmol, 3.0 mol%) in Et_3N (12.2 mL, 89.5 mmol, 2.2 equiv) was stirred at room temperature for 1 hour. To this solution was added a solution of *trans*-4-methoxy-3-butene-2-one (4.0 mL, 40.7 mmol) in benzene (20 mL), followed by TMSCl (10.3 mL, 81.4 mmol, 2.0 equiv) over a period of 30 minutes. The reaction mixture was stirred under N_2 atmosphere at 40 °C for 19 hours before being cooled to room temperature and diluted with Et_2O (150 mL). The mixture was filtered through Celite and washed with Et_2O. The filtrate was concentrated *in vacuo* and purified by kugelrohr distillation (40−42 °C at 2 mbar) to give the title compound **11** as a colourless oil (4.56 g, 65% yield): $\boldsymbol{R_f}$ = 0.60 (10% EtOAc/hexane); **^{1}H NMR** (400 MHz, $CDCl_3$) δ 6.83 (1H, d, *J* = 12.6 Hz, H-4), 5.35 (1H, d, *J* = 12.6 Hz, H-3), 4.11 (1H, s, H-1) 4.06 (1H, s, H-1) 3.58 (3H, s, H-5), 0.23 (9H, s, $OSi(\underline{CH_3})_3$) ppm. This compound was characterised only by ^{1}H NMR due to its volatility and instability. The data matched those reported in the literature.[1]

Methyl 2-methoxy-4-(trimethylsilyloxy)cyclohex-3-enecarboxylate (13)

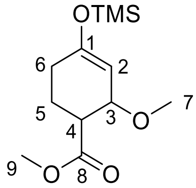

To a solution of diene **11** (0.43 g, 2.5 mmol) in toluene (1.7 mL) was added methyl acrylate (0.40 mL, 4.4 mmol, 1.7 equiv). The reaction mixture was stirred under N_2 atmosphere at 80 °C for 45 hours. The reaction mixture was then cooled to room temperature and concentrated *in vacuo* to yield a mixture of two diastereomers of cyclohexene **13** as a yellow oil, which was directly used for the next step without purification (569 mg, >99% conversion, 88% crude yield): ***R$_f$*** = 0.45 (30% EtOAc/hexane); **^{1}H NMR** (400 MHz, $CDCl_3$) δ 5.14 (1H, d, *J* = 5.7 Hz, H-2), 4.99−4.97 (1H, m, H-2), 4.27−4.23 (1H, m, H-3), 4.11 (1H, dd, *J* = 4.8, 4.8 Hz, H-3), 3.71 (3H, s, H-9), 3.70 (3H, s, H-9), 3.32 (3H, s, H-7), 3.29 (3H, s, H-7), 2.62−2.57 (1H, m, H-4), 2.54−2.49 (1H, m, H-4), 2.11−1.85 (8H, m, H-6 + H-5), 0.21 (9H, s, $OSi(CH_3)_3$) 0.20 (9H, s, $OSi(CH_3)_3$) ppm. This compound was characterised only by ^{1}H NMR due to its instability. The data matched those reported in the literature.[2]

4-(Hydroxymethyl)cyclohex-2-en-1-one (14)

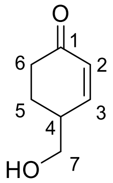

To a suspension of $LiAlH_4$ (54 mg, 1.4 mmol, 1.4 equiv) in Et_2O (5.5 mL) at −78 °C was added a solution of crude cyclohexene **13** (0.26 g, 1.0 mmol) in Et_2O (4.0 mL). The reaction mixture was stirred under N_2 atmosphere at −78 °C for 4 hours and then allowed to reach 0 °C. The reaction mixture was quenched with H_2O (0.05 mL), 15% NaOH (0.05 mL) followed by H_2O (0.15 mL) and allowed to reach room temperature. The mixture was added Na_2SO_4 and stirred at room temperature for 15 minutes before being filtered. The filtrate was concentrated *in vacuo*. The crude residue was purified by silica gel flash column chromatography (50% EtOAc/hexane) to the title compound **14** as a yellow oil (56 mg, 44% yield). ***R$_f$*** = 0.17 (60% EtOAc/hexane); **IR** (ATR) ν_{max} 3406 (O-H), 2927, 2871, 1662 (C=O), 1393, 1256, 1083, 1048, 845 cm^{-1}; **^{1}H NMR** (400 MHz, $CDCl_3$) δ 6.97 (1H, ddd, *J* = 10.2, 2.4, 1.2 Hz, H-3), 6.06 (1H, dd, *J* = 10.2, 2.6 Hz, H-2), 3.73 (1H, dd, *J* = 10.4, 6.4 Hz, H-7), 3.67 (1H, dd, *J* = 10.4, 6.6 Hz, H-7), 2.64 (1H, dddddd, *J* = 9.8, 6.6, 6.4, 4.8, 2.6, 2.4 Hz, H-4), 2.55 (1H, ddd, *J* = 16.8, 4.8, 4.8 Hz, H-6),

2.40 (1H, ddd, J = 16.8, 12.6, 4.8 Hz, H-6), 2.13 (1H, ddddd, J = 13.4, 4.8, 4.8, 4.8, 1.2 Hz, H-5), 1.81 (1H, dddd, J = 13.4, 12.6, 9.8, 4.8 Hz, H-5) ppm; **^{13}C NMR** (100 MHz, $CDCl_3$) δ 199.9 (C-1), 151.5 (C-3), 130.5 (C-2), 65.3 (C-7), 39.1 (C-4), 36.8 (C-6), 25.5 (C-5) ppm; **HRMS** (ESI) *m/z* cald for $C_7H_{10}NaO_2$ ($M+Na^+$) 149.0573, found 149.0572.

Triisopropyl((4-((trimethylsilyl)oxy)cyclohex-3-en-1-yl)methoxy)silane (*rac*-15)

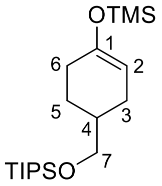

To a solution of freshly distilled diisopropylamine (0.36 mL, 2.5 mmol, 1.2 equiv) in THF (3 mL) at −78 °C, was added *n*-BuLi (1.4 mL of a 1.89 M solution in hexane, 2.5 mmol, 1.2 equiv) dropwise. After stirring for 45 minutes, to this solution was added a solution of cyclohexanone **9** (0.60 g, 2.1 mmol) in THF (9 mL) and stirred for a further 1 hour. Freshly distilled trimethylsilyl chloride (0.35 mL, 2.7 mmol, 1.3 equiv) was added at −78 °C. The reaction mixture was stirred at −78 °C for 15 minutes and allowed to reach room temperature. The reaction mixture was quenched with a saturated aqueous solution of $NaHCO_3$ (10 mL) and diluted with Et_2O (10 mL). The organic layer was separated, and the aqueous layer was extracted with Et_2O (3 x 20 mL). The combined organic layers were washed with brine, dried over anhydrous Na_2SO_4 and concentrated *in vacuo* to give racemic TMS-enol ether **rac-15** as a light yellow oil, which was directly used for the next step without purification (749 mg, >99% conversion).

(*S*)-Triisopropyl((4-((trimethylsilyl)oxy)cyclohex-3-en-1-yl)methoxy)silane ((*S*)-15)

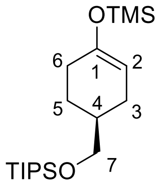

To a solution of (+)-bis[(*R*)-1-phenylethyl]amine (3.2 mL, 14.0 mmol, 1.3 equiv) in THF (43 mL) at −78 °C, was slowly added *n*-BuLi (6.85 mL of a 1.89 M solution in hexane, 12.9 mmol, 1.2 equiv). The pink solution was stirred under N_2 atmosphere at −78 °C for 30 minutes. Freshly distilled trimethylsilyl chloride (6.85 mL, 53.9 mmol, 5.0 equiv) was added dropwise at −78 °C where upon it turned colourless. After an additional 10 minutes, a solution of cyclohexanone **9** (3.06 g, 10.8 mmol) in THF (5 mL) was slowly added. The mixture was stirred for a further 1.5 hours before being treated with freshly distilled trimethylamine (13.7 mL, 108 mmol, 10.0

equiv) at −78 °C, followed by a saturated aqueous solution of $NaHCO_3$ (14 mL) below −20 °C. The mixture was allowed to reach room temperature. The organic layer was separated, and the aqueous layer was extracted with hexane (3 x 40 mL). The combined organic layers were washed with 3 N citric acid and 1 N citric acid (until all the amine byproduct was removed as determined by TLC and ninhydrin stain), and a saturated aqueous solution of $NaHCO_3$ and brine. The organic layer was dried over anhydrous Na_2SO_4 and concentrated *in vacuo* to give the crude product **(*S*)-15** as a pale oil, which was directly used for the next step without purification (3.85 g, >99% conversion).

(*S*)-4-(((Triisopropylsilyl)oxy)methyl)cyclohex-2-en-1-one ((*S*)-5)

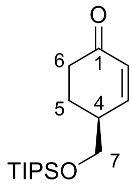

To a solution of crude TMS-enol ether **(*S*)-15** (3.85 g, 10.8 mmol, 1.0 equiv) in DMSO (150 mL) was added IBX (15.1 g, 54.0 mmol, 5.0 equiv). The reaction mixture was stirred at 40 °C under N_2 atmosphere overnight before being cooled to room temperature. The reaction mixture was quenched with a saturated aqueous solution of $NaHCO_3$ (100 mL) and diluted with hexane (100 mL). The organic layer was separated, and the aqueous layer was extracted with hexane (3 x 100 mL). The combined organic layers were washed with brine, dried over anhydrous Na_2SO_4 and concentrated *in vacuo*. The crude residue was purified by silica gel flash column chromatography (2% EtOAc/hexane) to afford (*S*)-cyclohexenone **(*S*)-5** as a pale oil (2.93 g, 96% yield over two steps, 80% *ee*): $\boldsymbol{R_f}$ = 0.19 (10% EtOAc/hexane); $\boldsymbol{[\alpha]_D^{20}}$ = −78.9 (*c* 1.00, $CHCl_3$); **IR** (ATR) ν_{max} 2942, 2891, 2865, 1682 (C=O), 1462, 1111, 1068, 881, 786, 681 cm^{-1}; ^{1}**H NMR** (400 MHz, $CDCl_3$) δ 7.00 (1H, ddd, *J* = 10.2, 2.6, 1.4 Hz, H-3), 6.05 (1H, dd, *J* = 10.2, 2.4 Hz, H-2), 3.75 (1H, dd, *J* = 9.6, 6.4 Hz, H-7), 3.67 (1H, dd, *J* = 9.6, 6.9 Hz, H-7), 2.62 (1H, dddddd, *J* = 9.9, 6.9, 6.4, 4.6, 2.6, 2.4 Hz, H-4), 2.54 (1H, ddd, *J* = 16.8, 4.6, 4.6 Hz, H-6), 2.39 (1H, ddd, *J* = 16.8, 12.8, 5.0 Hz, H-6), 2.09 (1H, ddddd, *J* = 13.2, 5.0, 4.6, 4.6, 1.4 Hz, H-5), 1.79 (1H, dddd, *J* = 13.2, 12.8, 9.9, 4.6 Hz, H-5), 1.11–1.01 (21H, m, $OSi(CH(CH_3)_2)_3$) ppm; ^{13}**C NMR** (100 MHz, $CDCl_3$) δ 200.1 (C-1), 152.3 (C-3), 130.1 (C-2), 66.0 (C-7), 39.6 (C-4), 36.9 (C-6), 25.6 (C-5), 18.1 ($OSi(CH(\underline{C}H_3)_2)_3$), 12.0 ($OSi(\underline{C}H(CH_3)_2)_3$) ppm; **HRMS** (ESI) *m/z* cald for $C_{16}H_{30}NaO_2Si$ ($M+Na^+$) 305.1907, found 305.1903. The enantiomeric excess (80% *ee*) was determined by HPLC

analysis using a CHIRALCEL® IC column eluting with 1% *i*-PrOH/hexane (flow rate = 1.0 mL/min, temp = 40 °C, λ = 216 nm): retention time = 15.0 min (major isomer), 16.0 min (minor isomer).

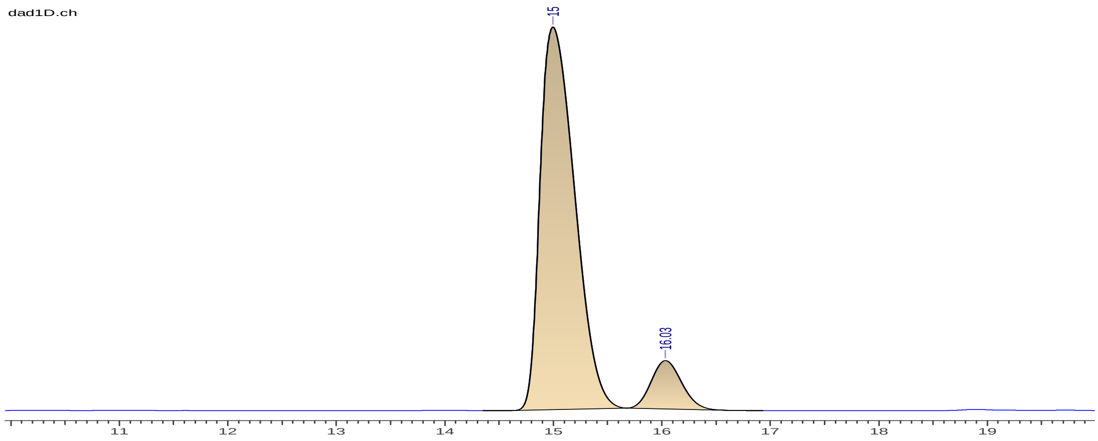

No.	tR	Peak Area (Y units*ms)	Area (%)	Width
1	15.00	27020104.000	89.977	0.600
2	16.03	3009888.250	10.023	0.518

3-Methyl-2-phenylthio-l,3-butadiene (6)

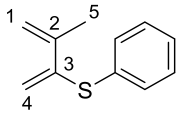

In a pressure vessel were placed $Pd(OAc)_2$ (69 mg, 0.31 mmol, 0.02 equiv), THF (7.6 mL), 2-methyl-1-buten-3-yne (1.5 mL, 15.7 mmol) and finally thiophenol (1.6 mL, 15.7 mmol, 1.0 equiv). The reaction mixture was stirred at 50 °C for 16 hours. After this period, the mixture was filtered through Celite and washed with EtOAc (50 mL). The filtrate was concentrated *in vacuo*. The crude product was purified by silica gel flash column chromatography (100% hexane) to yield diene **6** as a light yellow oil (2.35 g, 85% yield): $\boldsymbol{R_f}$ = 0.26 (100% hexane); **IR** (ATR) ν_{max} 3074, 3060, 2977, 1573, 1478, 1439, 1440, 1375, 1119, 1025 cm^{-1}; **^{1}H NMR** (400 MHz, $CDCl_3$) δ 7.31–7.16 (5H, m, Ar-H), 5.55 (1H, s, H-1), 5.52 (1H, s, H-4), 5.22 (1H, d, *J* = 1.2 Hz, H-1), 5.06 (1H, d, *J* = 1.2 Hz, H-4), 1.96 (3H, s, H-5) ppm; **^{13}C NMR** (100 MHz, $CDCl_3$) δ 144.2 (C-3), 140.9 (C-2), 134.8 (C-Ar), 131.4 (2CH-Ar), 129.2 (2CH-Ar), 127.1 (CH-Ar), 117.5 (C-1), 116.9 (C-4), 21.4 (C-5) ppm; **HRMS** (APCI) *m/z* cald for $C_{11}H_{13}S$ ($M+H^+$) 177.0732, found 177.0735. The characterisation data matched those reported previously.[3]

(4*S*,4a*R*,8a*R*)-7-Methyl-6-(phenylthio)-4-(((triisopropylsilyl)oxy)methyl)-3,4,4a,5,8,8a-hexahydronaphthalen-1(2*H*)-one (4)

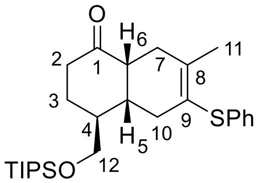

To a solution of enone **(*S*)-5** (0.38 g, 1.4 mmol, 1.0 equiv) in CH_2Cl_2 (3.5 mL) was added a 1.0 M solution of $EtAlCl_2$ in hexane (0.28 mL, 0.28 mmol, 0.2 equiv) and the mixture was stirred under a N_2 atmosphere at room temperature for 10 minutes. After this time, a solution of diene **6** (2.05 g, 11.6 mmol, 10.0 equiv) in CH_2Cl_2 (2.9 mL) was added to the mixture and the reaction mixture was stirred at room temperature for a further 1 hour. The reaction mixture was then quenched with Rochelle's salt 10% aqueous solution (10 mL) and stirred vigorously overnight. The organic layer was separated, and the aqueous layer was extracted with CH_2Cl_2) (3 x 10 mL). The combined organic layers were washed with brine, dried over anhydrous Na_2SO_4 and concentrated *in vacuo*. The crude residue was purified by silica gel flash column chromatography (5% Et_2O/hexane) to yield the title compound **4** as a yellow oil (488 mg, 80% yield): $\boldsymbol{R_f}$ = 0.07 (5% Et_2O/hexane); $[\alpha]_D^{20}$ = +18.6 (*c* 0.976, $CHCl_3$); **IR** (ATR) ν_{max} 2941, 2865, 1715 (C=O ketone), 1476, 1462, 1108, 1069, 882, 739, 689 cm^{-1}; **^{1}H NMR** (400 MHz, $CDCl_3$) δ 7.26–7.22 (2H, m, Ar-H), 7.16–7.10 (3H, m, Ar-H), 3.81 (1H, dd, *J* = 9.9, 6.0 Hz, H-12), 3.77 (1H, dd, *J* = 9.9, 6.2 Hz, H-12), 2.89 (1H, ddd, *J* = 5.0, 5.0, 4.0 Hz, H-6), 2.69 (1H, dd, *J* = 18.0, 4.0 Hz, H-7), 2.46 (1H, dddd, *J* = 9.3, 9.3, 5.0, 5.0 Hz, H-5), 2.41–2.30 (2H, m, H-2), 2.20–2.11 (3H, m, H-10 + H-7), 1.99 (3H, s, H-11), 1.97–1.89 (2H, m, H-3), 1.81–1.73 (1H, m, H-4), 1.10–0.99 (21H, m, $OSi(CH(CH_3)_2)_3$) ppm; **^{13}C NMR** (100 MHz, $CDCl_3$) δ 212.6 (C-1), 140.2 (C-9), 136.6 (C-Ar), 129.0 (2CH-Ar), 128.0 (2CH-Ar), 125.4 (CH-Ar), 121.2 (C-8), 65.3 (C-12), 45.5 (C-6), 39.0 (C-4), 37.6 (C-5), 37.5 (C-2), 35.2 (C-10), 31.3 (C-7), 25.6 (C-3), 21.4 (C-11), 18.2 ($OSi(CH(\underline{C}H_3)_2)_3$), 12.0 ($OSi(\underline{C}H(CH_3)_2)_3$) ppm; **HRMS** (ESI) *m/z* cald for $C_{27}H_{42}NaO_2SSi$ (M+Na$^+$) 481.2567, found 481.2573.

nOe data for 4

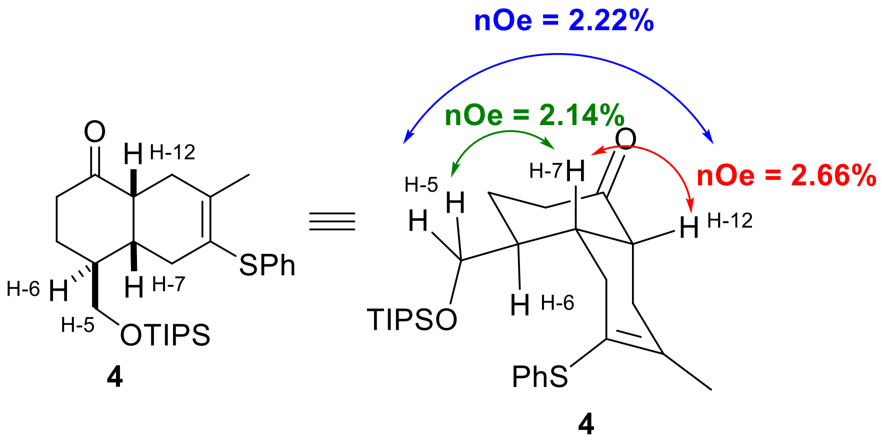

Figure S1 – nOe correlations for 4

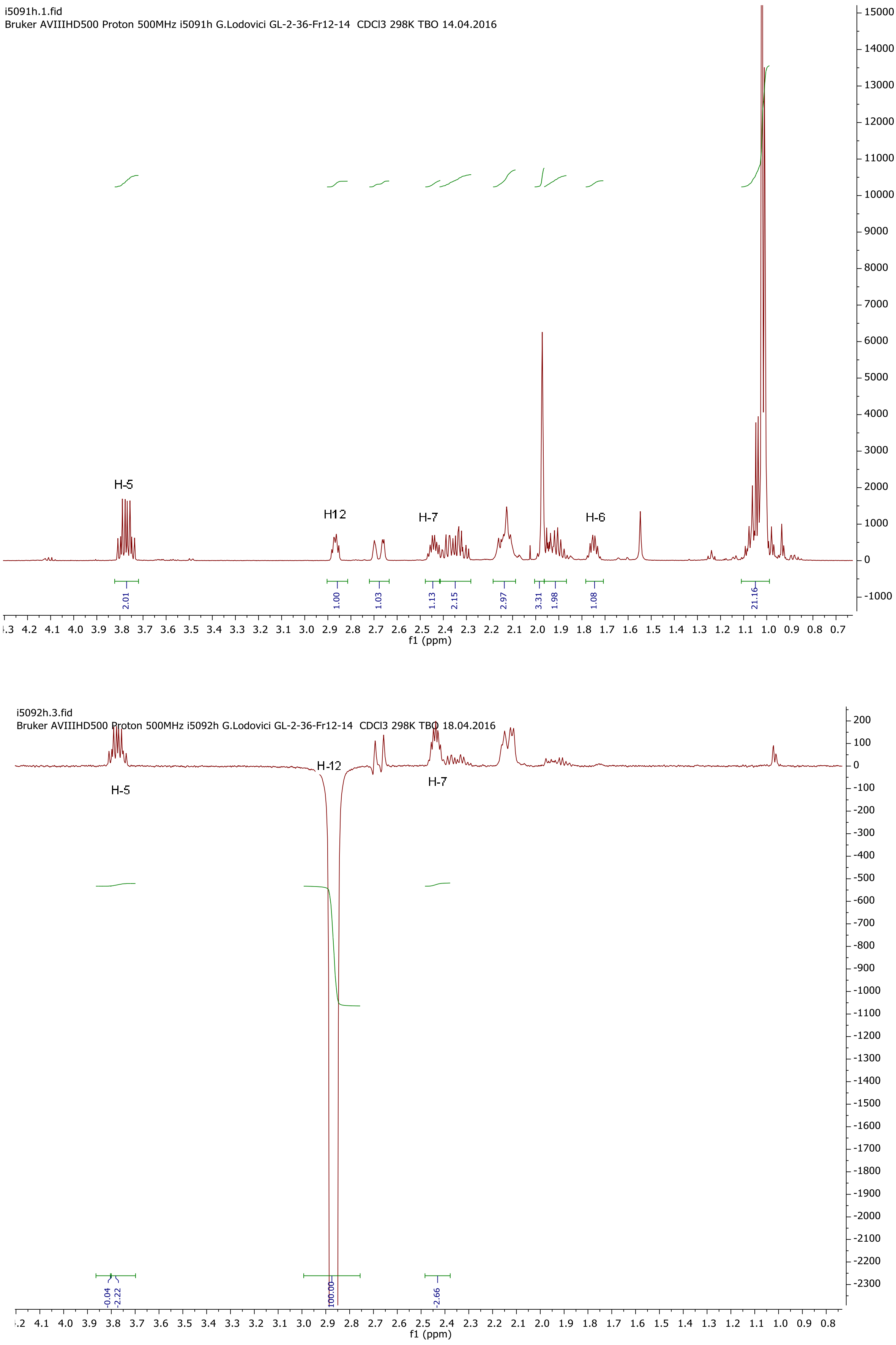

Figure S2. The ^{1}H NMR spectrum of *cis*-decalin **4** and the nOe analysis of H-12.

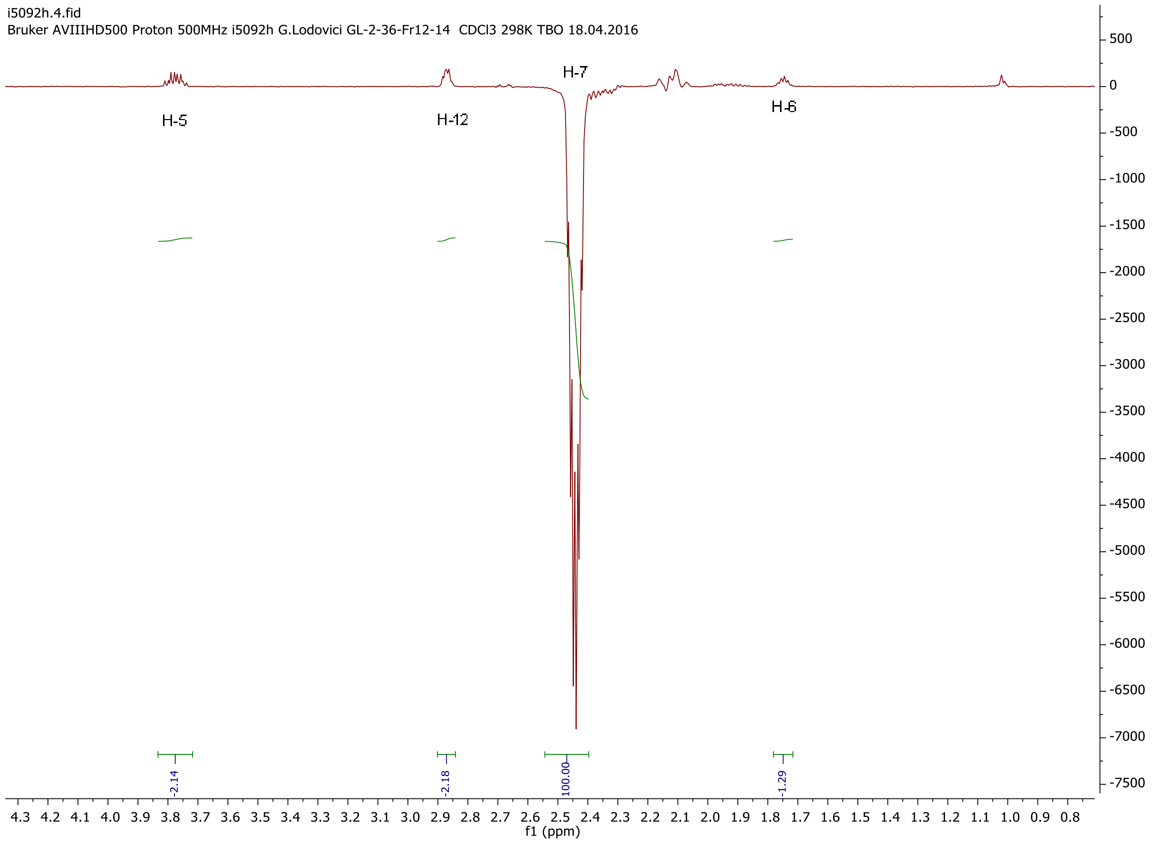

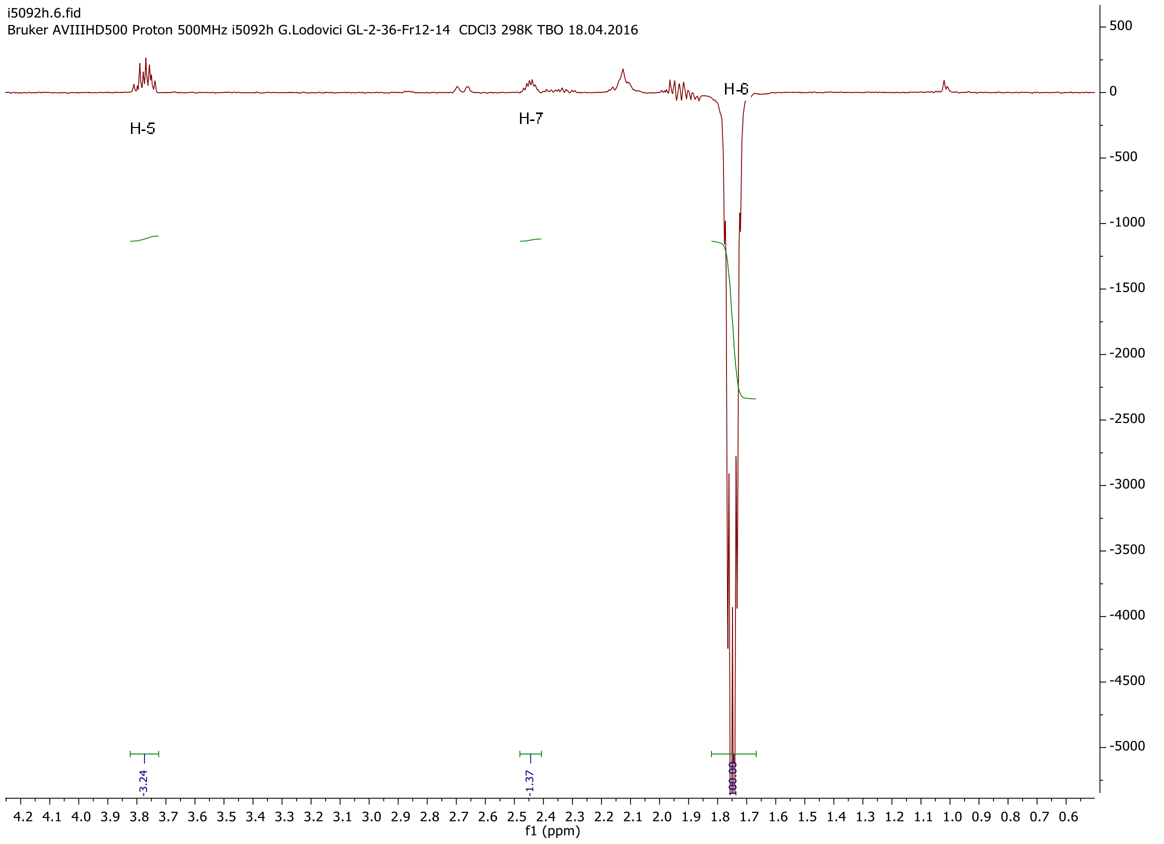

Figure S3. The nOe analysis of H-7 and H-6 for decalin **4**.

(4*S*,4a*R*,8a*S*)-7-Methyl-6-(phenylthio)-4-(((triisopropylsilyl)oxy)methyl)-3,4,4a,5,8,8a-hexahydronaphthalen-1(*2H*)-one (16)

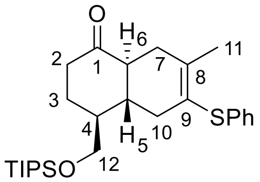

To a solution of *cis*-decalin **4** (1.55 g, 3.38 mmol, 1.0 equiv) in CH_2Cl_2 (17 mL) was added a 1.0 M solution of $EtAlCl_2$ in hexane (0.845 mL, 0.845 mmol, 0.25 equiv) and the reaction mixture was stirred under N_2 atmosphere at room temperature for 3 days. The reaction mixture was then quenched with Rochelle's salt 10% aqueous solution (20 mL) and stirred vigorously overnight. The organic layer was separated, and the aqueous layer was extracted with CH_2Cl_2 (3 x 20 mL). The combined organic layers were washed with brine, dried over anhydrous Na_2SO_4 and concentrated *in vacuo*. The crude residue was purified by silica gel flash column chromatography (5% Et_2O/hexane) to yield the desired *trans*-decalin **16** as a yellow oil (1.25 g, 81% yield): $\boldsymbol{R_f}$ = 0.07 (5% Et_2O/hexane); $\mathbf{[\alpha]_D^{20}}$ = +65.6 (*c* 0.231, $CHCl_3$); **IR** (ATR) ν_{max} 2941, 2865, 1713 (C=O ketone), 1476, 1120, 1070, 883, 740, 687 cm^{-1}; ^{1}**H NMR** (400 MHz, $CDCl_3$) δ 7.26–7.21 (2H, m, Ar-H), 7.18–7.10 (3H, m, Ar-H), 3.64 (1H, dd, *J* = 9.9, 3.1 Hz, H-12), 3.58 (1H, dd, *J* = 9.9, 5.0 Hz, H-12), 2.55–2.40 (4H, m, H-2 + H-10 + H-7), 2.39–2.32 (2H, m, H-6 + H-7), 2.17–2.08 (2H, m, H-3 + H-10), 1.99 (3H, s, H-11), 1.85–1.70 (2H, m, H-3 + H-5), 1.68–1.60 (1H, m, H-4), 1.00–0.90 (21H, m, $OSi(CH(CH_3)_2)_3$) ppm; ^{13}**C NMR** (100 MHz, $CDCl_3$) δ 211.8 (C-1), 140.7 (C-9), 136.2 (C-Ar), 129.0 (2CH-Ar), 128.3 (2CH-Ar), 125.6 (CH-Ar), 121.6 (C-8), 64.2 (C-12), 49.1 (C-6), 44.8 (C-4), 41.7 (C-5), 41.3 (C-2), 38.1 (C-10), 32.4 (C-7), 30.0 (C-3), 21.6 (C-11), 18.1 ($OSi(CH(\underline{C}H_3)_2)_3$), 12.0 ($OSi(\underline{C}H(CH_3)_2)_3$) ppm; **HRMS** (ESI) *m/z* cald for $C_{27}H_{42}NaO_2SSi$ requires ($M+Na^+$) 481.2567, found 481.2567.

(*4S*,4a*R*,8a*S*)-7-Methyl-4-(((triisopropylsilyl)oxy)methyl)-3,4,4a,5,8,8a-hexahydronaphthalen-1(2*H*)-one (17)

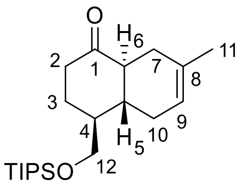

To a solution of *trans*-decalin **16** (0.34 g, 0.74 mmol, 1.0 equiv) in acetone (74 mL) was added an excess of unwashed Raney Nickel (50% in H_2O, 30.0 equiv) and the reaction mixture was stirred under a H_2 atmosphere (balloon) at room temperature for 1 hour. After this time, the reaction was filtered through Celite and washed with CH_2Cl_2. The filtrate was dried over anhydrous Na_2SO_4 and concentrated *in vacuo*. The crude residue was purified by silica gel flash column chromatography (2% EtOAc/hexane) to deliver the title compound **17** as a light yellow oil (224 mg, 86% yield): $\boldsymbol{R_f}$ = 0.10 (2% EtOAc/hexane); $\boldsymbol{[\alpha]_D^{20}}$ = +46.1 (*c* 1.21, $CHCl_3$); **IR** (ATR) ν_{max} 2941, 2865, 1713 (C=O ketone), 1462, 1118, 1098, 882, 787, 682 cm^{-1}; **^{1}H NMR** (400 MHz, $CDCl_3$) δ 5.34–5.28 (1H, m, H-9), 3.82 (1H, dd, *J* = 9.8, 2.4 Hz, H-12), 3.64 (1H, dd, *J* = 9.8, 5.3 Hz, H-12), 2.50–2.37 (2H, m, H-2), 2.36–2.11 (4H, m, H-6 + H-7 + H-3 + + H-10), 2.07–2.02 (1H, m, H-7), 1.92–1.81 (1H, m, H-10), 1.67 (3H, s, H-11), 1.65–1.55 (3H, m, H-4 + H-3 + H-5), 1.08–1.02 (21H, m, $OSi(CH(CH_3)_2)_3$) ppm; **^{13}C NMR** (100 MHz, $CDCl_3$) δ 212.6 (C-1), 133.1 (C-8), 119.2 (C-9), 64.7 (C-12), 49.4 (C-6), 45.1 (C-4), 41.4 (C-2), 40.2 (C-5), 31.7 (C-10), 30.2 (C-7), 29.7 (C-3), 23.6 (C-11), 18.2 ($OSi(CH(\underline{C}H_3)_2)_3$), 12.1 ($OSi(\underline{C}H(CH_3)_2)_3$) ppm; **HRMS** (ESI) *m/z* cald for $C_{21}H_{38}NaO_2Si$ ($M+Na^+$) 373.2533, found 373.2534.

13-(2,4-Dinitrophenyl)-14-((12*S, 7*R**, 6*S**)-10-methyl-6-(((triisopropylsilyl)oxy)methyl)-15,6,7,8,11,12-hexahydronaphthalen-13(2H)-ylidene)hydrazine (19)**

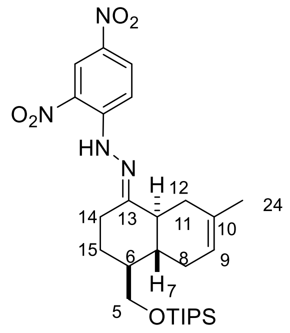

To a solution of *trans*-decalin **17** (25 mg, 0.070 mmol) and 3Å molecular sieves in dry methanol (4.0 mL), 2,4-dinitrophenylhydrazine **18** (28 mg, 0.14 mmol) and glacial acetic acid (0.50 mL) were added. The reaction was stirred at 50 °C for 16 hours. After this time, the reaction was

quenched with saturated aqueous solution of $NaHCO_3$ (5 mL) and the organic layer was extracted with CH_2Cl_2 (3 × 10 mL) dried with Na_2SO_4, filtered and concentrated *in vacuo*. The crude product was purified by silica gel flash column chromatography (5–20% EtOAc in hexane) to yield the title compound **19** as an orange solid (26 mg, 70% yield). Crystallization method: slow evaporation of solvent from a solution of **19** in a minimum amount of $CHCl_3$. **Melting point** = 71–73 °C. **R*f*** = 0.61 (10% EtOAc/hexane). **IR** (ATR): ν_{max} 3006, 2942, 2864, 1617 (C=N), 1518, 1422, 1333, 1119, 1092 cm^{-1}. **^{1}H NMR** (400 MHz, $CDCl_3$): δ 11.29 (1H, s, N-H), 9.15 (1H, d, *J* = 2.8 Hz, Ar-H), 8.34 (1H, dd, *J* = 9.6, 2.8 Hz, Ar-H), 8.00 (1H, d, *J* = 9.6 Hz, Ar-H), 5.41–5.37 (1H, m, H-9), 3.83 (1H, dd, *J* = 10.1, 2.8 Hz, H-5), 3.64 (1H, dd, *J* = 10.1, 5.5 Hz, H-5), 3.01 (1H, ddd, *J* = 14.2, 3.2, 3.2 Hz, H-14), 2.45–2.30 (4H, m, H-12 + H-11 + H-8), 2.29–2.20 (1H, m, H-14), 2.18–2.03 (1H, m, H-15), 1.94–1.82 (1H, m, H-8), 1.78 (3H, s, H-24), 1.57–1.49 (3H, m, H-6 + H-7 + H-15), 1.09–1.02 (21H, m, $OSiCH(CH_3)_2$) ppm. **^{13}C NMR** (400 MHz, $CDCl_3$): δ 162.1 (C=N), 145.6 (C-10), 137.5 (Ar), 132.9 (Ar), 130.0 (Ar), 128.9 (Ar-H), 123.4 (Ar-H), 119.2 (C-9), 116.4 (Ar-H), 64.6 (C-5), 45.2 (C-6), 44.0 (C-12), 39.8 (C-7), 31.7 (C-8), 31.5 (C-11), 28.6 (C-15), 26.5 (C-14), 23.7 (C-24), 18.0 ($OSiCH(\underline{C}H_3)_2$), 11.9 ($OSi\underline{C}H(CH_3)_2$) ppm. **MS** (ESI): m/z 529 ($M-H^+$); HRMS: found: ($M-H^+$) 529.2847. $C_{27}H_{41}N_4O_5Si$ requires ($M-H^+$) 529.2852.

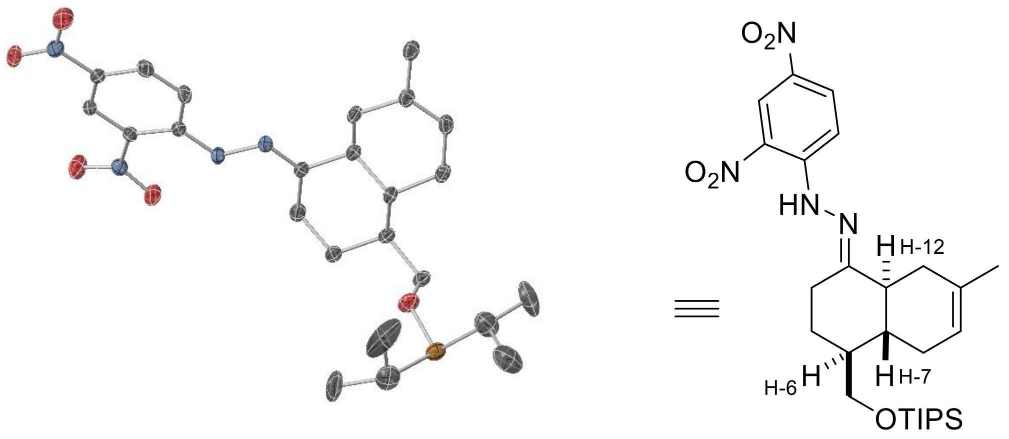

19

Figure S4. Single crystal X-ray diffraction of hydrazone **19** - CCDC = 2341814. Single crystal X-ray diffraction structure visualised in CrystalMaker v11.0.02: thermal ellipsoids set at 50% probability level, H-atoms omitted for clarity; oxygen atoms in red, carbon in grey, nitrogen in blue and silicon in light orange. The disorder in the OTIPS group is not shown

Triethyl(((4*S*,4a*R*,8a*S*)-7-methyl-4-(((triisopropylsilyl)oxy)methyl)-3,4,4a,5,8,8a-hexahydronaphthalen-1-yl)oxy)silane (20)

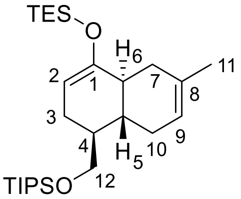

To a solution of freshly distilled diisopropylamine (1.27 mL, 9.048 mmol, 1.2 equiv) in THF (9.1 mL) at –78 °C was added *n*-BuLi (1.6 M solution, 5.71 mL, 9.048 mmol, 1.2 equiv) dropwise. The solution was stirred under N_2 atmosphere at –78 °C for 45 minutes before a solution of ketone **17** (2.61 g, 7.54 mmol, 1.0 equiv) in THF (29 mL) was added. The mixture was stirred at –78 °C for 1 hour and then added freshly distilled triethylsilyl chloride (1.24 mL, 9.80 mmol, 1.3 equiv). The reaction mixture was stirred at –78 °C for a further 30 minutes before being warmed to room temperature for 30 minutes. The mixture was then quenched with H_2O (30 mL) and diluted with Et_2O (30 mL). The organic layer was separated, and the aqueous layer was extracted with Et_2O (3 x 30 mL). The organic layers were washed with brine, dried over anhydrous Na_2SO_4 and concentrated *in vacuo*. The crude residue was purified by silica gel flash column chromatography (2% Et_2O/hexane) to yield title compound **20** as a yellow oil (3.16 g, 91% yield): $\boldsymbol{R_f}$ = 0.34 (2% Et_2O/hexane); $\mathbf{[\alpha]_D^{20}}$ = +73.7 (*c* 0.379, $CHCl_3$); **IR** (ATR) ν_{max} 2951, 2867, 1666, 1462, 1200, 1113, 1013, 882, 729 cm^{-1}; **^{1}H NMR** (400 MHz, $CDCl_3$) δ 5.39–5.33 (1H, m, H-9), 4.83 (1H, ddd, *J* = 5.7, 1.7, 1.7 Hz, H-2), 3.76 (1H, dd, *J* = 9.6, 3.4 Hz, H-12), 3.58 (1H, dd, *J* = 9.6, 6.2 Hz, H-12), 2.28 (1H, dd, *J* = 15.2, 4.0 Hz, H-7), 2.23–1.96 (4H, m, H-10 + H-3 + H-6), 1.80–1.69 (2H, m, H-3 + H-7), 1.67 (3H, s, H-11), 1.52–1.39 (2H, m, H-4 + H-5), 1.07–1.03 (21H, m, $OSi(CH(CH_3)_2)_3$), 0.98 (9H, t, *J* = 7.8 Hz, ($OSi(CH_2\underline{CH_3})_3$)), 0.68 (6H, q, *J* = 7.8 Hz, ($OSi(\underline{CH_2}CH_3)_3$) ppm; **^{13}C NMR** (100 MHz, $CDCl_3$) δ 152.1 (C-1), 134.2 (C-8), 120.5 (C-9), 102.3 (C-2), 65.3 (C-12), 41.5 (C-6), 41.3 (C-4), 38. (C-5), 34.3 (C-10), 30.4 (C-7), 27.9 (C-3), 23.8 (C-11), 18.2 ($OSi(CH(\underline{C}H_3)_2)_3$), 12.1 ($OSi(\underline{C}H(CH_3)_2)_3$), 7.0 ($OSi(CH_2\underline{C}H_3)_3$), 5.2 ($OSi(\underline{C}H_2CH_3)_3$) ppm; **HRMS** (APCI) *m/z* cald for $C_{27}H_{53}O_2Si$ ($M+H^+$) 465.3579, found 465.3586.

(4*S*,4a*R*,8a*S*)-7-Methyl-4-(((triisopropylsilyl)oxy)methyl)-4a,5,8,8a-tetrahydronaphthalen-1(4*H*)-one (21)

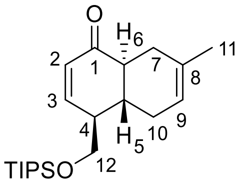

To a solution of TES-enol ether **20** (72 mg, 0.15 mmol, 1.0 equiv) in DMSO (1.5 mL) was added IBX (105 mg, 0.375 mmol, 2.5 equiv). The reaction mixture was stirred under N_2 atmosphere at 60 °C for 3 days before being cooled to room temperature. The mixture was quenched with a saturated aqueous solution of $NaHCO_3$ (10 mL) and diluted with Et_2O (10 mL). The organic layer was separated, and the aqueous layer was extracted with Et_2O (3 x 10 mL). The combined organic layers were washed with brine, dried over anhydrous Na_2SO_4 and concentrated *in vacuo*. The crude residue was purified by silica gel flash column chromatography (2% Et_2O/hexane) to produce the enone *trans*-decalin **5** as a light yellow oil (32 mg, 59% yield): $\boldsymbol{R_f}$ = 0.14 (5% Et_2O/hexane); $\boldsymbol{[\alpha]_D^{20}}$ = +125.2 (*c* 0.328, $CHCl_3$); **IR** (ATR) ν_{max} 2942, 2866, 1674 (C=O ketone), 1463, 1393, 1114, 882, 783, 683 cm^{-1}; **^{1}H NMR** (400 MHz, $CDCl_3$) δ 7.07 (1H, dd, *J* = 10.2, 1.9 Hz, H-3), 6.07 (1H, dd, *J* = 10.2, 2.8 Hz, H-2), 5.37–5.29 (1H, m, H-9), 3.97 (1H, dd, *J* = 9.7, 4.2 Hz, H-12), 3.65 (1H, dd, *J* = 9.7, 7.2 Hz, H-12), 2.45–2.26 (4H, m, H-7 + H-4 + H-6), 2.10–1.81 (3H, m, H-5 + H-10), 1.69 (3H, s, H-11), 1.10–1.02 (21H, m, $OSi(CH(CH_3)_2)_3$) ppm; **^{13}C NMR** (100 MHz, $CDCl_3$) δ 201.3 (C-1), 152.2 (C-3), 133.3 (C-8), 129.2 (C-2), 118.9 (C-9), 63.8 (C-12), 46.1 (C-6), 45.9 (C-4), 36.3 (C-5), 31.2 (C-7), 30.3 (C-10), 23.6 (C-11), 18.1 ($OSi(CH(\underline{C}H_3)_2)_3$), 12.0 ($OSi(\underline{C}H(CH_3)_2)_3$) ppm; **HRMS** (ESI) *m/z* cald for $C_{21}H_{36}NaO_2Si$ ($M+Na^+$) 371.2377, found 371.2370.

(4*S*,4a*R*,8a*S*)-4-(Hydroxymethyl)-7-methyl-4a,5,8,8a-tetrahydronaphthalen-1(4*H*)-one (22)

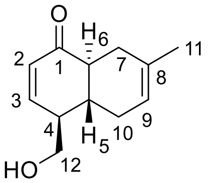

To a solution of enone *trans*-decalin **21** (0.29 g, 0.83 mmol, 1.0 equiv) in THF (8.3 mL) was added a 1.0 M solution of TBAF in THF (1.66 mL, 1.66 mmol, 2.0 equiv). The reaction mixture was stirred under N_2 atmosphere at room temperature for 2 hours. The mixture was then quenched with H_2O (20 mL) and diluted with EtOAc (20 mL). The organic layer was separated,

and the aqueous layer was extracted with EtOAc (3 x 20 mL). The combined organic layers were washed with brine, dried over anhydrous Na_2SO_4 and concentrated *in vacuo*. The crude residue was purified by silica gel flash column chromatography (30–40% EtOAc/hexane) to afford alcohol **22** as a light orange oil (157 mg, 98% yield): $\boldsymbol{R_f}$ = 0.21 (40% EtOAc/hexane); $[\alpha]_D^{20}$ = +155.8 (*c* 0.451, $CHCl_3$); **IR** (ATR) ν_{max} 3413 (O-H), 2915, 2883, 1661 (C=O ketone), 1437, 1394, 1190, 1044, 780 cm^{-1}; ^{1}**H NMR** (400 MHz, $CDCl_3$) δ 7.01 (1H, dd, *J* = 10.2, 2.0 Hz, H-3), 6.10 (1H, ddd, *J* = 10.2, 1.8, 1.8 Hz, H-2), 5.40–5.28 (1H, m, H-9), 3.93 (1H, dd, *J* = 10.6, 4.1 Hz, H-12), 3.68 (1H, dd, *J* = 10.6, 6.4 Hz, H-12), 2.49–2.26 (4H, m, H-6 + H-4 + H-10), 2.09–1.88 (3H, m, H-5 + H-7), 1.69 (3H, s, H-11) ppm; ^{13}**C NMR** (100 MHz, $CDCl_3$) δ 201.2 (C-1), 151.5 (C-3), 133.3 (C-8), 130.0 (C-2), 118.8 (C-9), 63.0 (C-12), 45.9 (C-6), 45.5 (C-4), 36.0 (C-5), 31.1 (C-7), 30.3 (C-10), 23.5 (C-11) ppm; **HRMS** (ESI) *m/z* cald for $C_{12}H_{16}NaO_2$ (M+Na$^+$) 215.1043, found 215.1045.

Ethyl (((1*S*,4a*S*,8a*R*)-6-methyl-4-oxo-1,4,4a,5,8,8a-hexahydronaphthalen-1-yl)methyl) malonate (23)

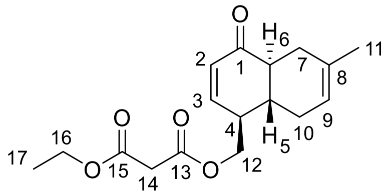

To a solution of alcohol **22** (0.18 g, 0.93 mmol, 1.0 equiv) and Et_3N (0.259 mL, 1.86 mmol, 2.0 equiv) in CH_2Cl_2 (4.7 mL) at 0 °C was slowly added ethyl malonyl chloride (0.179 mL, 1.40 mmol, 1.5 equiv). The reaction mixture was stirred under N_2 atmosphere at 0 °C initially and allowed to rise to room temperature over 2 hours. The mixture was then diluted with CH_2Cl_2 (30 mL) and washed with a saturated aqueous solution of $NaHCO_3$ (3 x 10 mL) and brine. The organic layer was dried over anhydrous Na_2SO_4 and concentrated *in vacuo*. The crude residue was purified by silica gel flash column chromatography (20–40% EtOAc/hexane) to give malonate **23** as a light yellow oil (243 mg, 85% yield): $\boldsymbol{R_f}$ = 0.49 (40% EtOAc/hexane); $[\alpha]_D^{20}$ = +127.7 (*c* 0.315, $CHCl_3$); **IR** (ATR) ν_{max} 2920, 1734 (C=O ester), 1673 (C=O ketone), 1330, 1268, 1149, 1033 cm^{-1}; ^{1}**H NMR** (400 MHz, $CDCl_3$) δ 6.85 (1H, dd, *J* = 10.2, 2.1 Hz, H-3), 6.09 (1H, dd, *J* = 10.2, 2.8 Hz, H-2), 5.40–5.30 (1H, m, H-9), 4.44 (1H, dd, *J* = 11.2, 3.8 Hz, H-12), 4.20 (1H, dd, *J* = 11.2, 6.6 Hz, H-12), 4.18 (2H, q, *J* = 7.2 Hz, H-16), 3.39 (2H, s, H-14), 2.59–2.50 (1H, m, H-4), 2.46–2.32 (3H, m, H-6 + H-10), 2.10–1.85 (3H, m, H-7 + H-5), 1.69 (3H, s, H-11), 1.26 (3H, t, *J* = 7.2 Hz, H-17) ppm; ^{13}**C NMR** (100 MHz, $CDCl_3$) δ 200.4 (C-1), 166.7 (C-13), 166.4 (C-15),

149.6 (C-3), 130.3 (C-8), 130.2 (C-2), 118.6 (C-9), 65.2 (C-12), 61.8 (C-16), 46.0 (C-6), 42.6 (C-4), 41.6 (C-14), 36.6 (C-5), 30.9 (C-7), 30.1 (C-10), 23.5 (C-11), 14.2 (C-17) ppm; **HRMS** (ESI) *m/z* cald for $C_{17}H_{22}NaO_5$ (M+Na$^+$) 329.1359, found 329.1360.

Ethyl (4*S*,4a*S*,6a*S*,10a*R*,10b*R*)-8-methyl-3,6-dioxo-3,4,4a,5,6,6a,7,10,10a,10b-decahydro-1*H*-benzo[*h*]isochromene-4-carboxylate (24)

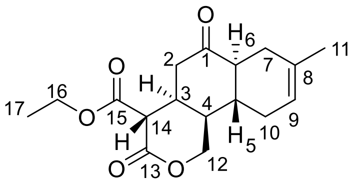

To a mixture of malonate **23** (46 mg, 0.15 mmol) and La(O-*i*-Pr)$_3$ (53 mg, 0.16 mmol, 1.0 equiv) in THF (1.2 mL), was stirred under N_2 atmosphere at room temperature for 10 minutes. Freshly distilled *i*-Pr$_2$NEt (0.052 mL, 0.31 mmol, 2.0 equiv) was then added. The reaction mixture was stirred at 40 °C for a further 7 hours. The mixture was then cooled to room temperature, quenched with saturated aqueous solution of $NaHCO_3$ (3 mL) and diluted with EtOAc (3 mL). The organic layer was separated, and the aqueous layer was extracted with EtOAc (5 × 3 mL). The combined organic layers were washed with brine, dried over anhydrous Na_2SO_4 and concentrated *in vacuo*. The crude product was purified by silica gel flash column chromatography (Et_2O:CH_2Cl_2:hexane = 15:40:45) to the title compound **24** (35 mg, 75% yield) as a white solid: $\boldsymbol{R_f}$ = 0.23 (40% EtOAc/hexane); $\boldsymbol{[\alpha]_D^{20}}$ = +91.8 (*c* 0.257, $CHCl_3$); **IR** (ATR) ν_{max} 2961, 2916, 1729 (C=O ester), 1437, 1235, 1154, 1035 cm^{-1}. ^{1}**H NMR** (400 MHz, $CDCl_3$) δ 5.39–5.31 (1H, m, H-9), 4.46 (2H, d, *J* = 3.5 Hz, H-12), 4.27 (2H, qd, *J* = 7.2, 1.6 Hz, H-16), 3.27 (1H, d, *J* = 11.4 Hz, H-14), 3.10 (1H, dddd, *J* = 11.4, 5.5, 5.5, 5.0 Hz, H-3), 2.66 (1H, dd, *J* = 14.6, 5.5 Hz, H-2), 2.44–2.31 (3H, m, H-6 + H-10 + H-2), 2.20–2.10 (3H, m, H-4 + H-7), 1.98–1.85 (1H, m, H-10), 1.79 (1H, dddd, *J* = 11.2, 11.2, 11.2, 4.8 Hz, H-5), 1.68 (3H, s, H-11), 1.31 (3H, t, *J* = 7.2 Hz, H-17) ppm; ^{13}**C NMR** (100 MHz, $CDCl_3$) δ 209.1 (C-1), 168.3 (C-15), 166.3 (C-13), 133.4 (C-8), 118.3 (C-9), 69.1 (C-12), 62.5 (C-16), 50.7 (C-14), 48.8 (C-6), 43.7 (C-2), 38.9 (C-4), 36.5 (C-3), 34.4 (C-5), 31.5 (C-10), 29.7 (C-7), 23.4 (C-11), 14.2 (C-17) ppm; **HRMS** (ESI) *m/z* cald for $C_{17}H_{22}NaO_5$ (M+Na$^+$) 329.1359, found 329.1363.

The *syn*-relationship between H-3 and H-4 required for the natural product core is supported by n.O.E data- (Figures S5–7). The relationship between H-3 and H-14 was confirmed to be the *anti*-relationship by the vicinal coupling constant of 11.4 Hz (J_{axial}) as shown in Figure S5.

Furthermore, the configuration of five stereogenic centres in tricyclic lactone **24** was further supported by single crystal X-ray diffraction data (Figure S6).

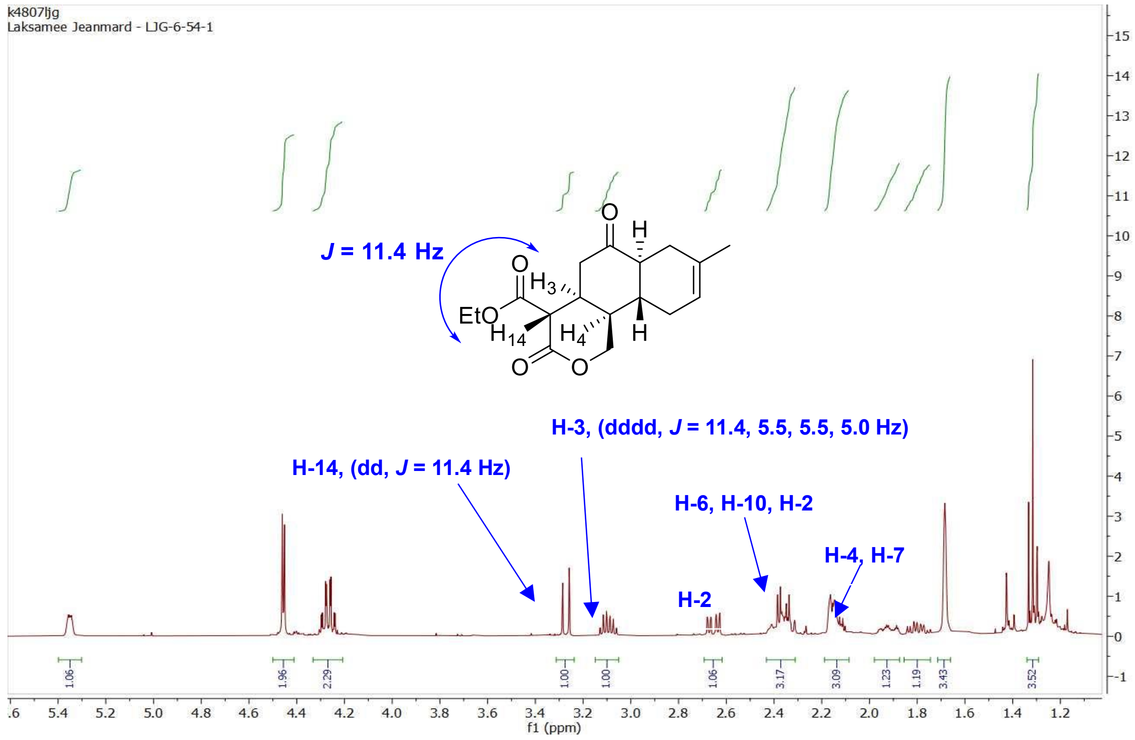

Figure S5: *The* ^{1}H *NMR spectrum of tricyclic lactone* ***24***

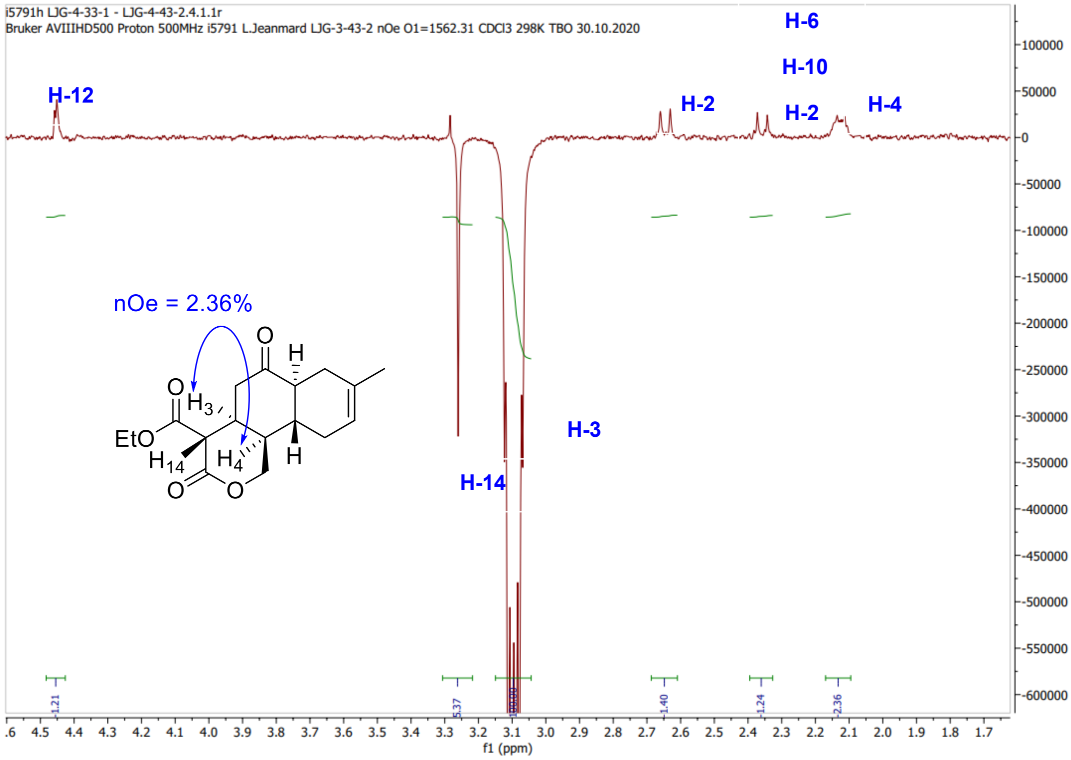

Figure S6: *The nOe analysis of H-3 of tricyclic lactone* ***24***

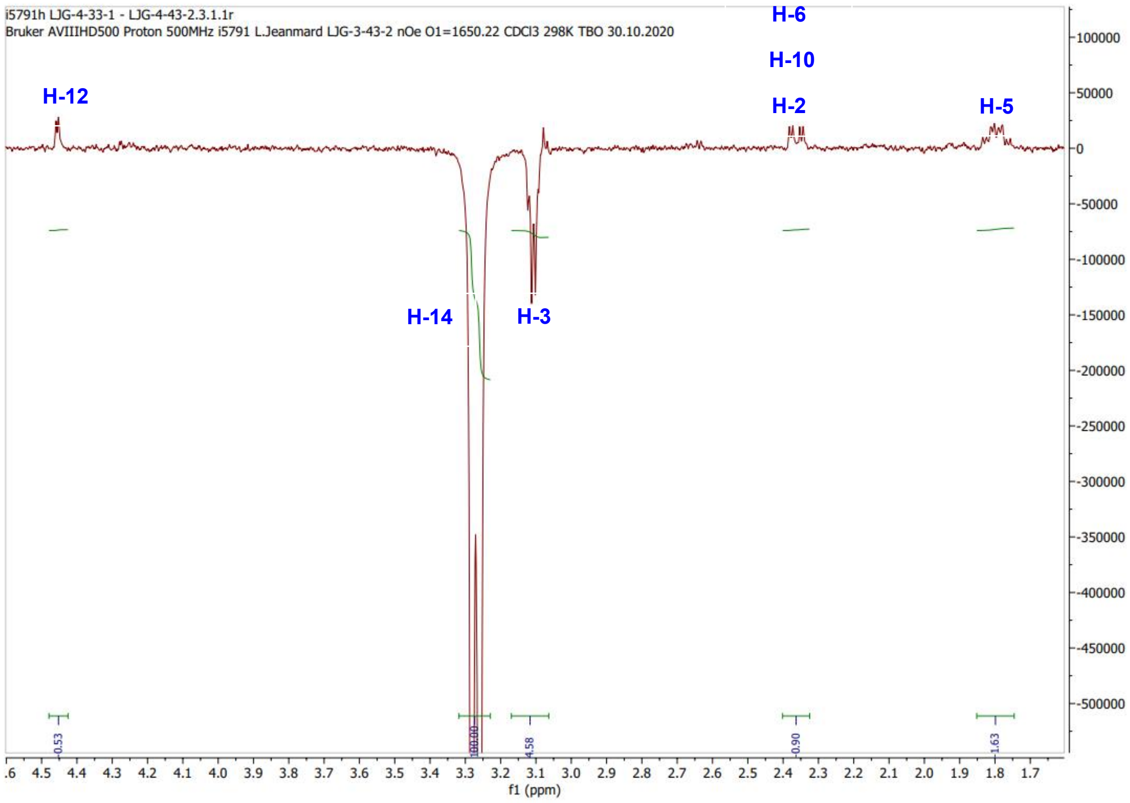

Figure S7: *The nOe analysis of H-14 of tricyclic lactone **24***

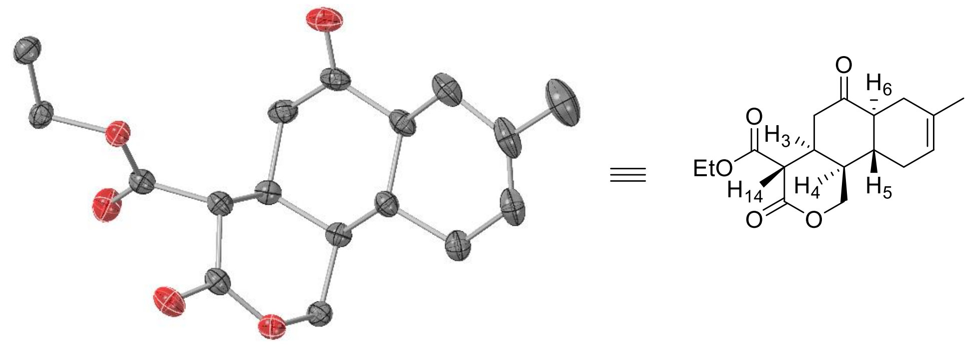

Figure S8: *The single crystal X-ray diffraction of tricyclic lactone **24.*** **CCDC =** 2341815. Single crystal X-ray diffraction structure visualised in CrystalMaker v11.0.02: thermal ellipsoids set at 50% probability level, H-atoms omitted for clarity; oxygen atoms in red and carbon in grey.

Ethyl (4*R*,4a*S*,6a*S*,10a*R*,10b*R*)-4,8-dimethyl-3,6-dioxo-3,4,4a,5,6,6a,7,10,10a,10b-decahydro-1*H*-benzo[*h*]isochromene-4-carboxylate (25)

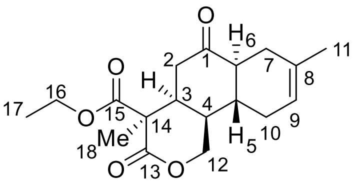

To a solution of tricyclic lactone **24** (14 mg, 0.045 mmol) in THF (1.5 mL) at 0 °C, was added NaH (4 mg, 0.07 mmol, 1.5 equiv, 60% in mineral oil) in one portion. The reaction mixture was stirred at 0 °C for 5 minutes, and then methyl iodide (0.01 mL, 0.09 mmol, 2.0 equiv) was added. The reaction mixture was warmed to room temperature and stirred for 6 hours before being quenched with saturated aqueous solution of NH_4Cl (3 mL) and diluted with EtOAc (3 mL). The organic layer was separated, and the aqueous layer was extracted with EtOAc (3 × 5 mL). The combined organic layers were washed with brine, dried over anhydrous Na_2SO_4 and concentrated *in vacuo*. The crude product was purified by silica gel flash column chromatography (20–40% EtOAc/hexane) to deliver the title compound **25** as a yellow oil (10 mg, 71% yield): $\boldsymbol{R_f}$ = 0.23 (40% EtOAc/hexane); $[\boldsymbol{\alpha}]_D^{20}$ = +94.9 (*c* 0.889, $CHCl_3$); **IR** (ATR) ν_{max} 2915, 1730 (C=O ester), 1711 (C=O ester), 1675 (C=O ketone), 1448, 1369, 1224, 1113, 1023 cm^{-1}; **^{1}H NMR** (400 MHz, $CDCl_3$) δ 5.41–5.29 (1H, m, H-9), 4.40 (1H, dd, *J* = 12.0, 4.2 Hz, H-12), 4.31–4.25 (1H, m, H-12), 4.24 (1H, dq, *J* = 10.8, 7.2 Hz, H-16), 4.08 (1H, dq, *J* = 10.8, 7.2 Hz, H-16), 3.09 (1H, dd, *J* = 15.0, 7.4 Hz, H-2), 2.55–2.40 (2H, m, H-2 + H-3), 2.32–2.17 (2H, m, H-7 + H-10), 2.17–2.06 (3H, m, H-6 + H-5 + H-4), 2.06–1.95 (1H, m, H-7), 1.86–1.74 (1H, m, H-10), 1.68 (3H, s, H-11), 1.55 (3H, s, H-18), 1.26 (3H, t, *J* = 7.2 Hz, H-17) ppm; **^{13}C NMR** (100 MHz, $CDCl_3$) δ 210.9 (C-1), 171.3 (C-13), 170.7 (C-15), 133.6 (C-8), 118.9 (C-9), 68.4 (C-12), 62.2 (C-16), 52.2 (C-14), 48.6 (C-6), 40.5 (C-5), 38.8 (C-2), 38.2 (C-3), 34.0 (C-4), 31.0 (C-7), 30.8 (C-10), 23.5 (C-18), 23.4 (C-11), 13.9 (C-17) ppm; **HRMS** (ESI) *m/z* cald for $C_{18}H_{24}NaO_5$ (M+Na^+) 343.1516, found 343.1510.

nOe data for tricyclic lactone 25

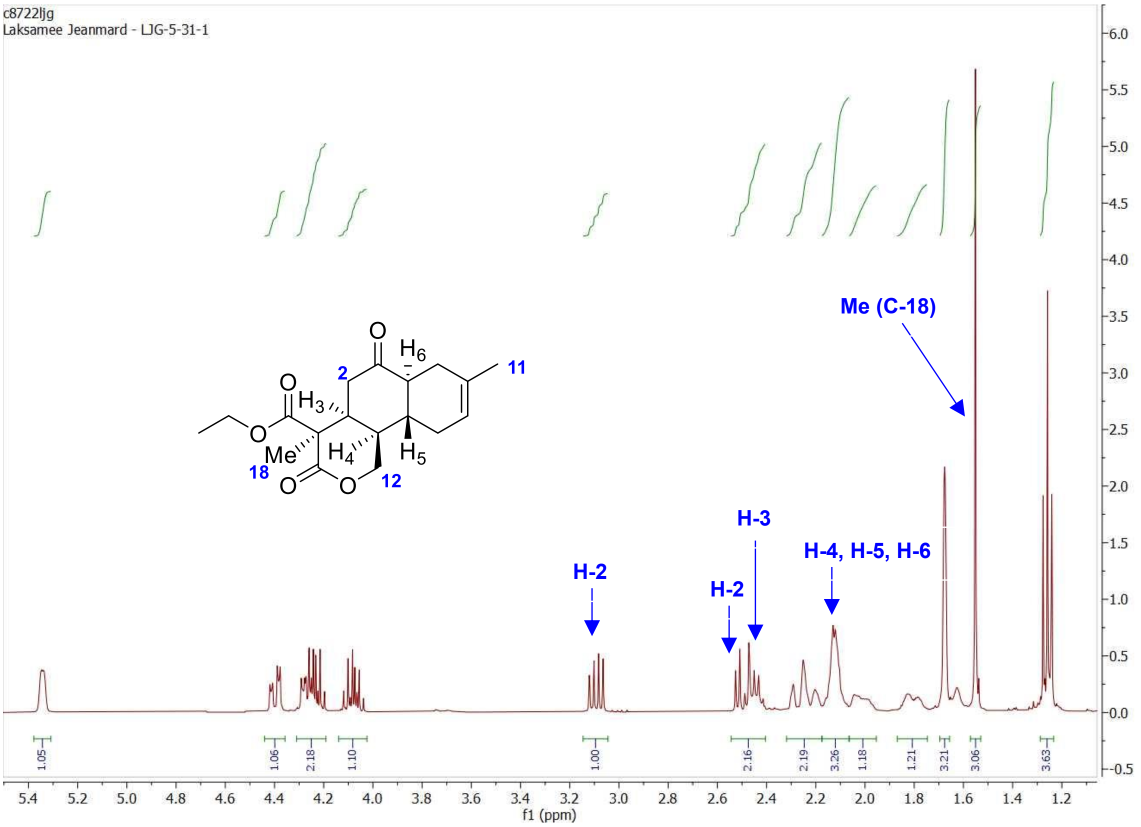

Figure S9: *The* ^{1}*H NMR spectrum of tricyclic lactone* ***25***

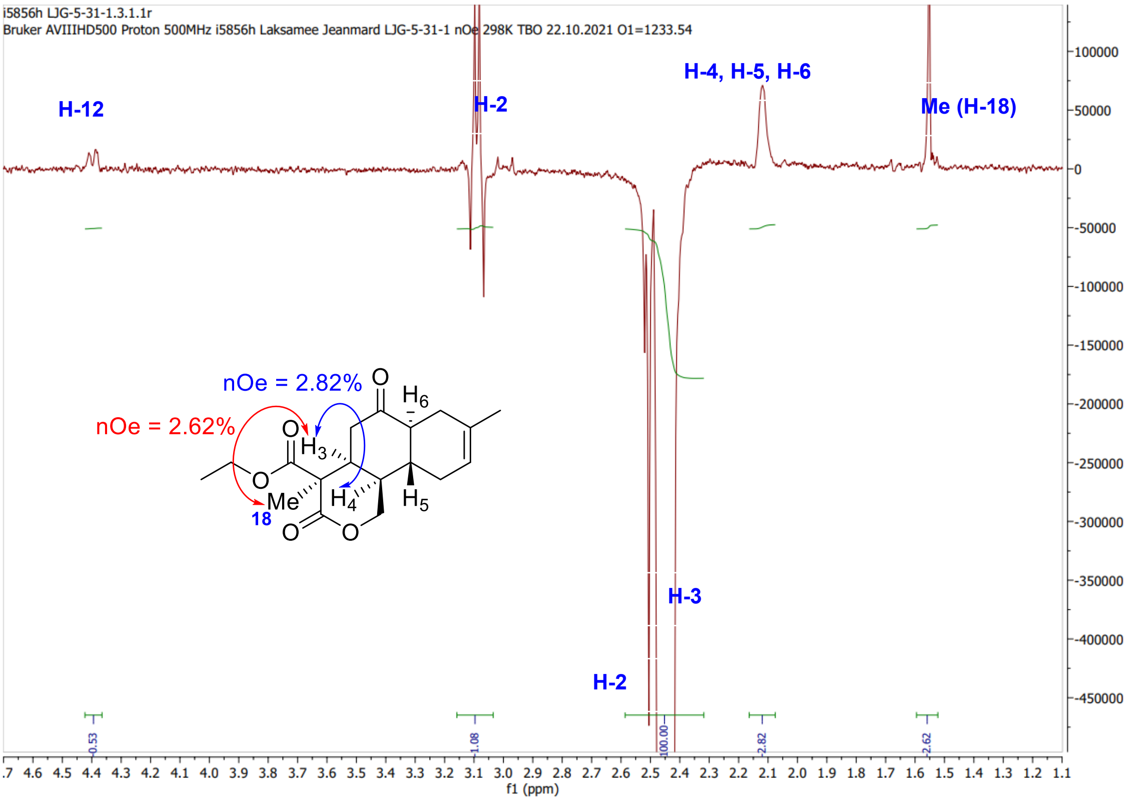

Figure S10: *The nOe analysis of H-3 in tricyclic lactone* ***25***

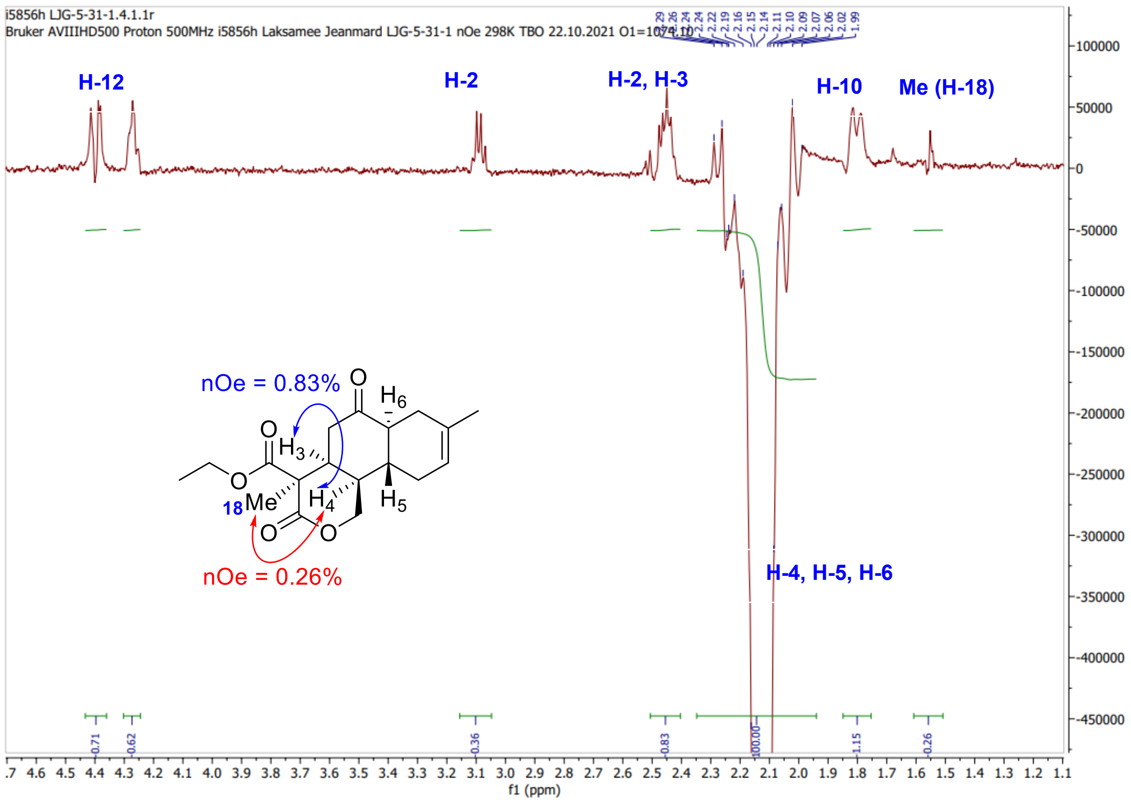

Figure S11: The nOe analysis of H-4 in tricyclic lactone ***25***

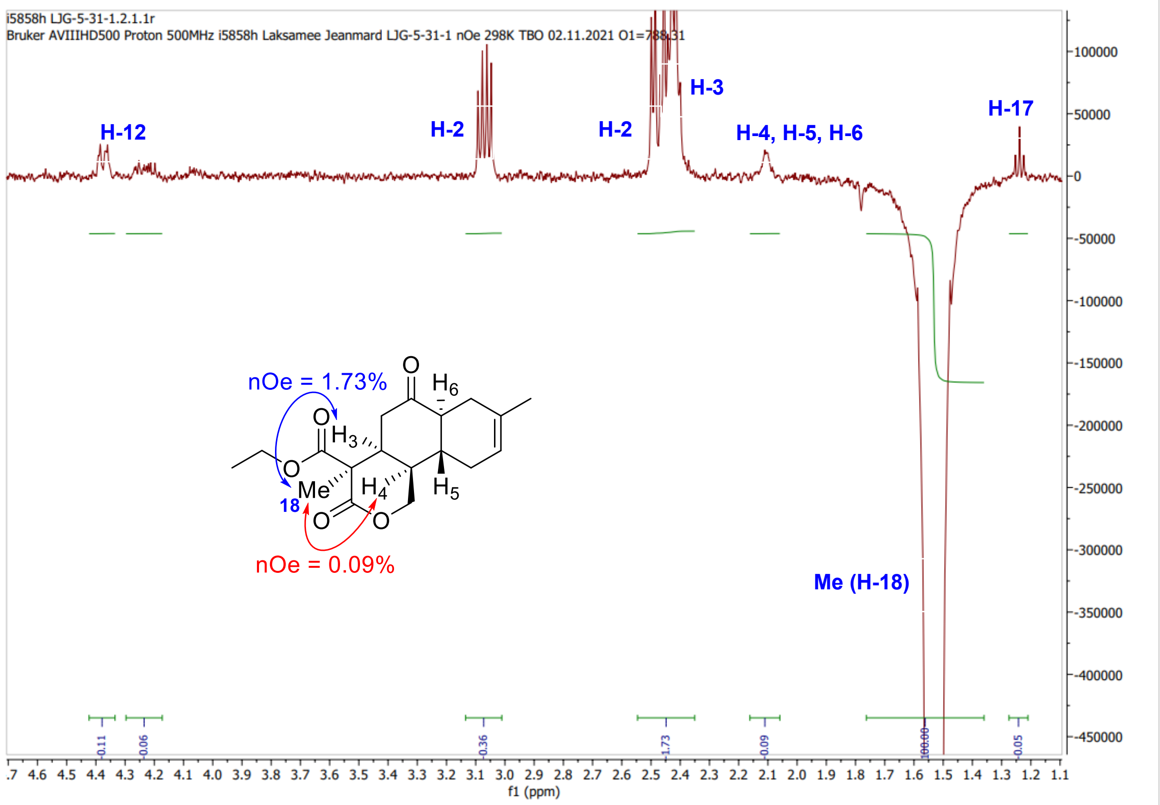

Figure S12: The nOe analysis of H-18 in tricyclic lactone ***25***

Ethyl (4*R*,4a*R*,6a*S*,10a*R*,10b*R*)-4,8-dimethyl-3-oxo-6-(((trifluoromethyl)sulfonyl)oxy)-3,4,4a,6a,7,10,10a,10b-octahydro-1*H*-benzo[*h*]isochromene-4-carboxylate (26)

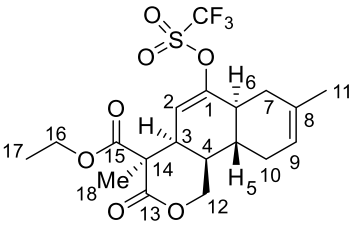

To a solution of freshly distilled diisopropylamine (0.2 mL, 1.43 mmol, 1.5 equiv) in THF (1.5 mL), was cooled to −78 °C and added *n*-BuLi (0.6 mL of a 2.5 M solution in hexane, 1.5 mmol, 1.5 equiv) dropwise. The stock solution of LDA was stirred at −78 °C for 45 minutes before adding dropwise into a solution of ketone **25** (32 mg, 0.099 mmol) in THF (0.29 mL). The mixture was stirred at −78 °C for 2 hours before adding a solution of *N*-(5-chloro-2-pyridyl)triflimide (61 mg, 0.15 mmol, 1.5 equiv) in THF (0.1 mL). The reaction mixture was stirred at −78 °C for 20 minutes and allowed to warm up to room temperature. The reaction mixture was quenched with H_2O (3 mL) and diluted with Et_2O (3 mL). The organic layer was separated, and the aqueous layer was extracted with Et_2O (3 x 5 mL). The combined organic layers were washed with brine, dried over anhydrous Na_2SO_4 and concentrated *in vacuo*. The crude product was purified by silica gel flash column chromatography (10–30% EtOAc/hexane) to afford the title compound **26** as a light yellow oil (28 mg, 62% yield): $\boldsymbol{R_f}$ = 0.29 (30% EtOAc/hexane); $[\alpha]_D^{20}$ = +42.6 (*c* 0.996, $CHCl_3$); **IR** (ATR) ν_{max} 2924, 2854, 1737 (C=O ester), 1418, 1214, 1143, 920 cm^{-1}; **^{1}H NMR** (400 MHz, $CDCl_3$) δ 5.89–5.81 (1H, dd, *J* = 4.3, 2.2 Hz, H-2), 5.42–5.32 (1H, m, H-9), 4.50 (1H, dd, *J* = 12.3, 7.1 Hz, H-12), 4.20 (2H, qd, *J* = 7.0, 1.2 Hz, H-16), 4.09 (1H, dd, *J* = 12.3, 9.2 Hz, H-12), 2.76–2.68 (1H, m, H-3), 2.49–2.37 (1H, m, H-6), 2.32–2.23 (2H, m, H-10), 2.23–2.15 (1H, m, H-4), 2.15–2.05 (1H, m, H-7), 2.02–1.87 (1H, m, H-7), 1.81–1.73 (1H, m, H-5), 1.71 (3H, s, H-11), 1.64 (3H, s, H-18), 1.33–1.15 (3H, m, H-17) ppm; **^{13}C NMR** (100 MHz, $CDCl_3$) δ 171.1 (C-13), 170.4 (C-15), 152.2 (C-1), 133.0 (C-8), 119.6 (C-9), 115.8 (C-2), 68.0 (C-12), 62.4 (C-16), 53.0 (C-14), 40.7 (C-3), 38.4 (C-6), 35.9 (C-5), 35.4 (C-4), 32.5 (C-10), 30.4 (C-7), 23.5 (C-11), 22.3 (C-18), 14.1 (C-17) ppm; **HRMS** (ESI) *m/z* cald for $C_{19}H_{23}F_3NaO_7S$ (M+Na^+) 475.1009, found 475.1010.

Ethyl (4*R*,4a*S*,6a*R*,10a*S*,10b*R*)-4,8-dimethyl-3-oxo-3,4,4a,6a,7,10,10a,10b-octahydro-1*H*-benzo[*h*]isochromene-4-carboxylate (27)

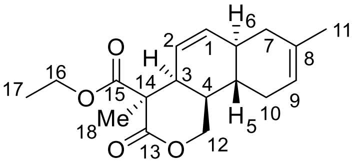

To a solution of vinyl triflate **26** (24 mg, 0.052 mmol) in DMF (3.2 mL) were added tributylamine (0.045 mL, 0.18 mmol, 3.5 equiv), $Pd(OAc)_2(PPh_3)_2$ (4 mg, 0.049 mmol, 0.095 equiv), followed by formic acid (0.006 mL, 0.13 mmol, 2.5 equiv). The reaction mixture was stirred at 50 °C for 2.5 hours. After this time, the reaction mixture was cooled to room temperature, quenched with H_2O (5 mL) and diluted with isopropyl ether (5 mL). The organic layer was separated, and the aqueous layer was extracted with isopropyl ether (3 × 5 mL). The combined organic layers were washed with brine, dried over anhydrous Na_2SO_4 and concentrated *in vacuo*. The crude product was purified by silica gel flash column chromatography (10–15% EtOAc/hexane) to give the title compound **27** as a yellow oil (12 mg, 75% yield): $\boldsymbol{R_f}$ = 0.23 (20% EtOAc/hexane); $\mathbf{[\alpha]_D^{20}}$ = +50.3 (*c* 0.885, $CHCl_3$); **IR** (ATR) ν_{max} 2913, 1737 (C=O ester), 1447, 1378, 1228, 1109, 1025 cm^{-1}; **^{1}H NMR** (400 MHz, $CDCl_3$) δ 5.84–5.72 (2H, m, H-1 + H-2), 5.39–5.29 (1H, m, H-9), 4.48 (1H, dd, *J* = 12.2, 7.2 Hz, H-12), 4.23–4.06 (3H, m, H-12 + H-16), 2.50–2.41 (1H, m, H-3), 2.19–2.12 (1H, m, H-4), 2.12–1.96 (3H, m, H-6 + H-10), 1.67 (3H, s, H-11), 1.63 (3H, s, H-18), 1.43–1.36 (1H, m, H-7), 1.36–1.31 (1H, m, H-5), 1.27–1.24 (1H, m, H-7), 1.24 (3H, t, *J* = 7.2 Hz, H-17) ppm; **^{13}C NMR** (100 MHz, $CDCl_3$) δ 172.5 (C-15), 171.0 (C-13), 134.2 (C-8), 133.6 (C-2), 123.1 (C-1), 119.9 (C-9), 68.7 (C-12), 61.9 (C-16), 53.4 (C-14), 41.2 (C-3), 36.8 (C-10), 36.7 (C-4), 36.1 (C-6), 34.6 (C-5), 30.1 (C-7), 23.5 (C-11), 22.4 (C-18), 14.1 (C-17) ppm; **HRMS** (ESI) *m/z* cald for $C_{18}H_{24}NaO_4$ (M+Na$^+$) 327.1567, found 327.1571.

Hydrolysis and decarboxylation of 27

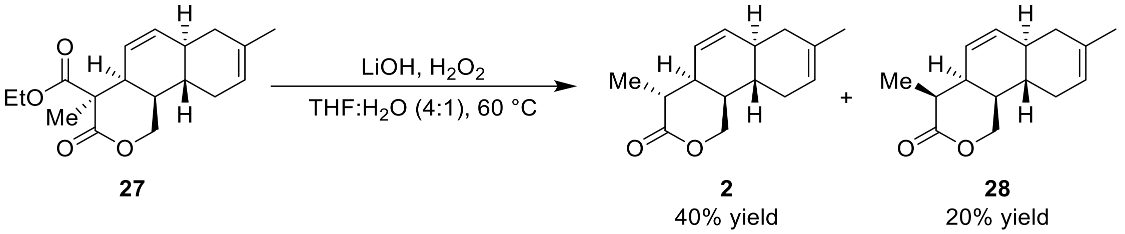

To a solution of ester **27** (15 mg, 0.048 mmol) in 0.5 mL of THF:H_2O (4:1), were added H_2O_2 (0.022 mL, 0.19 mmol, 4.0 equiv), followed by LiOH (2 mg, 0.09 mmol, 2.0 equiv). The reaction mixture was stirred at 60 °C for 5 hours before being added another portion of LiOH (2 mg, 0.09 mmol, 2.0 equiv). The reaction mixture was stirred for a further 1.5 hours and allowed to cool to room temperature. The mixture was diluted with Et_2O (5 mL) and washed with aqueous solution of 5% metasodium bisulfite (5 mL) and brine (5 mL). The organic layer was dried over anhydrous Na_2SO_4 and concentrated *in vacuo*. The crude product was purified by silica gel flash column chromatography (10–20% EtOAc/hexane) to deliver tricyclic lactone **2** (5 mg, 40% yield) and tricyclic lactone **28** (2 mg, 20% yield).

(4*R*,4a*S*,6a*R*,10a*S*,10b*R*)-4,8-Dimethyl-1,4,4a,6a,7,10,10a,10b-octahydro-3*H*-benzo[*h*]isochromen-3-one (2)

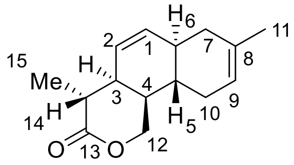

Data for 2 (a light yellow oil): ***R****f* = 0.40 (30% EtOAc/hexane); **[α]$_D^{20}$** = +81.2 (*c* 0.222, $CHCl_3$); **IR** (ATR) ν_{max} 2960, 2920, 1744 (C=O ester), 1440, 1261, 1176, 1103, 790 cm^{-1}; **^{1}H NMR** (400 MHz, $CDCl_3$) δ 5.72 (1H, ddd, *J* = 10.0, 2.1, 2.1 Hz, H-1), 5.57 (1H, ddd, *J* = 10.0, 3.1, 3.1 Hz, H-2), 5.41–5.34 (1H, m, H-9), 4.39 (1H, dd, *J* = 11.6, 3.4 Hz, H-12), 4.33 (1H, dd, *J* = 11.6, 3.0 Hz, H-12), 2.95 (1H, dddd, *J* = 10.1, 8.9, 3.1, 2.1 Hz, H-3), 2.84 (1H, dq, *J* = 10.1, 7.0 Hz, H-14), 2.35–2.27 (2H, m, H-7), 2.09–1.91 (5H, m, H-4 + H-5 + H-6 + H-10), 1.65 (3H, s, H-11), 1.23 (3H, d, *J* = 7.0 Hz, H-15) ppm; **^{13}C NMR** (100 MHz, $CDCl_3$) δ 176.1 (C-13), 134.8 (C-1), 133.9 (C-8), 124.1 (C-2), 120.1 (C-9), 67.3 (C-12), 38.7 (C-4), 37.3 (C-14), 37.1 (C-10), 35.5 (C-3), 33.3 (C-5), 30.5 (C-6), 30.2 (C-7), 23.5 (C-11), 12.6 (C-15) ppm; **HRMS** (ESI) *m/z* cald for $C_{15}H_{20}NaO_2$ (M+Na$^+$) 255.1356, found 255.1362.

(4*S*,4a*S*,6a*R*,10a*S*,10b*R*)-4,8-Dimethyl-1,4,4a,6a,7,10,10a,10b-octahydro-3*H*-benzo[*h*]isochromen-3-one (28)

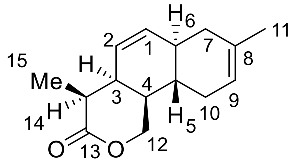

Data for 28 (a light yellow oil): R_f = 0.48 (30% EtOAc/hexane); $[\alpha]_D^{20}$ = +37.7 (*c* 0.079, $CHCl_3$); **IR** (ATR) ν_{max} 2923, 2849, 1751 (C=O ester), 1454, 1278, 1181, 1107, 787 cm^{-1}; **^{1}H NMR** (400 MHz, $CDCl_3$) δ 5.76–5.66 (2H, m, H-1 + H-2), 5.41–5.36 (1H, m, H-9), 4.45 (1H, dd, *J* = 11.8, 6.4 Hz, H-12), 4.07 (1H, dd, *J* = 11.8, 8.0 Hz, H-12), 2.42 (1H, dq, *J* = 11.6, 6.8 Hz, H-14), 2.27–2.19 (1H, m, H-3), 2.15–1.98 (4H, m, H-4, H-5, H-6, H-10), 1.81–1.48 (3H, m, H-7 + H-10), 1.67 (3H, s, H-11), 1.31 (3H, d, *J* = 6.8 Hz, H-15) ppm; **^{13}C NMR** (100 MHz, $CDCl_3$) δ 176.3 (C-13), 134.2 (C-8), 133.5 (C-1), 125.8 (C-2), 120.1 (C-9), 69.0 (C-12), 38.7 (C-14), 38.2 (C-3), 37.0, 36.9, 36.3, 35.1, 29.9, 23.5 (C-11), 14.0 (C-15) ppm; **HRMS** (ESI) *m/z* cald for $C_{15}H_{20}NaO_2$ (M+Na$^+$) 255.1356, found 255.1360.

nOe data for tricyclic lactone 2

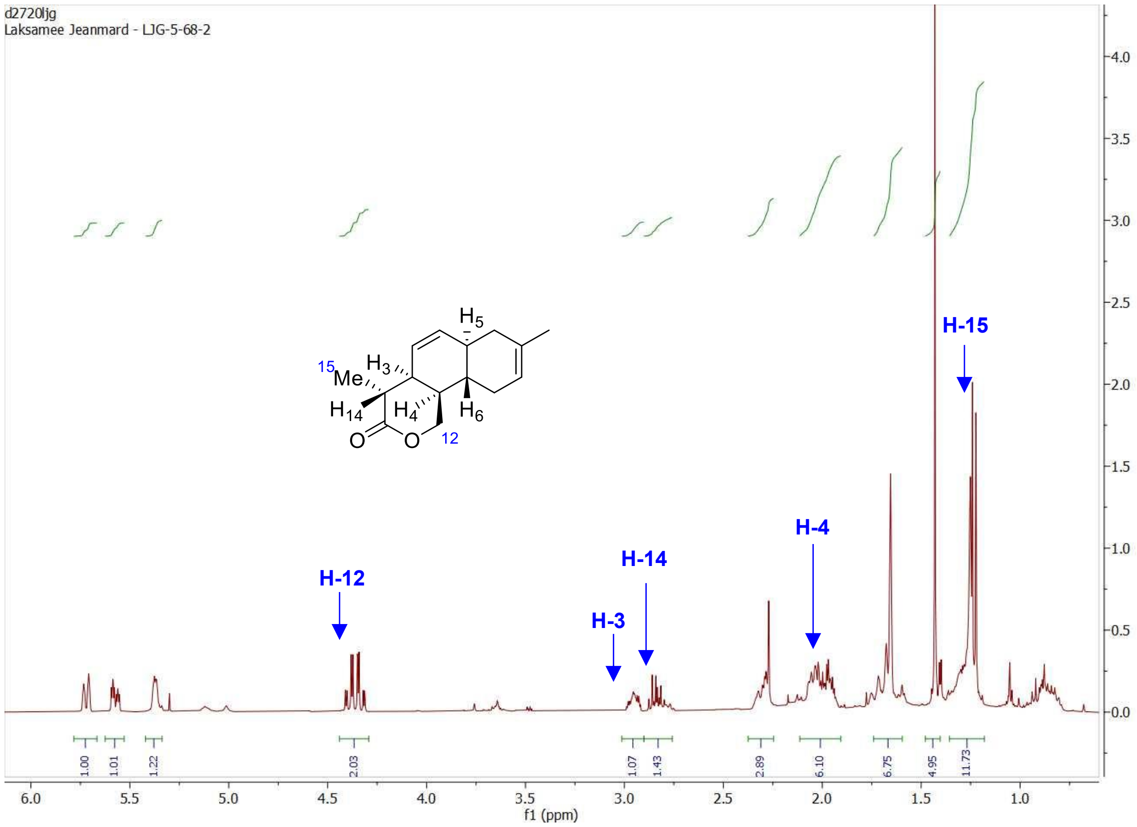

Figure S13: *The ^{1}H NMR spectrum of tricyclic lactone* ***2.***

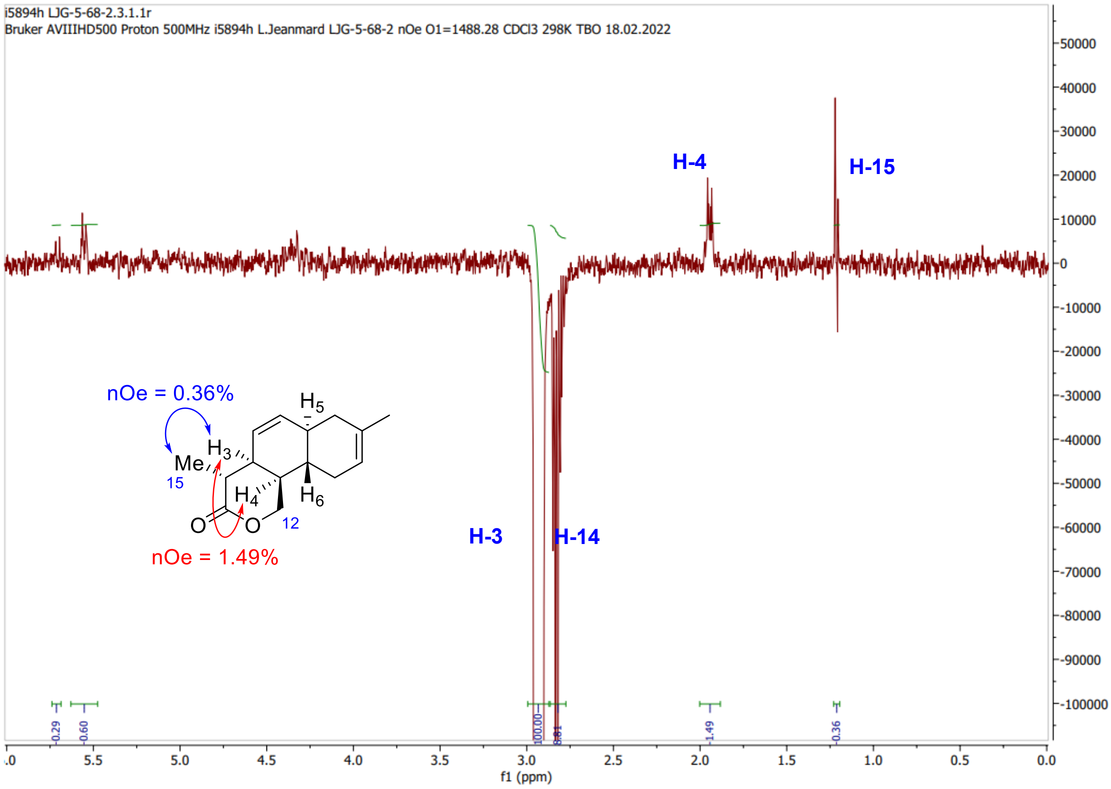

Figure S14: *The nOe analysis of H-3 in tricyclic lactone* ***2***

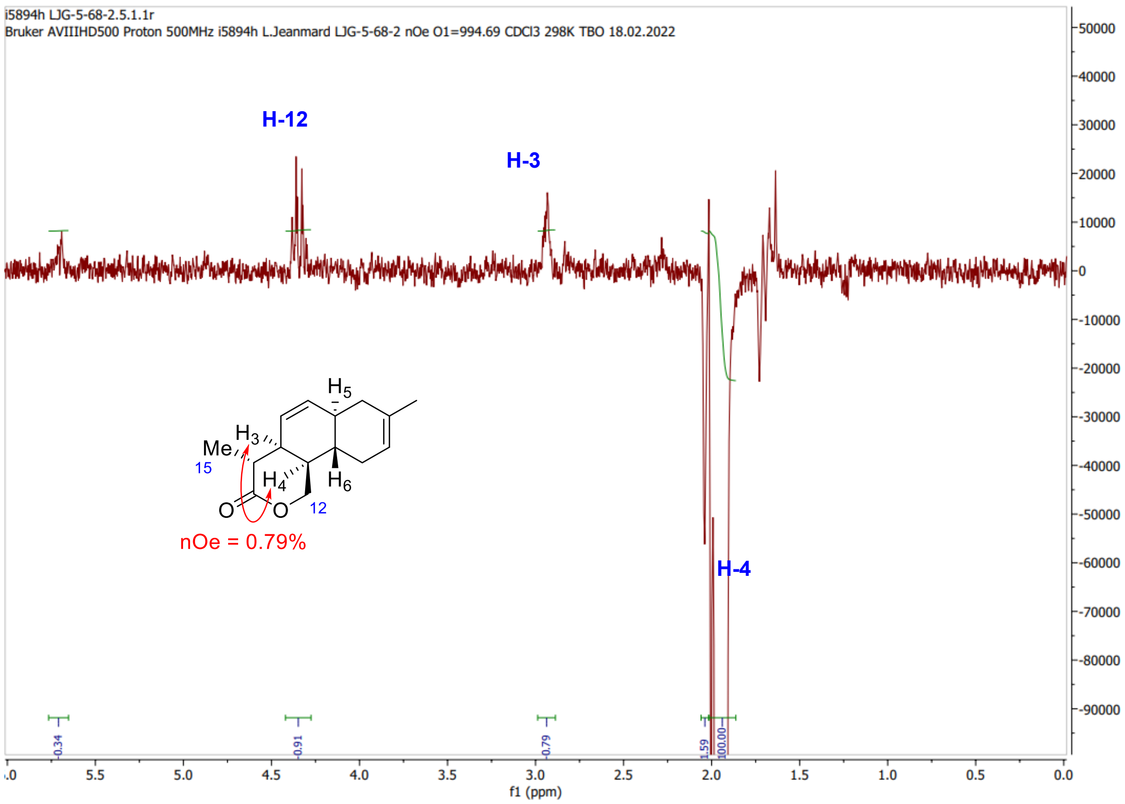

Figure S15: *The nOe analysis of H-4 in tricyclic lactone* ***2***

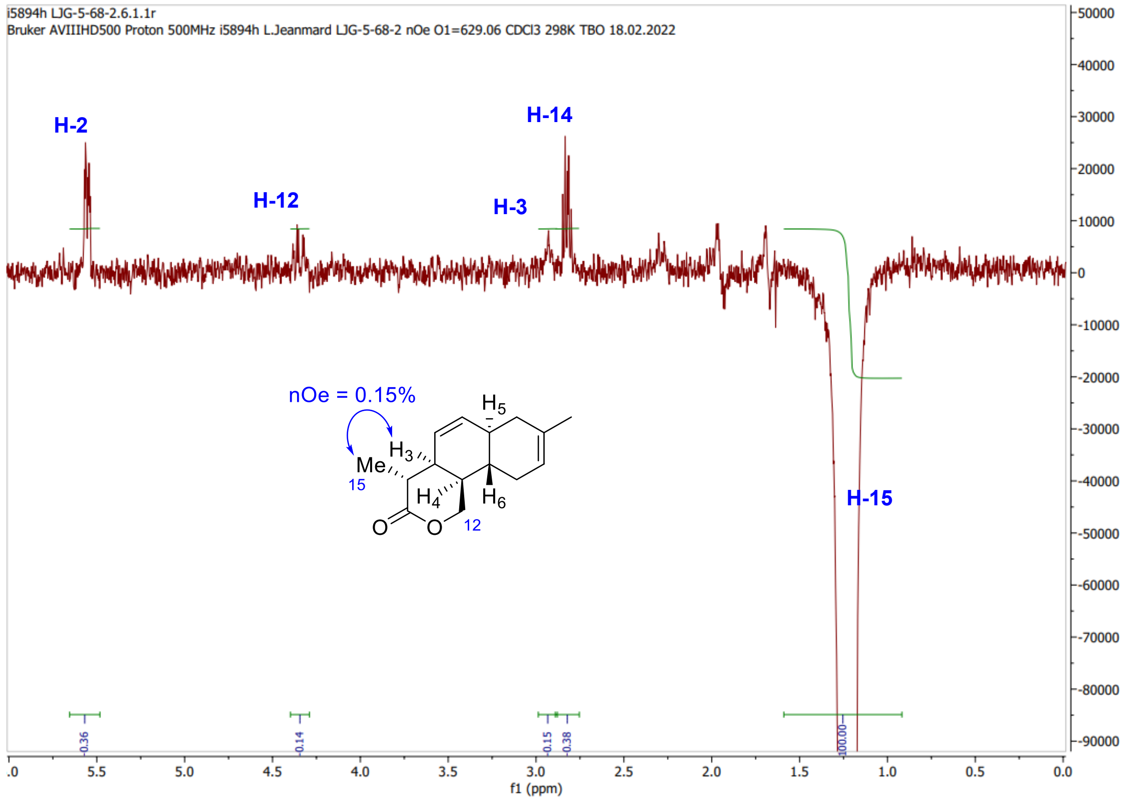

Figure S16: *The nOe analysis of H-15 in tricyclic lactone* ***2***

nOe data for tricyclic lactone 28

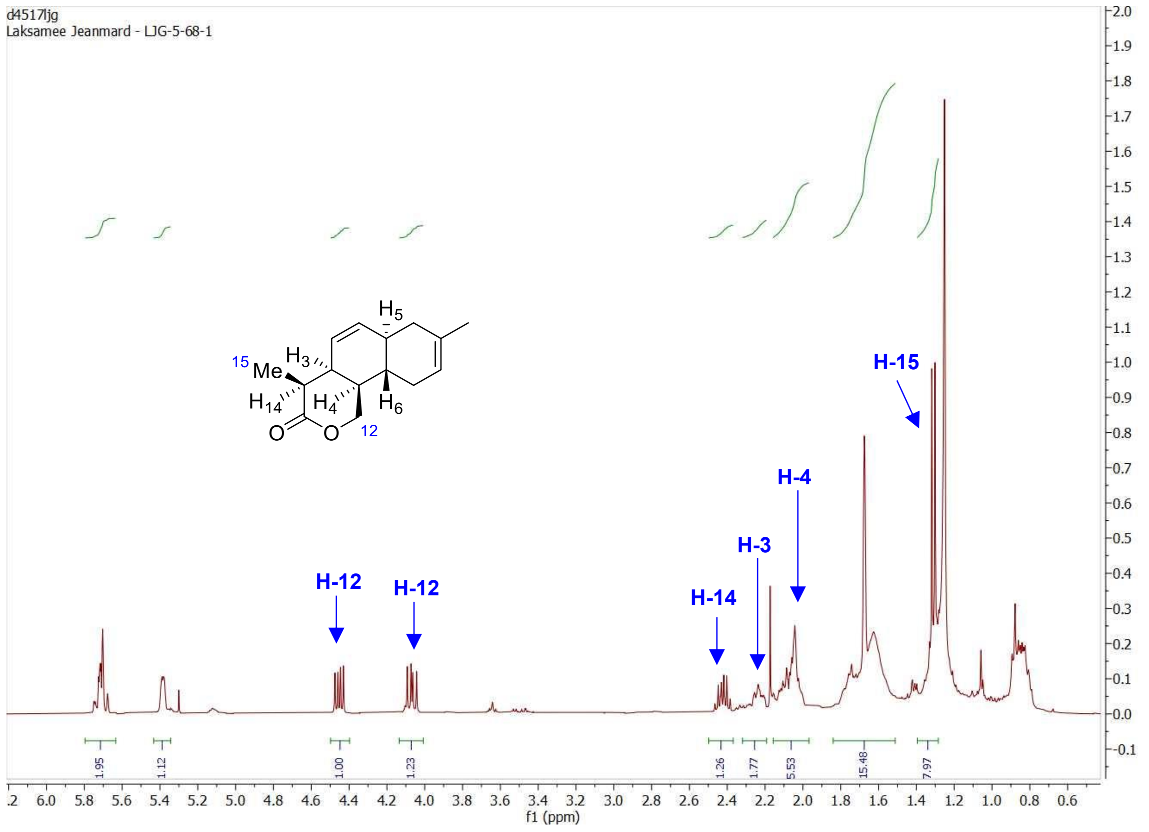

Figure S17: *The ^{1}H NMR spectrum of tricyclic lactone **28**.*

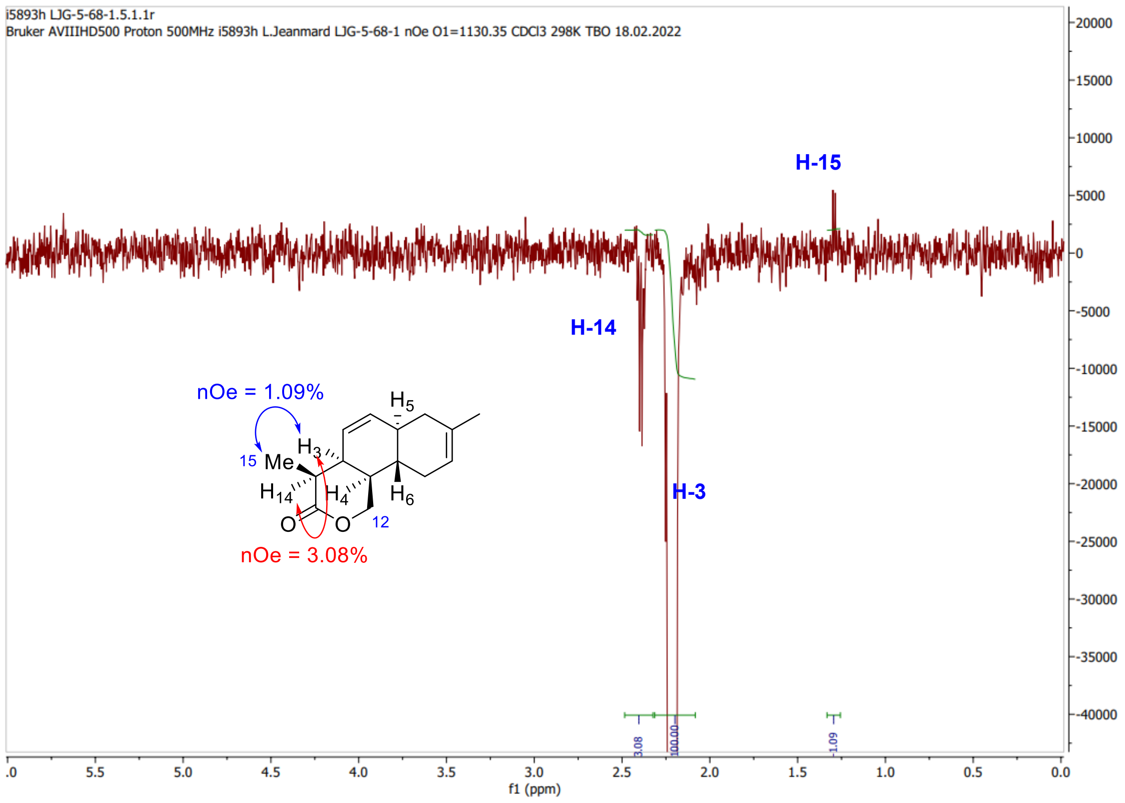

Figure S18: *The nOe analysis of H-3 in tricyclic lactone **28***

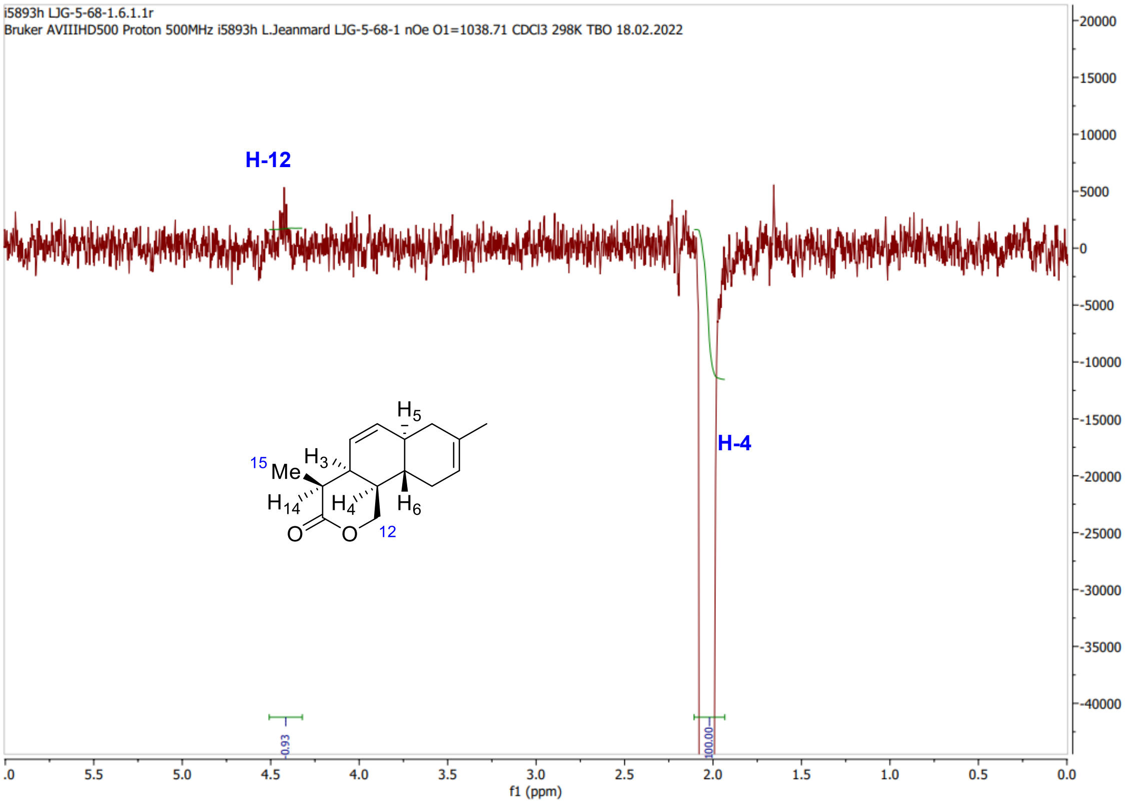

Figure S19: *The nOe analysis of H-4 in tricyclic lactone* ***28***

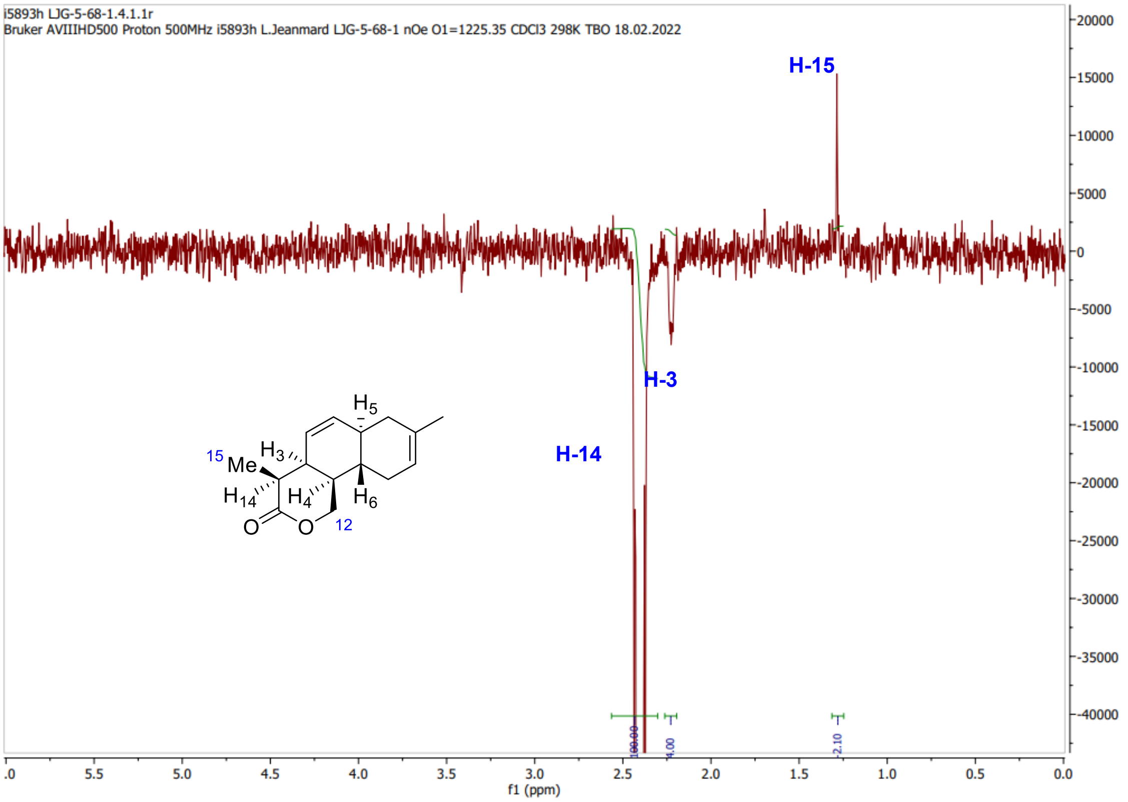

Figure S20: *The nOe analysis of H-14 in tricyclic lactone* ***28***

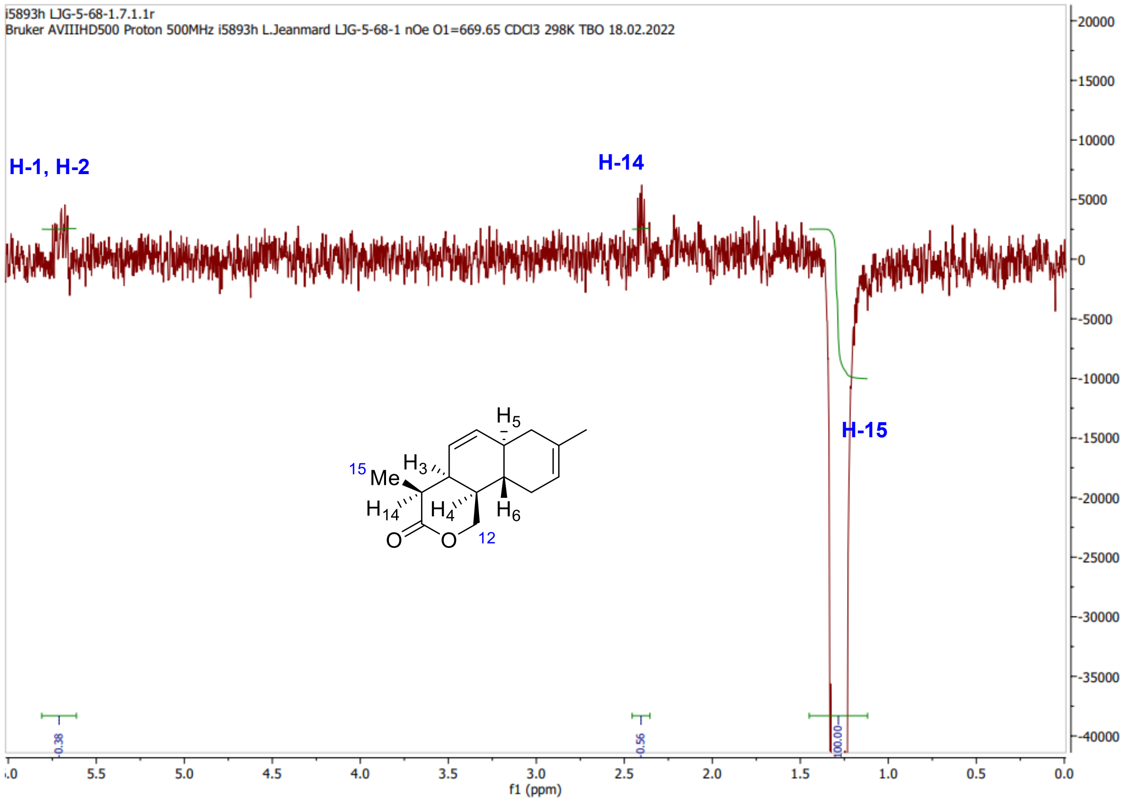

Figure S21: *The nOe analysis of H-15 in tricyclic lactone **28***

3) Additional optimisation tables

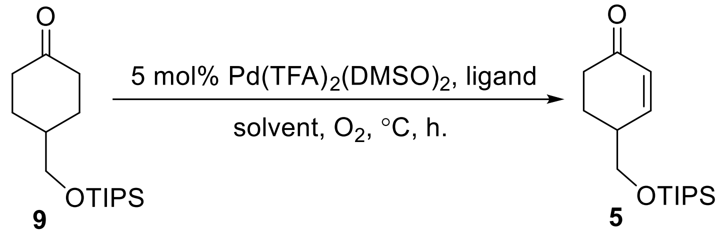

entry	Solvent	Temp (°C)	Time (h.)	Conversion (%)
1	AcOH	rt	96	5
2	AcOH	80	16	trace
3	AcOH	rt	16	trace
4	AcOH	80	16	trace
5	EtOAc	rt	16	trace
6	AcOH	80	16	trace
7	EtOAc	rt	16	trace

Table S1. Attempted desaturation of ketone **9**.

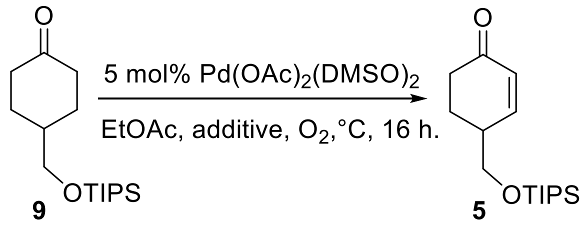

entry	Temp (°C)	Additive (2 eq.)	Conversion (%)
1	60	-	-
2	60	K_2CO_3	-
3	rt	K_2CO_3	-
4	60	NEt_3	-

Table S2. Additive screening to in the attempted desaturation of ketone **9**.

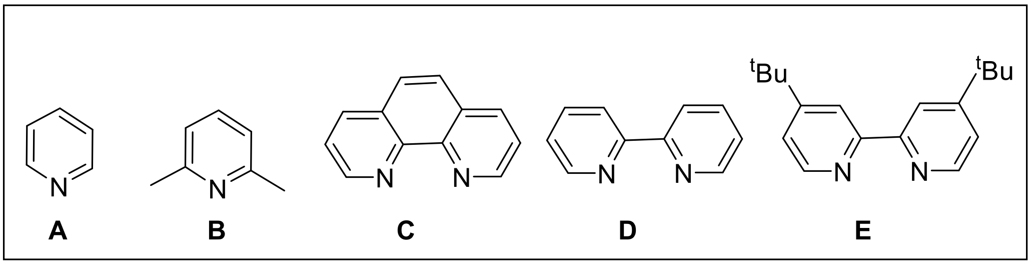

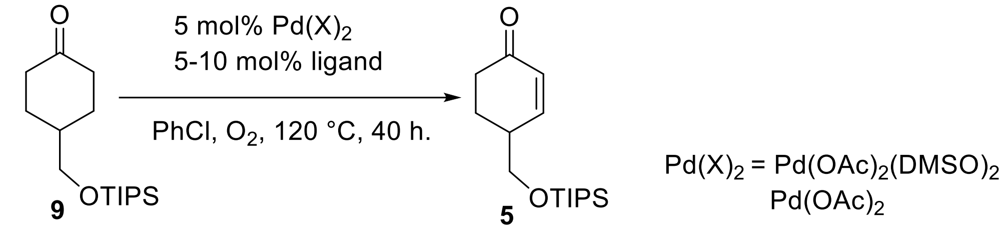

entry	Catalyst	Ligand (mol%)	Conversion (%)
**1**	$Pd(OAc)_2(DMSO)_2$	**A** (10)	19
**2**	$Pd(OAc)_2(DMSO)_2$	**B** (10)	-
**3**	$Pd(OAc)_2(DMSO)_2$	**C** (5)	-
**4**	$Pd(OAc)_2(DMSO)_2$	**D** (5)	17
**5**	$Pd(OAc)_2$	**A** (10)	18
**6**	$Pd(OAc)_2$	**B** (10)	-
**7**	$Pd(OAc)_2$	**C** (5)	-
**8**	$Pd(OAc)_2$	**D** (5)	58
**9**	$Pd(OAc)_2$	**E** (5)	76

Table S3. Pyridyl and bipyridyl ligand screening to improve the desaturation of ketone **9**.

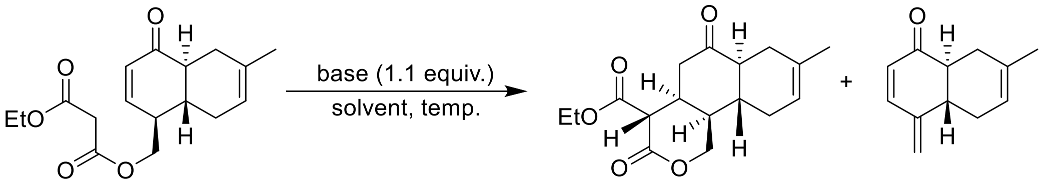

23 24 S1

Entry	Conditions	Temperature	Crude Ratio[a] **24** : **S1** : **23**	Isolated Yield **24**
1	NaH, DMF	0 °C to rt, 5 h	16.6 : 1 : 5.6	30%
2	KHMDS, THF	−78 °C to 0 °C, 8 h	1 : 2.6 : 0	–
3	DBU, THF	0 °C to rt, 23 h	only **S1**	–
4	Et_3N, THF	0 °C to rt to 40 °C, 23 h	n.r.	–

Table S4. *Screening conditions for the intramolecular Michael addition of malonate* ***23*** *[a] Crude ratio was determined by integration of peaks in the ^{1}H NMR spectrum of the reaction mixture.*

4) ^{1}H and ^{13}C NMR spectra

^{1}H NMR spectrum of **4-(((Triisopropylsilyl)oxy)methyl)cyclohexan-1-ol (8)** (400 MHz, $CDCl_3$)

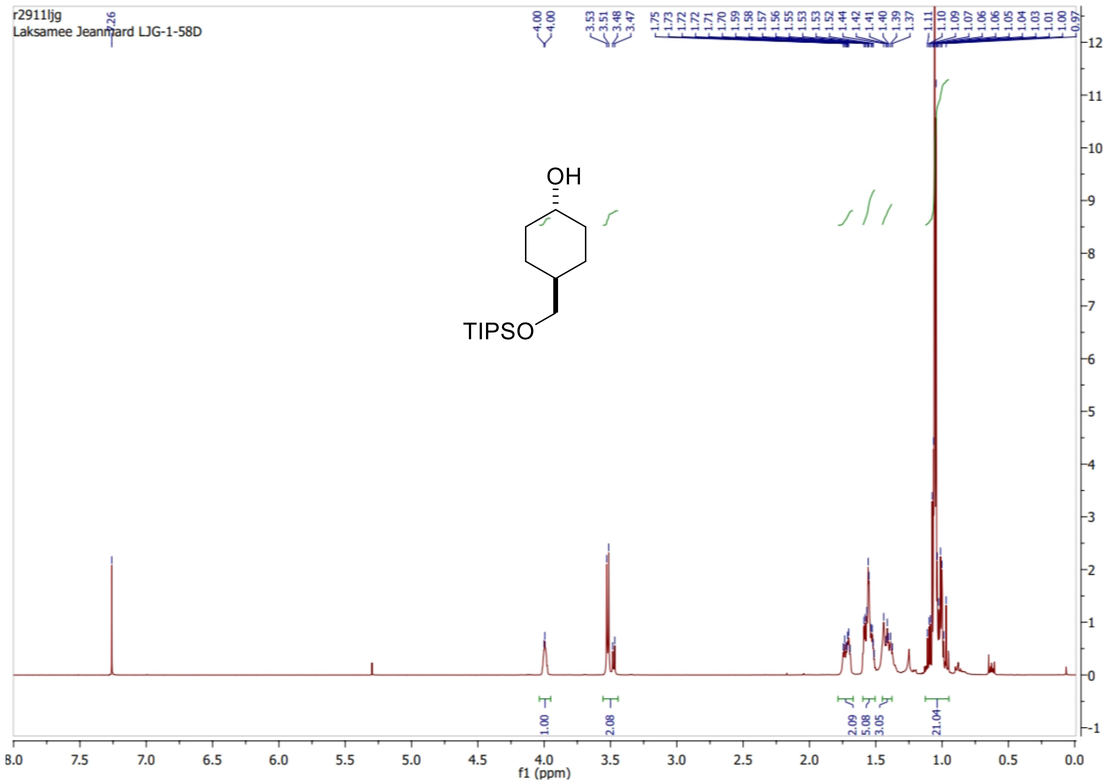

^{13}C NMR spectrum of **4-(((Triisopropylsilyl)oxy)methyl)cyclohexan-1-ol (8)** (100 MHz, $CDCl_3$)

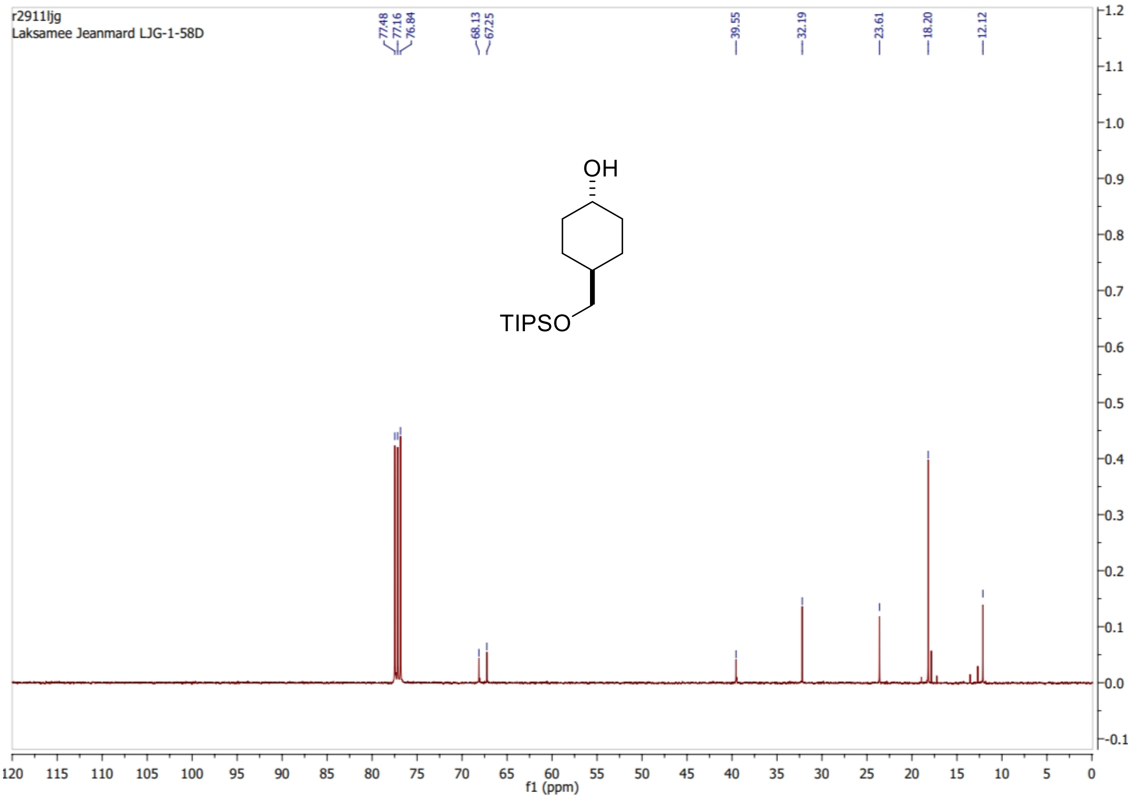

^{1}H NMR spectrum of **4-(((Triisopropylsilyl)oxy)methyl)cyclohexan-1-ol (8)** (400 MHz, $CDCl_3$)

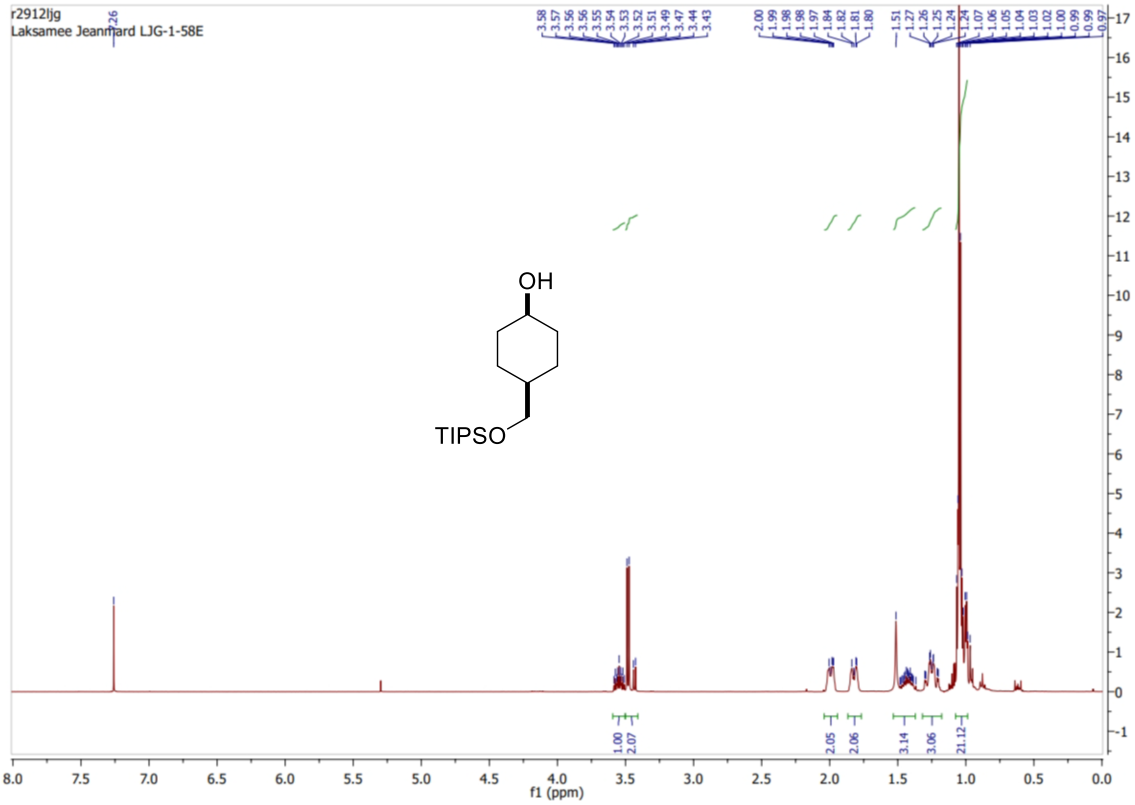

^{13}C NMR spectrum of **4-(((Triisopropylsilyl)oxy)methyl)cyclohexan-1-ol (8)** (100 MHz, $CDCl_3$)

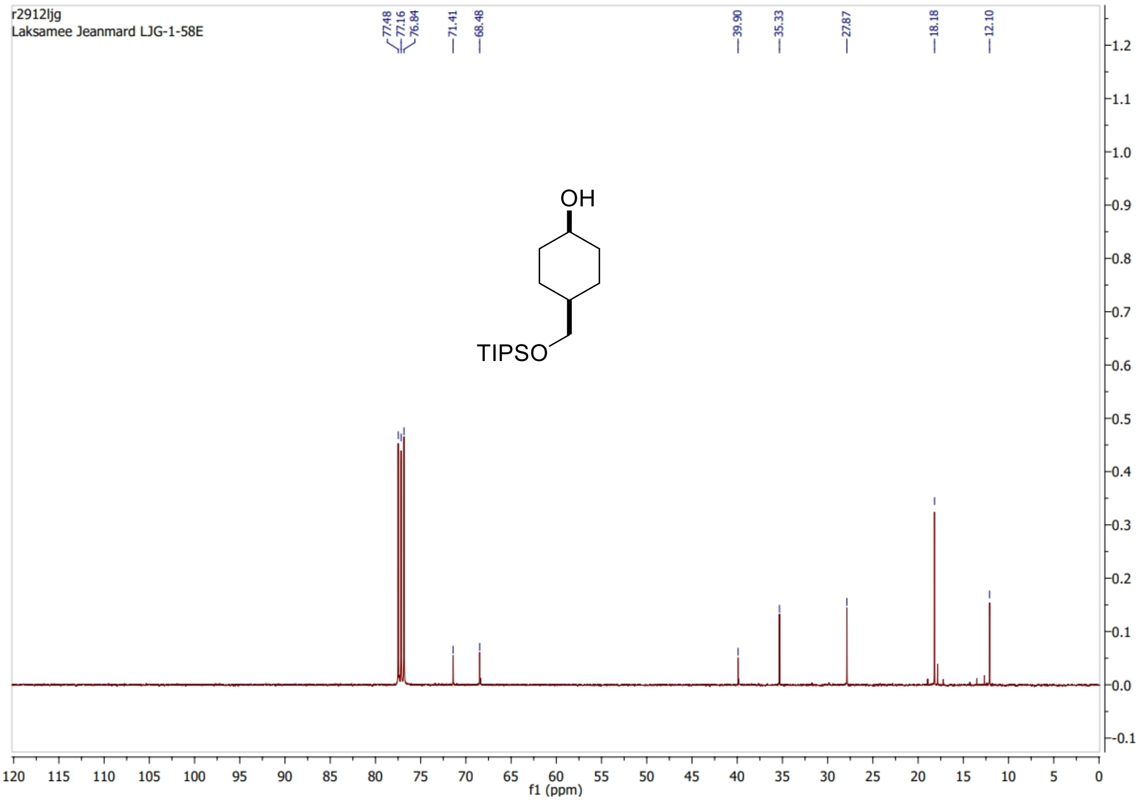

^{1}H NMR spectrum of **4-(((Triisopropylsilyl)oxy)methyl)cyclohexan-1-one (9)** (400 MHz, $CDCl_3$)

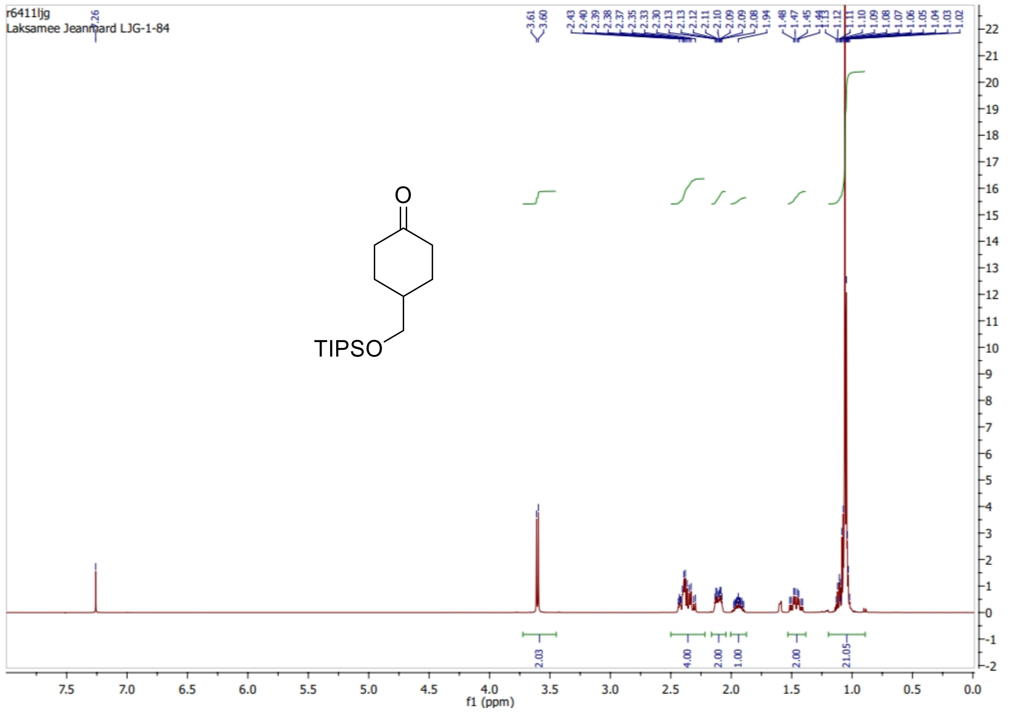

^{13}C NMR spectrum of **4-(((Triisopropylsilyl)oxy)methyl)cyclohexan-1-one (9)** (100 MHz, $CDCl_3$)

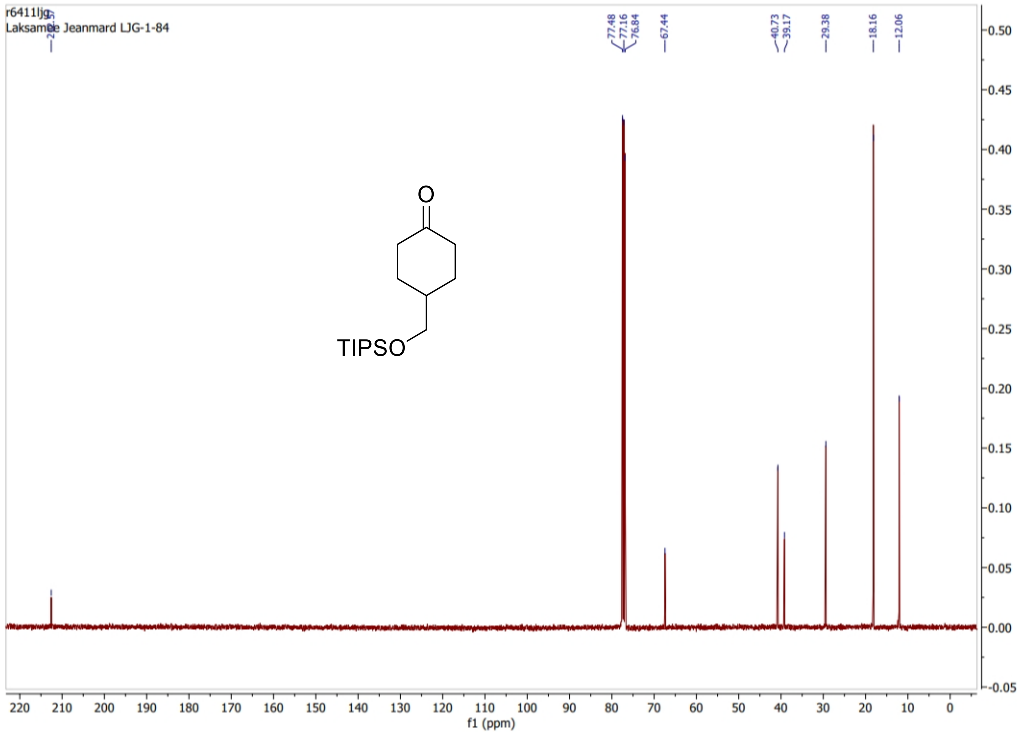

^{1}H NMR spectrum of **4-(Hydroxymethyl)cyclohex-2-en-1-one (14)** (400 MHz, $CDCl_3$)

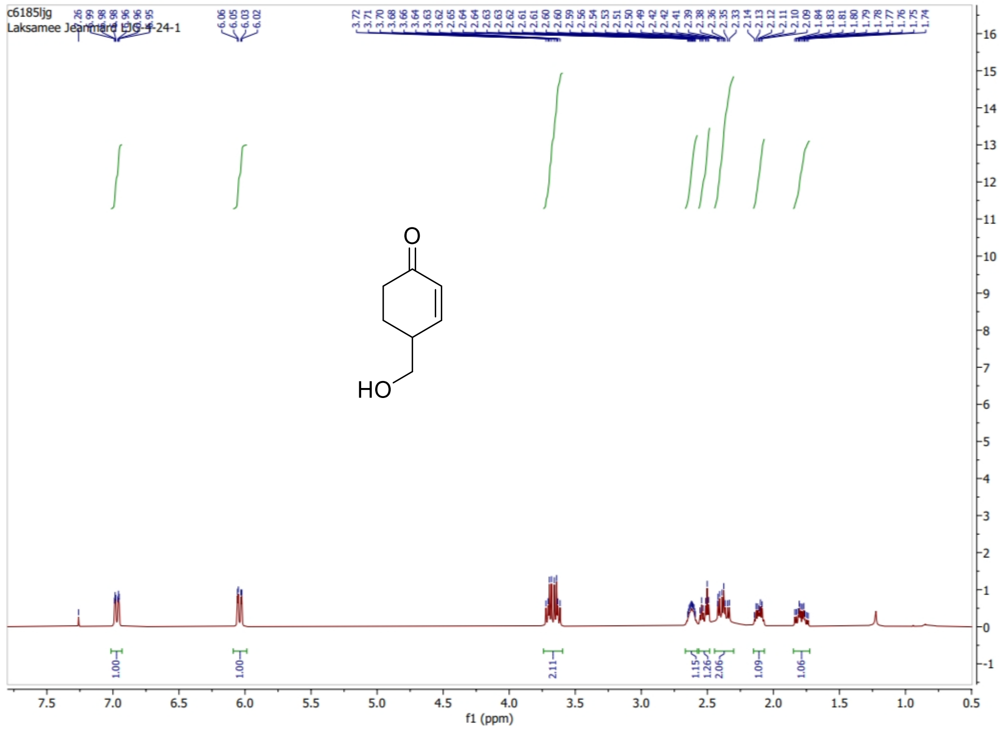

^{13}C NMR spectrum of **4-(Hydroxymethyl)cyclohex-2-en-1-one (14)** (100 MHz, $CDCl_3$)

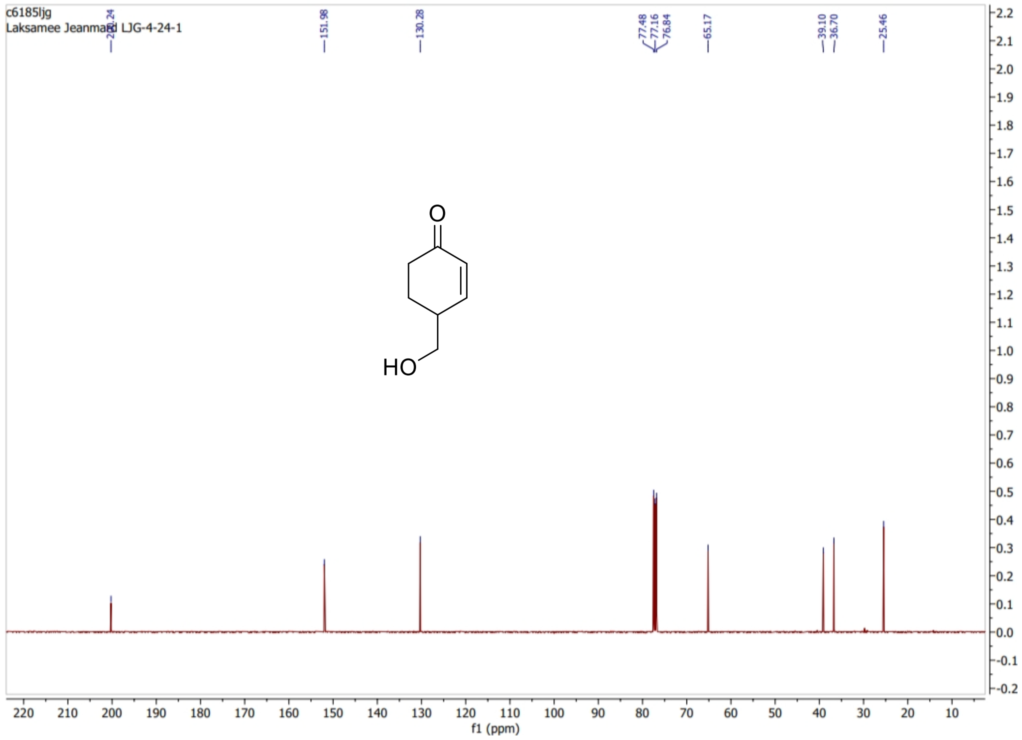

^{1}H NMR spectrum of **(*S*)-4-(((Triisopropylsilyl)oxy)methyl)cyclohex-2-en-1-one ((*S*)-5)** (400 MHz, $CDCl_3$)

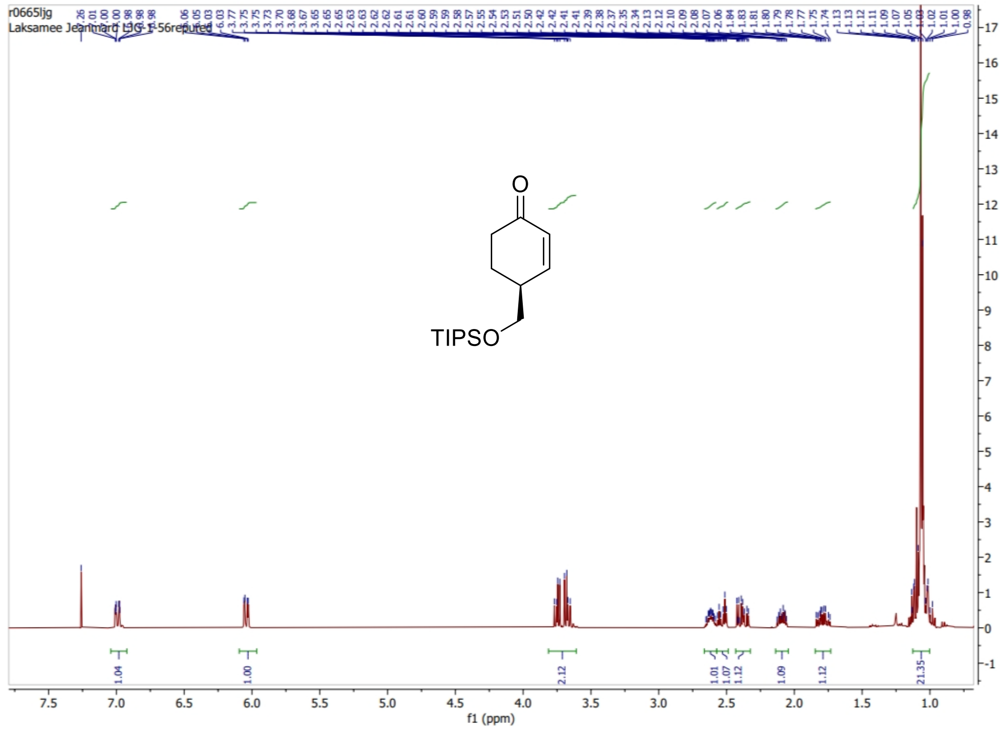

^{13}C NMR spectrum of **(*S*)-4-(((Triisopropylsilyl)oxy)methyl)cyclohex-2-en-1-one ((*S*)-5)** (100 MHz, $CDCl_3$)

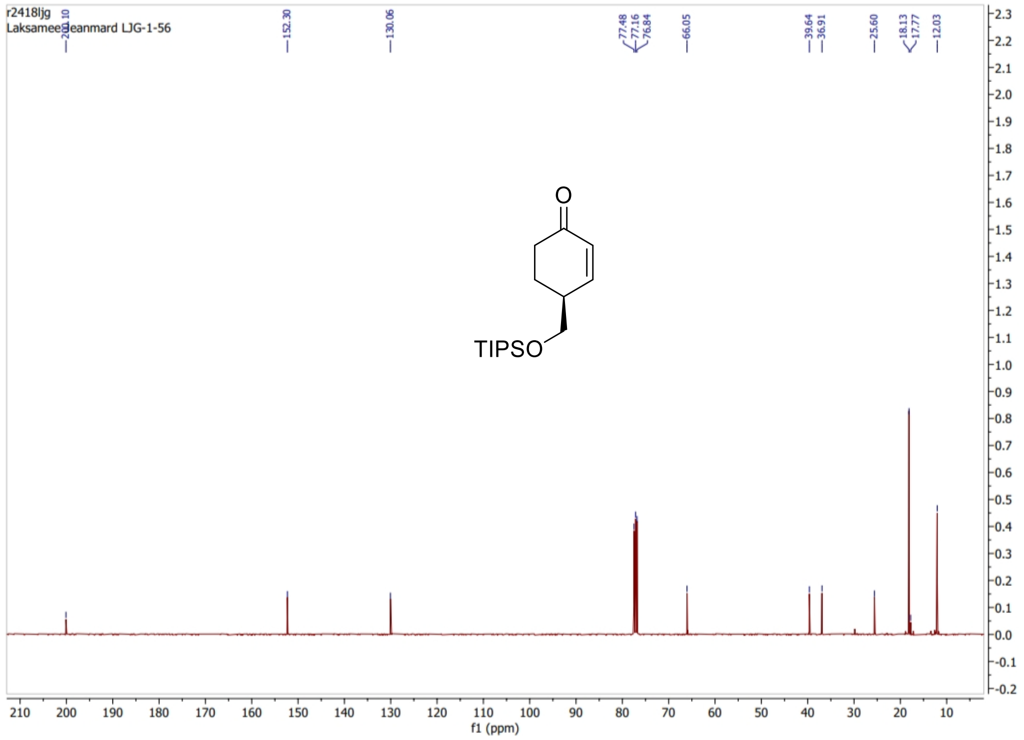

^{1}H NMR spectrum of **(4*S*,4a*R*,8a*R*)-7-Methyl-6-(phenylthio)-4-(((triisopropylsilyl)oxy) methyl)-3,4,4a,5,8,8a-hexahydronaphthalen-1(2*H*)-one (4)** (400 MHz, $CDCl_3$)

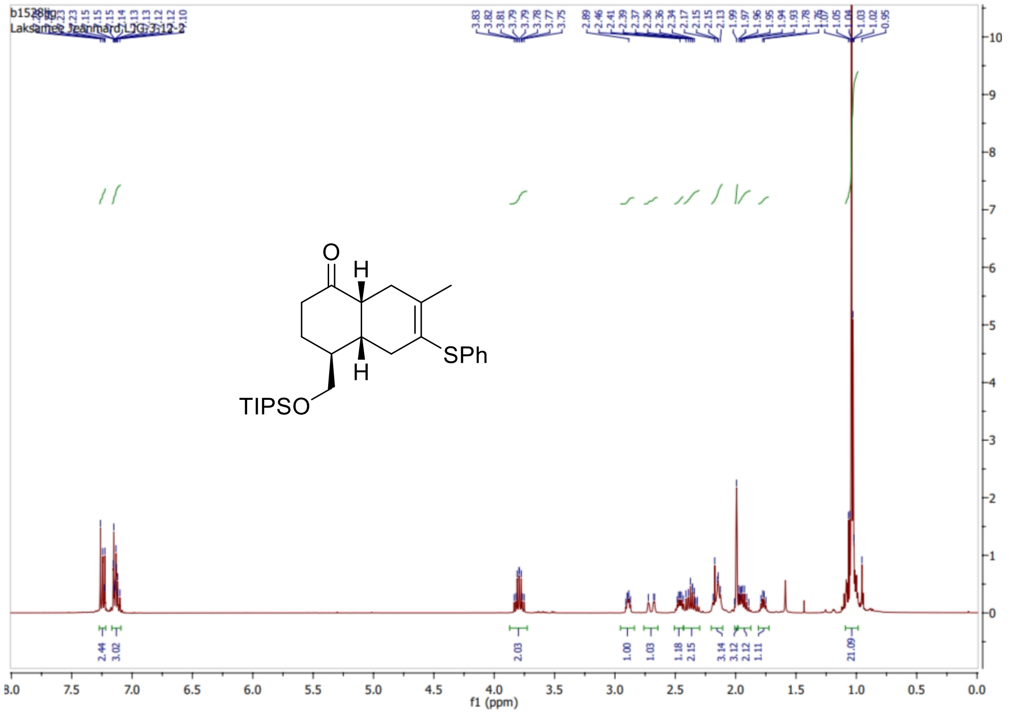

^{13}C NMR spectrum of **(4*S*,4a*R*,8a*R*)-7-Methyl-6-(phenylthio)-4-(((triisopropylsilyl)oxy) methyl)-3,4,4a,5,8,8a-hexahydronaphthalen-1(2*H*)-one (4)** (100 MHz, $CDCl_3$)

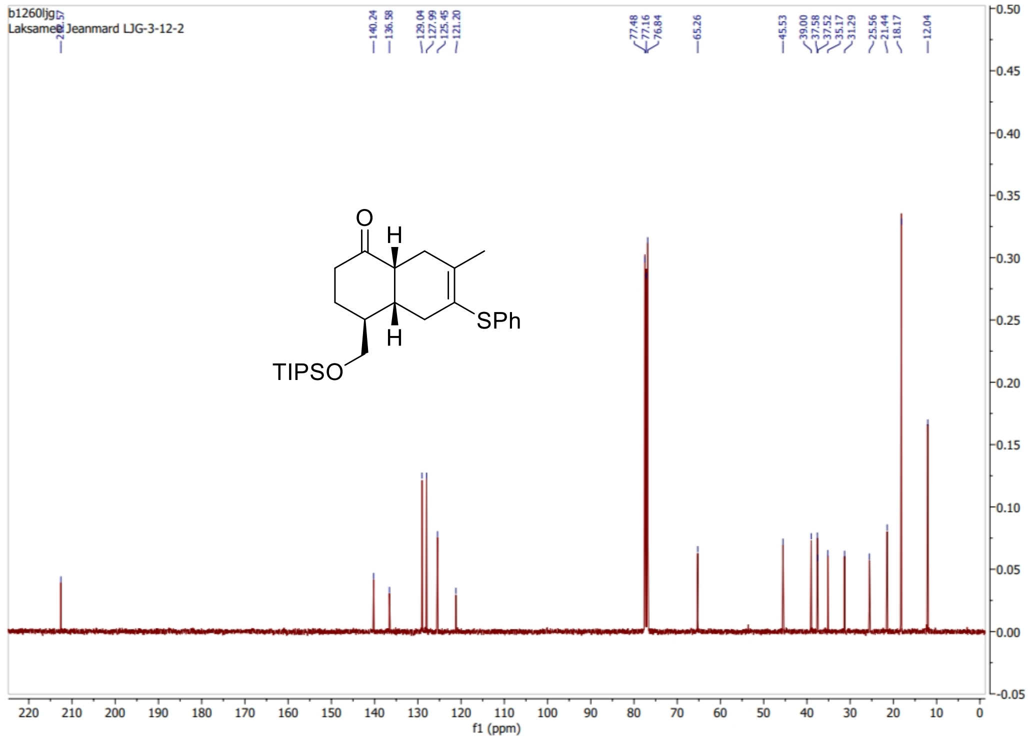

^{1}H NMR spectrum of **(4*S*,4a*R*,8a*S*)-7-Methyl-6-(phenylthio)-4-(((triisopropylsilyl)oxy) methyl)-3,4,4a,5,8,8a-hexahydronaphthalen-1(2*H*)-one (16)** (400 MHz, $CDCl_3$)

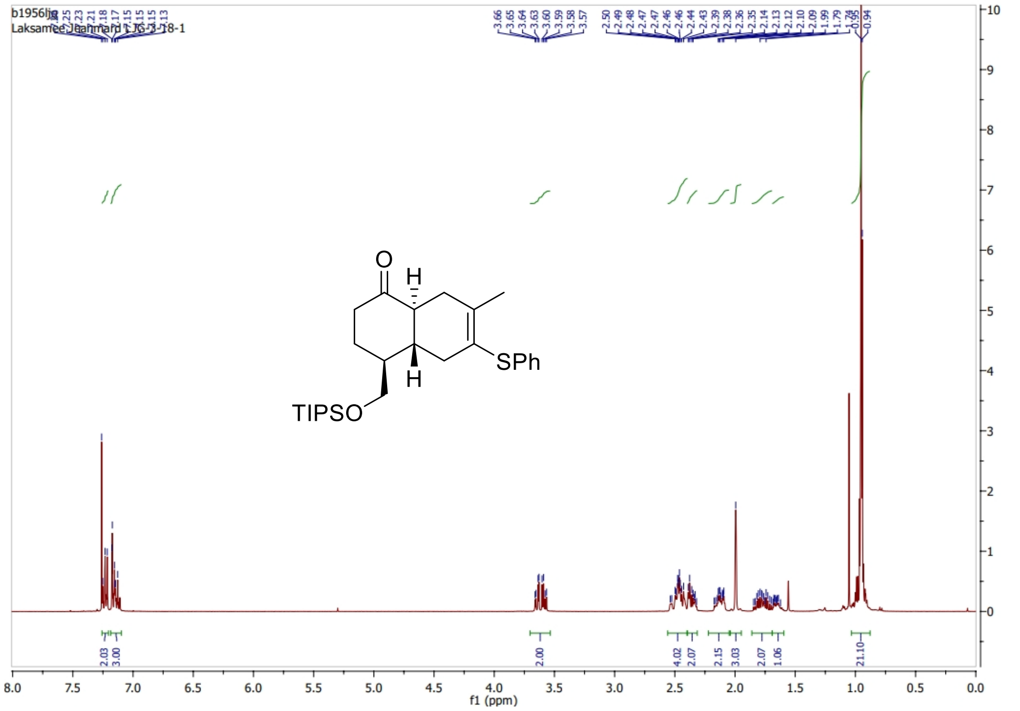

^{13}C NMR spectrum of **(4*S*,4a*R*,8a*S*)-7-Methyl-6-(phenylthio)-4-(((triisopropylsilyl)oxy) methyl)-3,4,4a,5,8,8a-hexahydronaphthalen-1(2*H*)-one (16)** (100 MHz, $CDCl_3$)

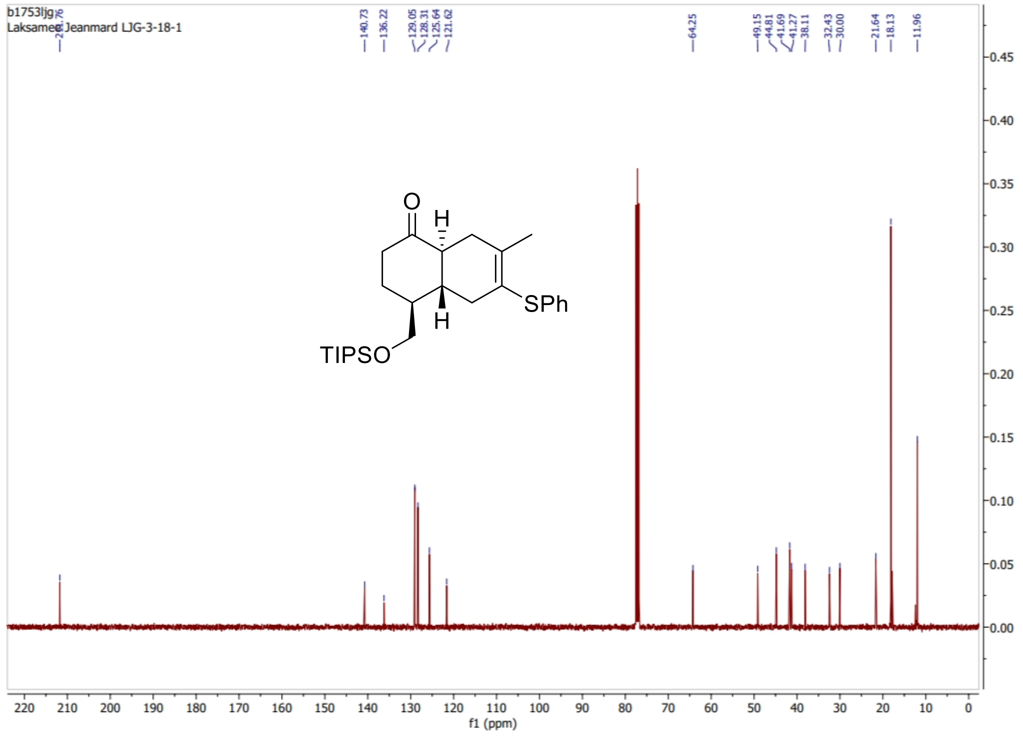

^{1}H NMR spectrum of **(4*S*,4a*R*,8a*S*)-7-Methyl-4-(((triisopropylsilyl)oxy)methyl)-3,4,4a,5,8,8a-hexahydronaphthalen-1(2*H*)-one (17)** (400 MHz, $CDCl_3$)

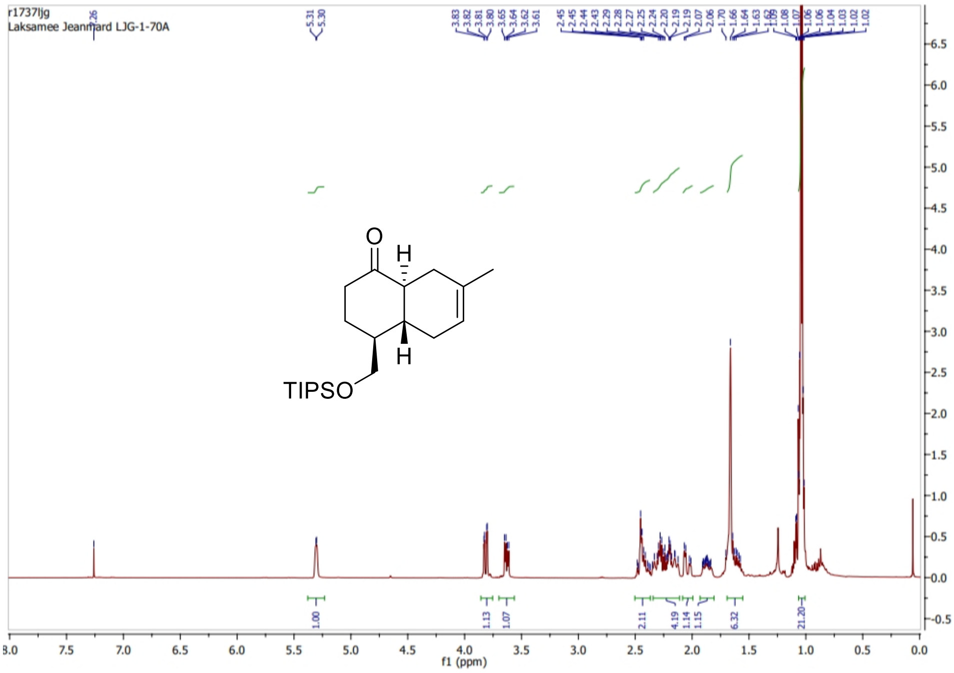

^{13}C NMR spectrum of **(4*S*,4a*R*,8a*S*)-7-Methyl-4-(((triisopropylsilyl)oxy)methyl)-3,4,4a,5,8,8a-hexahydronaphthalen-1(2*H*)-one (17)** (100 MHz, $CDCl_3$)

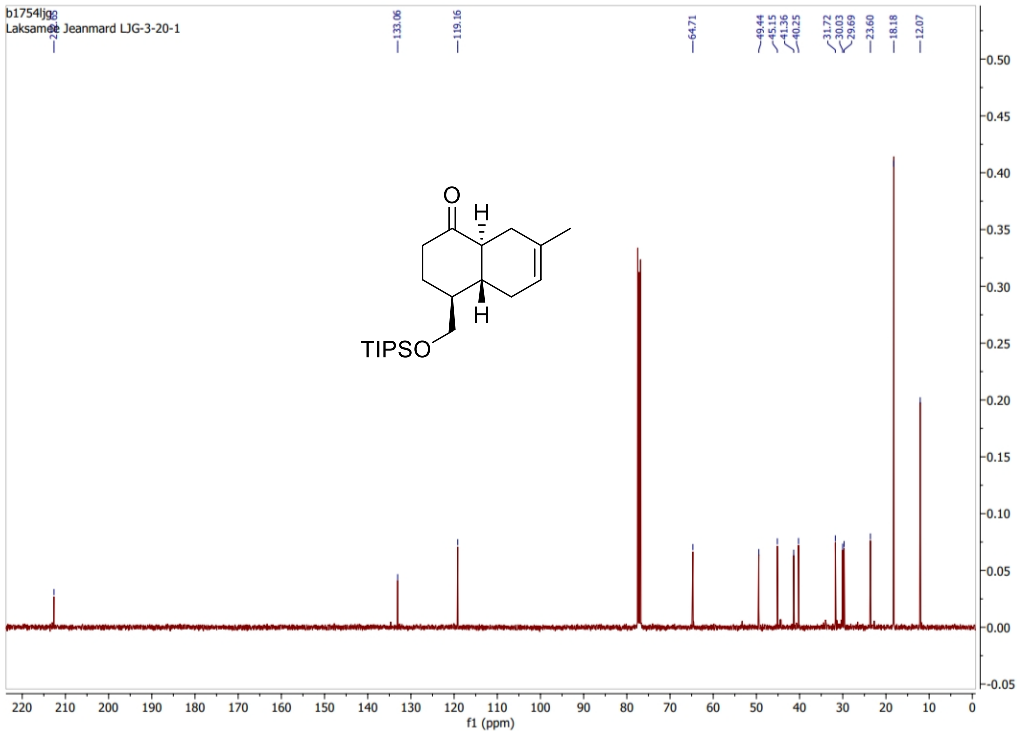

^{1}H NMR spectrum of **Triethyl(((4*S*,4a*R*,8a*S*)-7-methyl-4-(((triisopropylsilyl)oxy)methyl)-3,4,4a,5,8,8a-hexahydronaphthalen-1-yl)oxy)silane (20)** (400 MHz, $CDCl_3$)

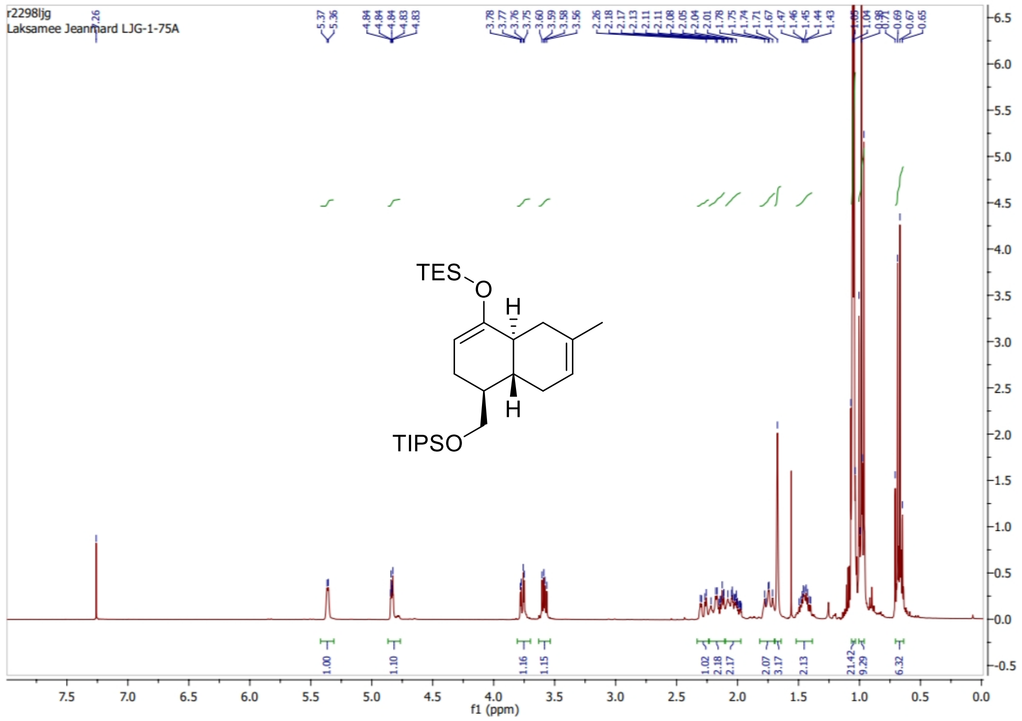

^{13}C NMR spectrum of **Triethyl(((4*S*,4a*R*,8a*S*)-7-methyl-4-(((triisopropylsilyl)oxy)methyl)-3,4,4a,5,8,8a-hexahydronaphthalen-1-yl)oxy)silane (20)** (100 MHz, $CDCl_3$)

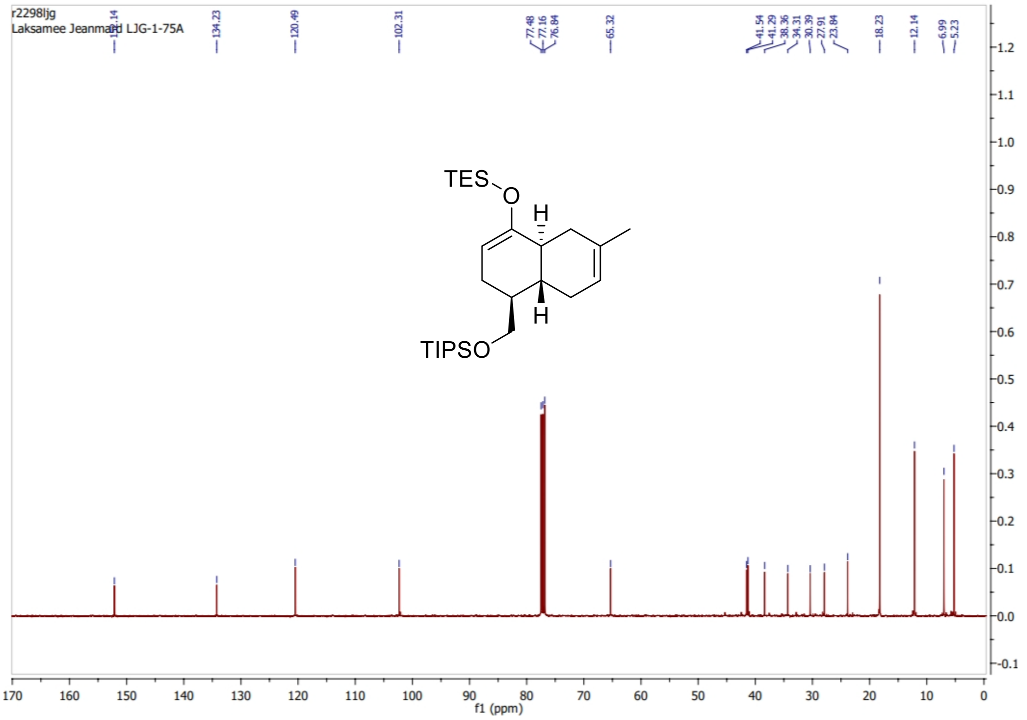

^{1}H NMR spectrum of **(4*S*,4a*R*,8a*S*)-7-Methyl-4-(((triisopropylsilyl)oxy)methyl)-4a,5,8,8a-tetrahydronaphthalen-1(4*H*)-one (21)** (400 MHz, $CDCl_3$)

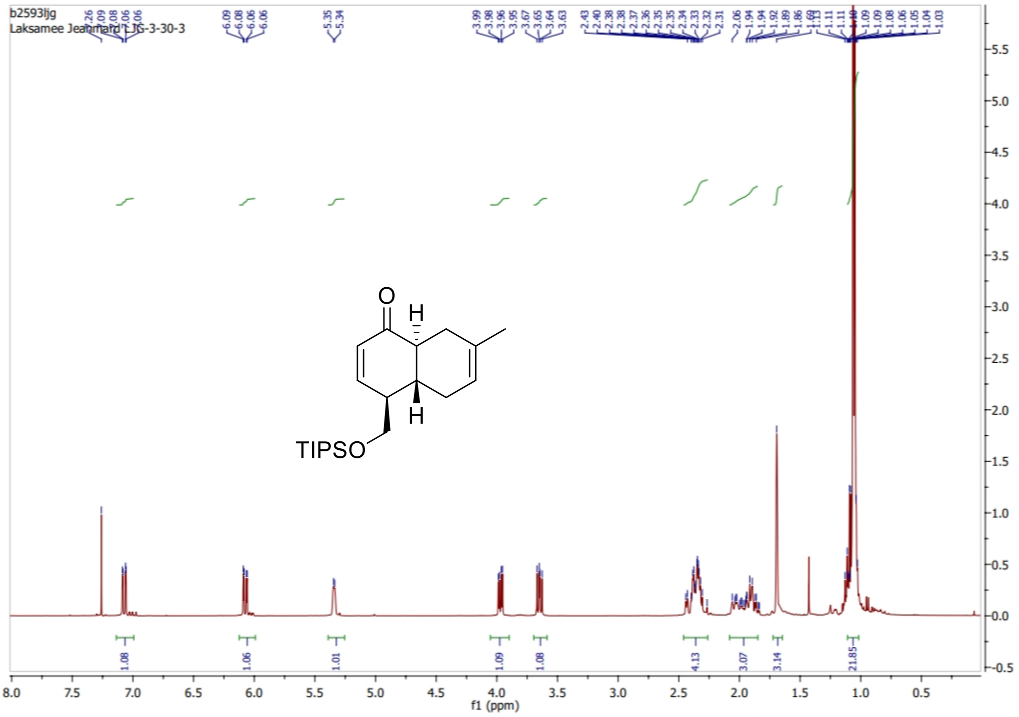

^{13}C NMR spectrum of **(4*S*,4a*R*,8a*S*)-7-Methyl-4-(((triisopropylsilyl)oxy)methyl)-4a,5,8,8a-tetrahydronaphthalen-1(4*H*)-one (21)** (100 MHz, $CDCl_3$)

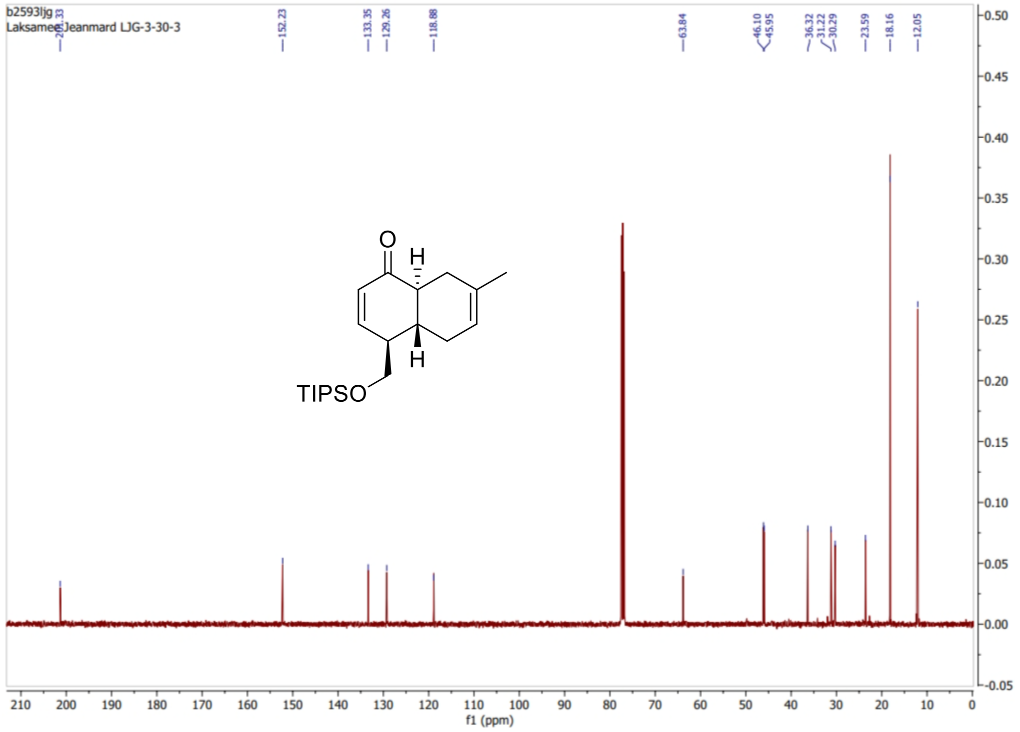

^{1}H NMR spectrum of **(4*S*,4a*R*,8a*S*)-4-(Hydroxymethyl)-7-methyl-4a,5,8,8a-tetrahydronaphthalen-1(4*H*)-one (22)** (400 MHz, $CDCl_3$)

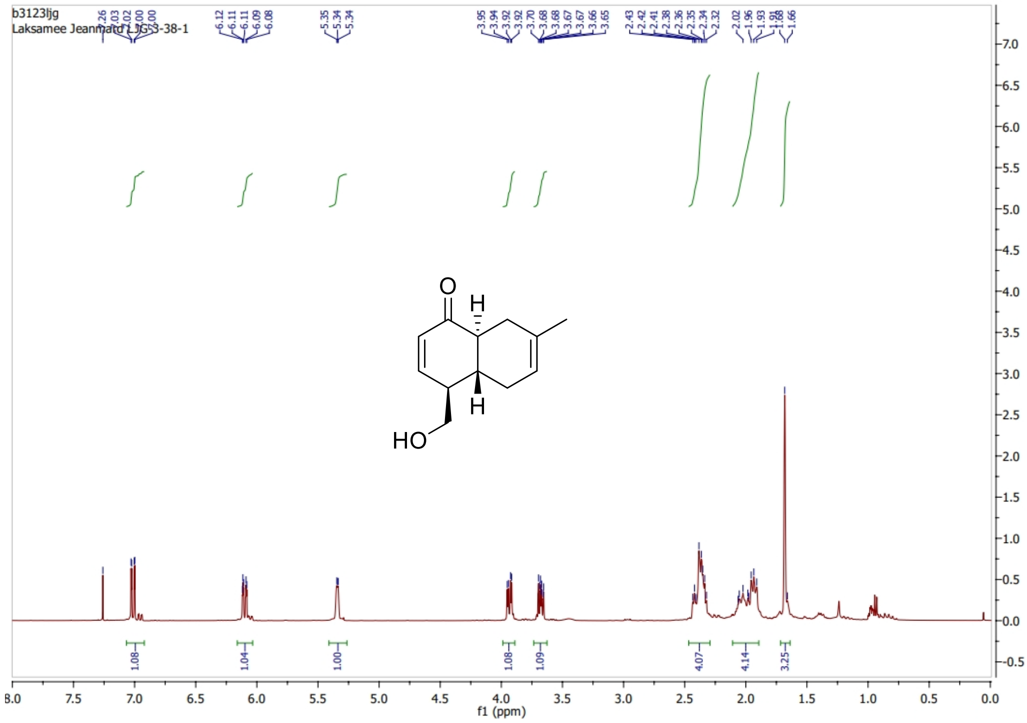

^{13}C NMR spectrum of **(4*S*,4a*R*,8a*S*)-4-(Hydroxymethyl)-7-methyl-4a,5,8,8a-tetrahydronaphthalen-1(4*H*)-one (22)** (100 MHz, $CDCl_3$)

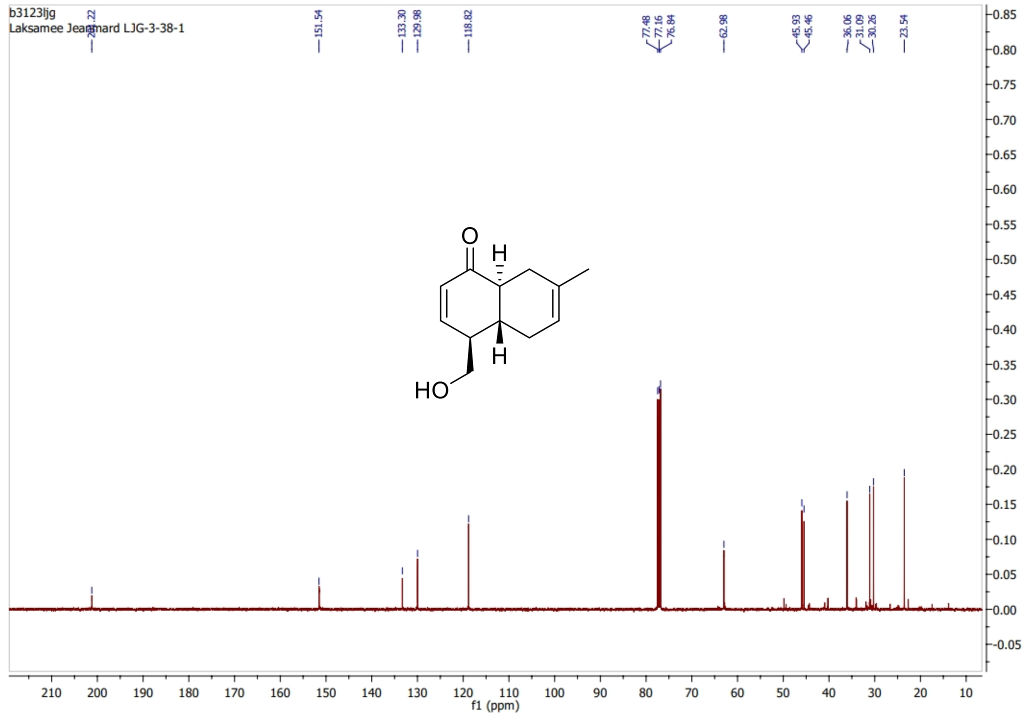

^{1}H NMR spectrum of **Ethyl (((1*S*,4a*S*,8a*R*)-6-methyl-4-oxo-1,4,4a,5,8,8a-hexahydronaphthalen-1-yl)methyl) malonate (23)** (400 MHz, $CDCl_3$)

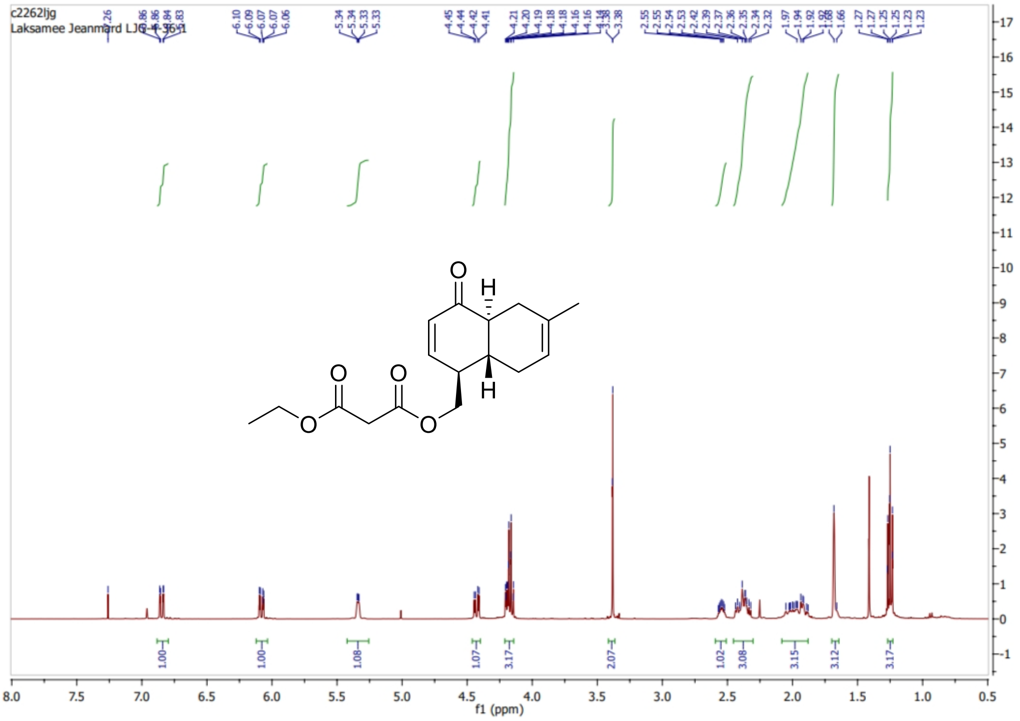

^{13}C NMR spectrum of **Ethyl (((1*S*,4a*S*,8a*R*)-6-methyl-4-oxo-1,4,4a,5,8,8a-hexahydronaphthalen-1-yl)methyl) malonate (23)** (100 MHz, $CDCl_3$)

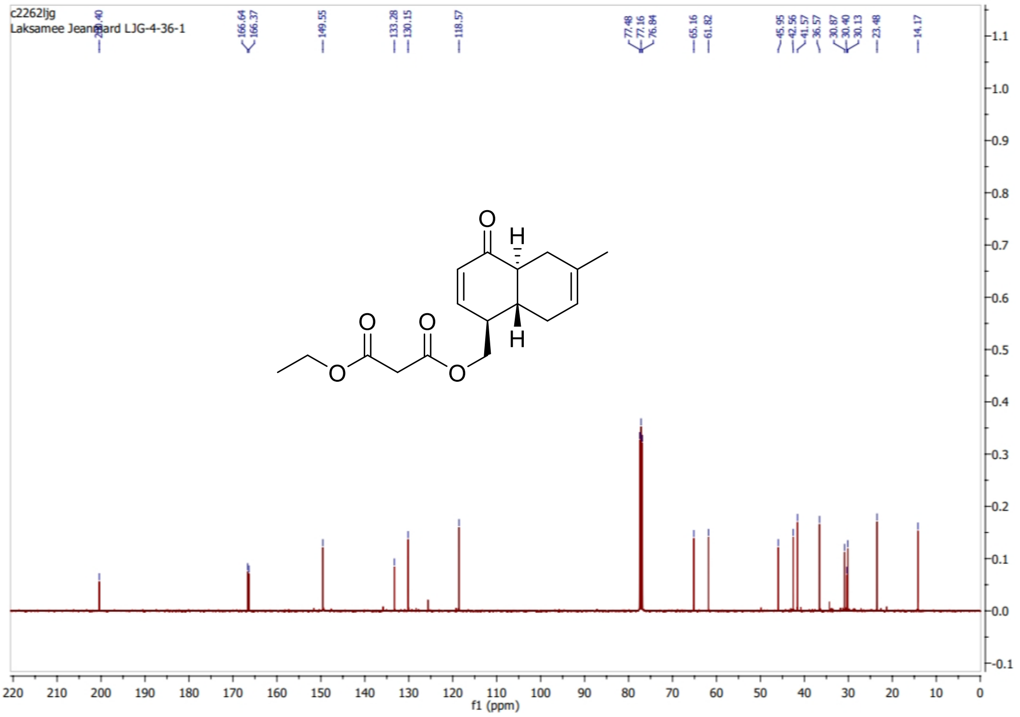

^{1}H NMR spectrum of **Ethyl (4*S*,4a*S*,6a*S*,10a*R*,10b*R*)-8-methyl-3,6-dioxo-3,4,4a,5,6,6a,7,10,10a,10b-decahydro-1*H*-benzo[*h*]isochromene-4-carboxylate (24)** (400 MHz, $CDCl_3$)

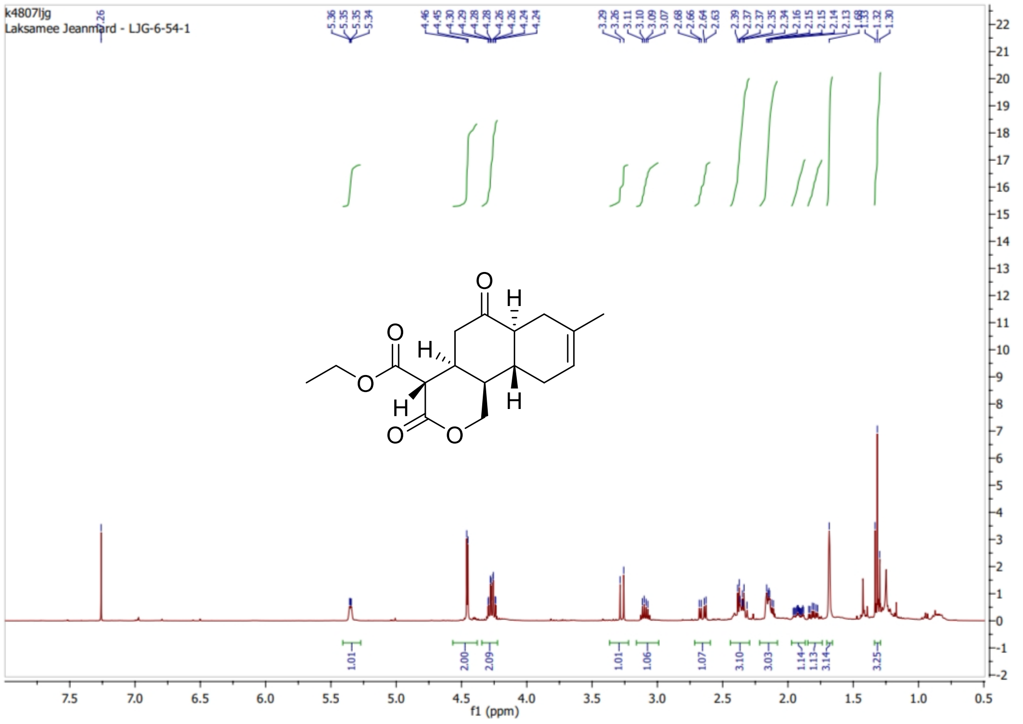

^{13}C NMR spectrum of **Ethyl (4*S*,4a*S*,6a*S*,10a*R*,10b*R*)-8-methyl-3,6-dioxo-3,4,4a,5,6,6a,7,10,10a,10b-decahydro-1*H*-benzo[*h*]isochromene-4-carboxylate (24)** (100 MHz, $CDCl_3$)

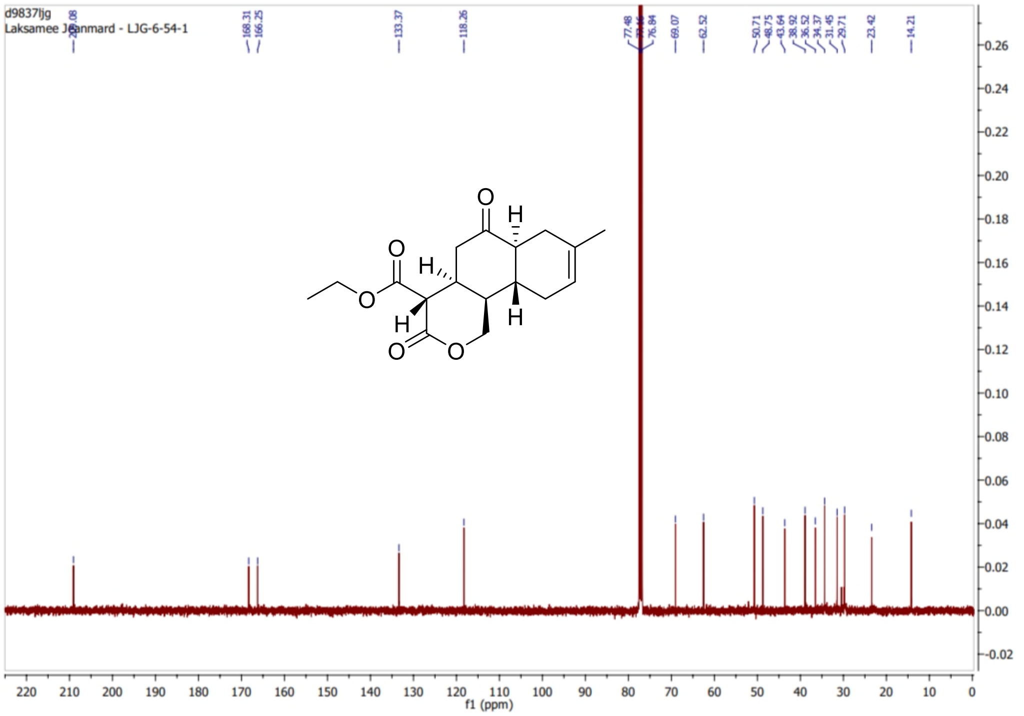

^{1}H NMR spectrum of **Ethyl (4*R*,4a*S*,6a*S*,10a*R*,10b*R*)-4,8-dimethyl-3,6-dioxo-3,4,4a,5,6,6a,7,10,10a,10b-decahydro-1*H*-benzo[*h*]isochromene-4-carboxylate (25)** (400 MHz, $CDCl_3$)

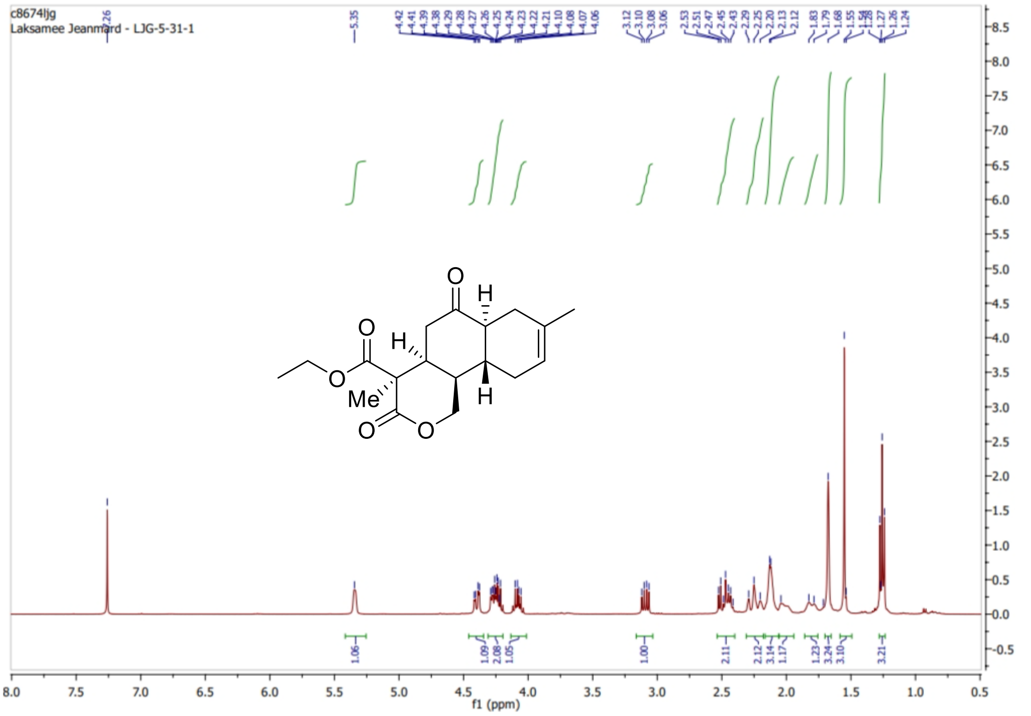

^{13}C NMR spectrum of **Ethyl (4*R*,4a*S*,6a*S*,10a*R*,10b*R*)-4,8-dimethyl-3,6-dioxo-3,4,4a,5,6,6a,7,10,10a,10b-decahydro-1*H*-benzo[*h*]isochromene-4-carboxylate (25)** (100 MHz, $CDCl_3$)

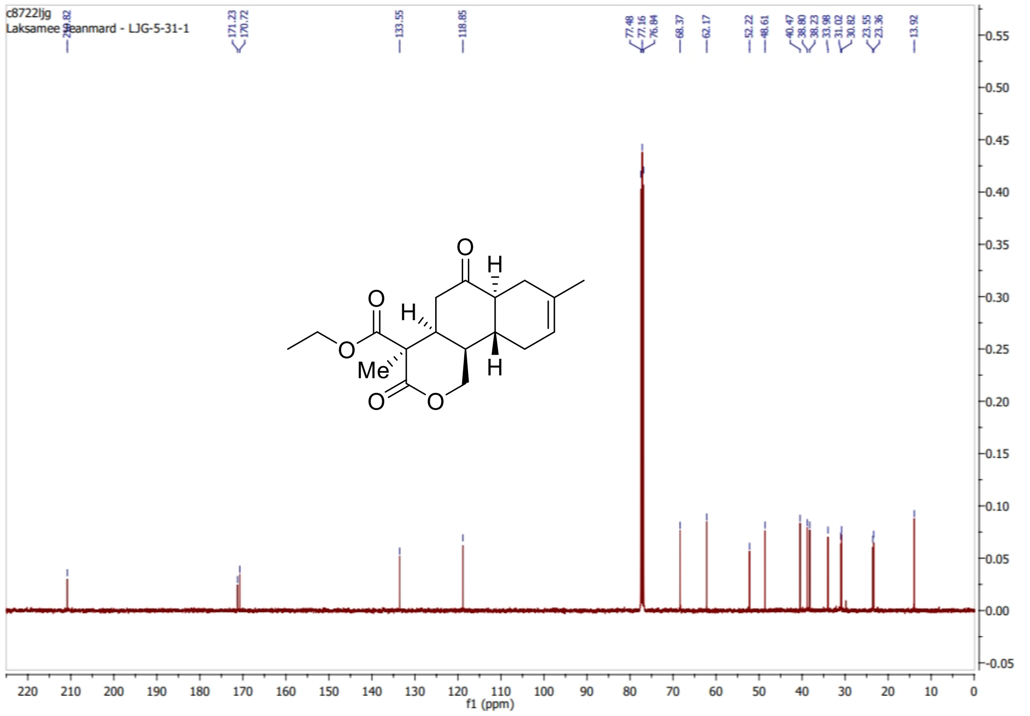

^{1}H NMR spectrum of **Ethyl (4*R*,4a*R*,6a*S*,10a*R*,10b*R*)-4,8-dimethyl-3-oxo-6-(((trifluoro methyl)sulfonyl)oxy)-3,4,4a,6a,7,10,10a,10b-octahydro-1*H*-benzo[*h*]isochromene-4-carboxylate (26)** (400 MHz, $CDCl_3$)

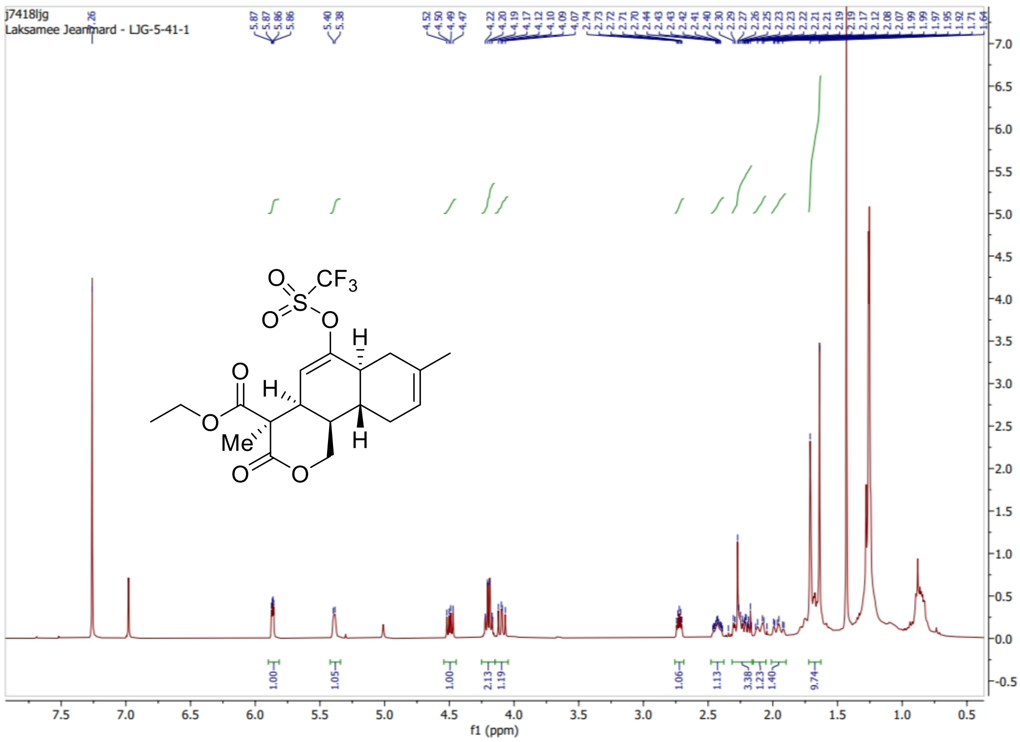

^{13}C NMR spectrum of **Ethyl (4*R*,4a*R*,6a*S*,10a*R*,10b*R*)-4,8-dimethyl-3-oxo-6-(((trifluoro methyl)sulfonyl)oxy)-3,4,4a,6a,7,10,10a,10b-octahydro-1*H*-benzo[*h*]isochromene-4-carboxylate (26)** (100 MHz, $CDCl_3$)

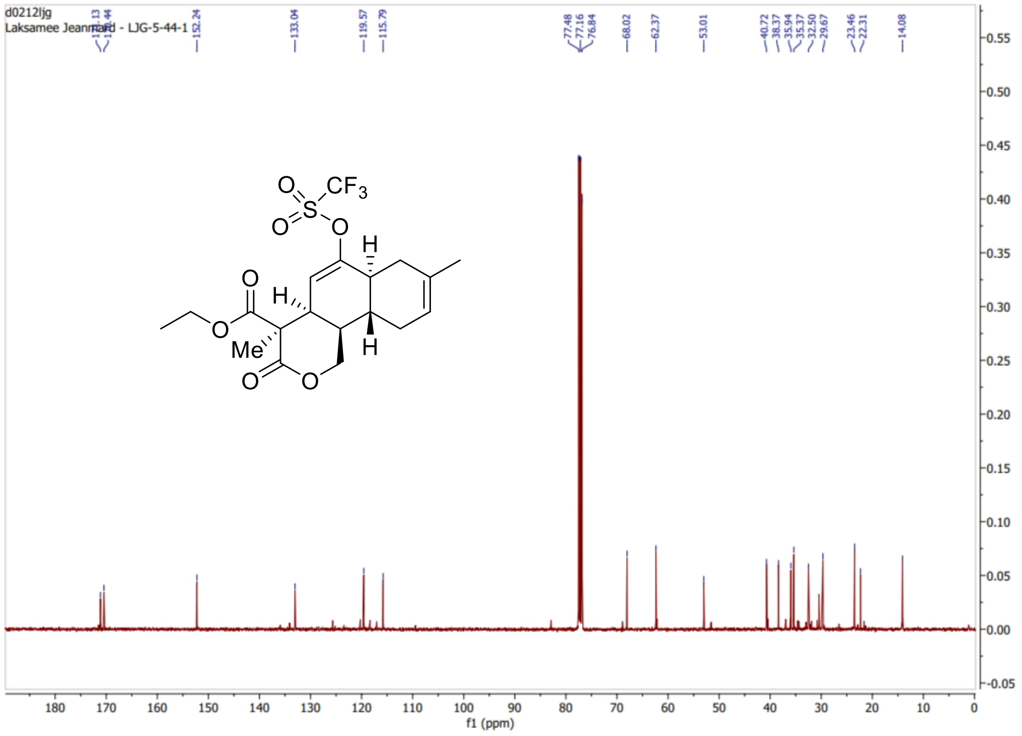

^{1}H NMR spectrum of **Ethyl (4*R*,4a*S*,6a*R*,10a*S*,10b*R*)-4,8-dimethyl-3-oxo-3,4,4a,6a,7,10,10a,10b-octahydro-1*H*-benzo[*h*]isochromene-4-carboxylate (27)** (400 MHz, $CDCl_3$)

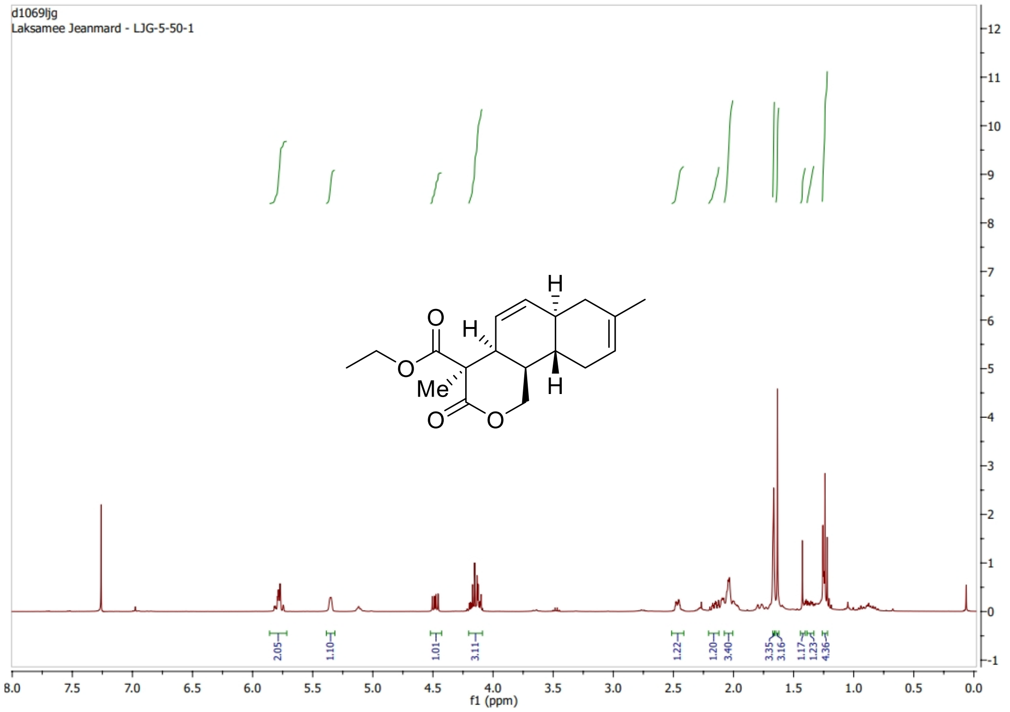

^{13}C NMR spectrum of **Ethyl (4*R*,4a*S*,6a*R*,10a*S*,10b*R*)-4,8-dimethyl-3-oxo-3,4,4a,6a,7,10,10a,10b-octahydro-1*H*-benzo[*h*]isochromene-4-carboxylate (27)** (100 MHz, $CDCl_3$)

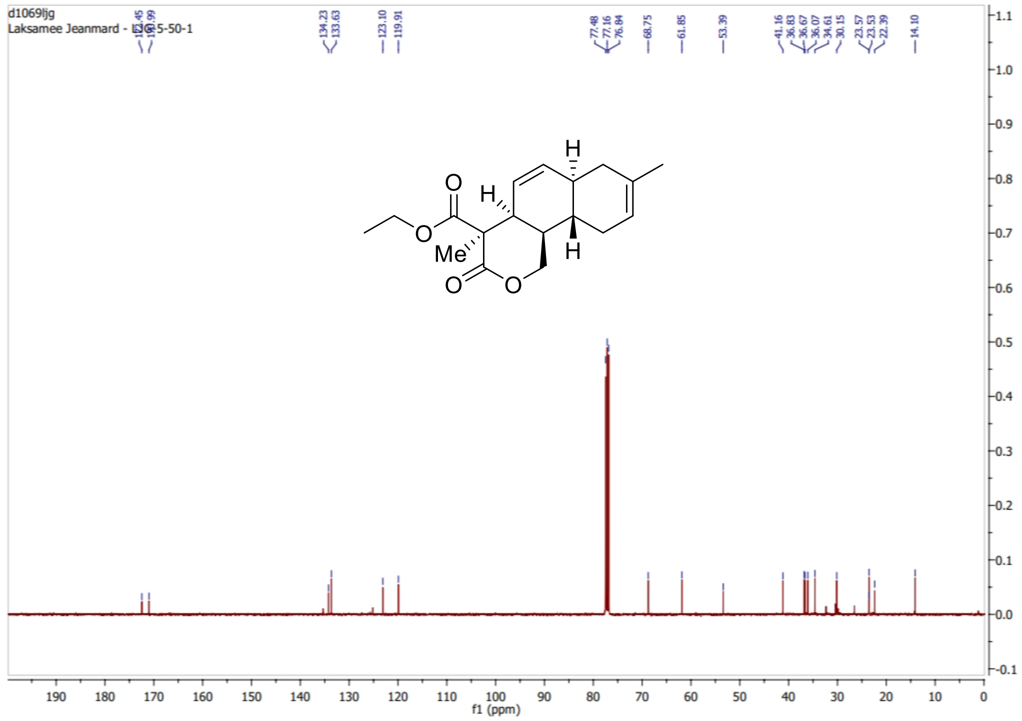

^{1}H NMR spectrum of **(4*R*,4a*S*,6a*R*,10a*S*,10b*R*)-4,8-Dimethyl-1,4,4a,6a,7,10,10a,10b-octahydro-3*H*-benzo[*h*]isochromen-3-one (2)** (400 MHz, $CDCl_3$)

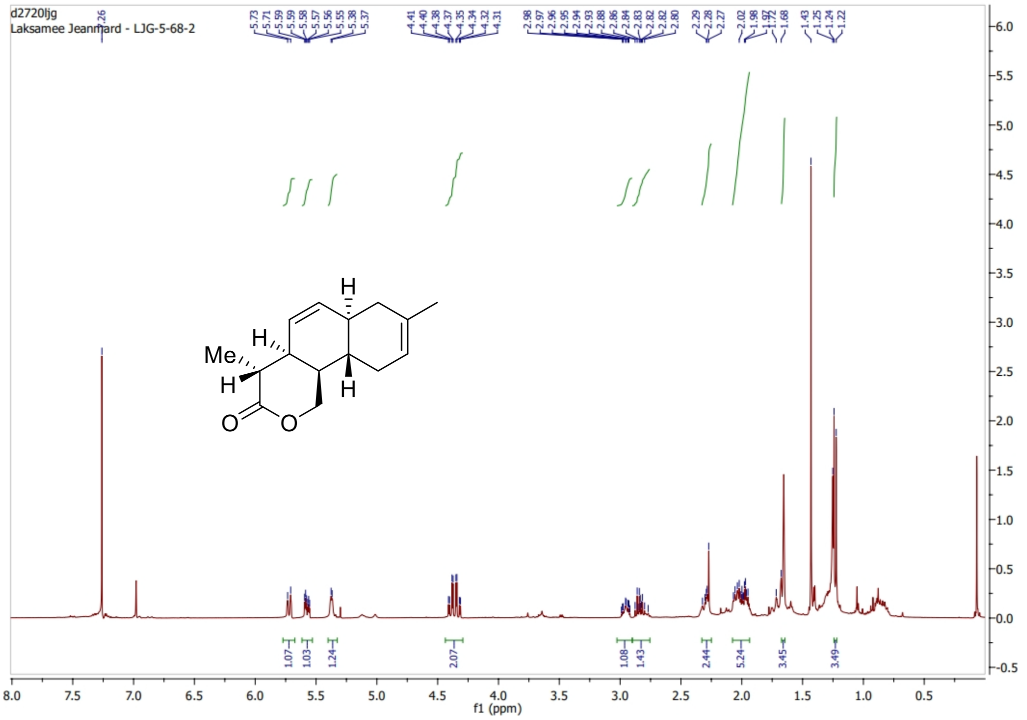

^{13}C NMR spectrum of **(4*R*,4a*S*,6a*R*,10a*S*,10b*R*)-4,8-Dimethyl-1,4,4a,6a,7,10,10a,10b-octahydro-3*H*-benzo[*h*]isochromen-3-one (2)** (100 MHz, $CDCl_3$)

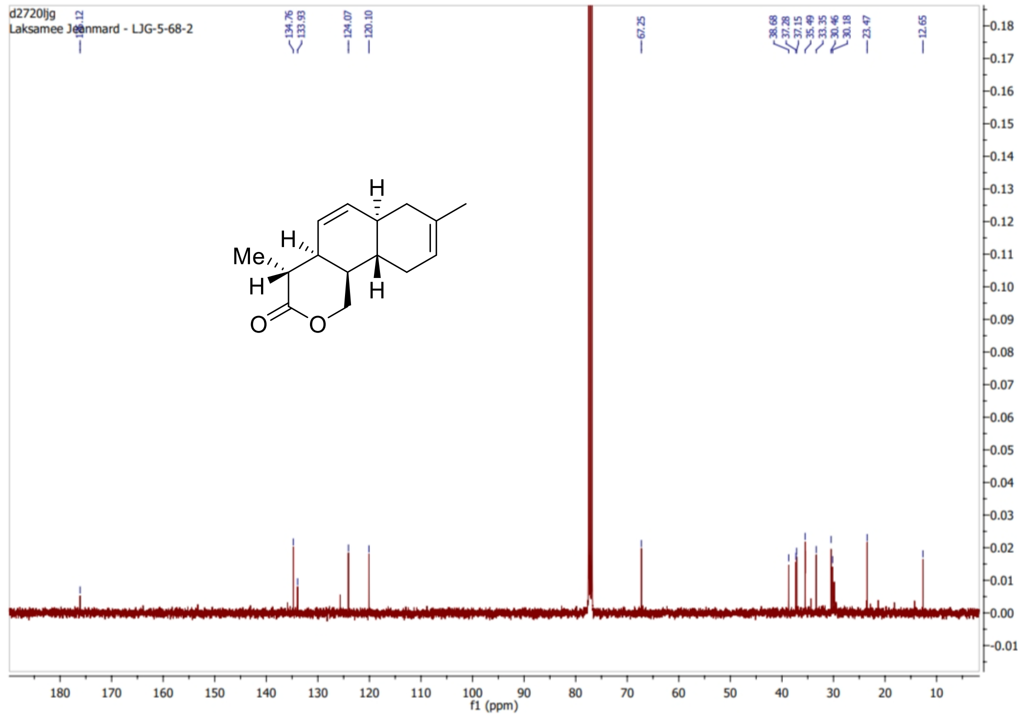

^{1}H NMR spectrum of **(4*S*,4a*S*,6a*R*,10a*S*,10b*R*)-4,8-Dimethyl-1,4,4a,6a,7,10,10a,10b-octahydro-3*H*-benzo[*h*]isochromen-3-one (28)** (400 MHz, $CDCl_3$)

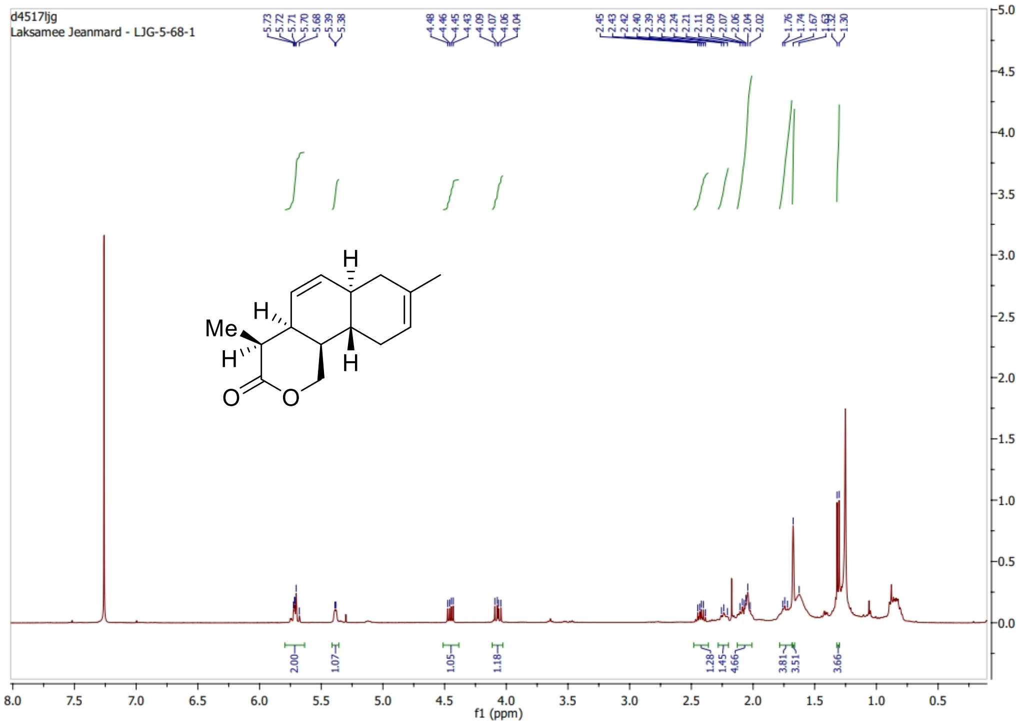

^{13}C NMR spectrum of **(4*S*,4a*S*,6a*R*,10a*S*,10b*R*)-4,8-Dimethyl-1,4,4a,6a,7,10,10a,10b-octahydro-3*H*-benzo[*h*]isochromen-3-one (28)** (100 MHz, $CDCl_3$)

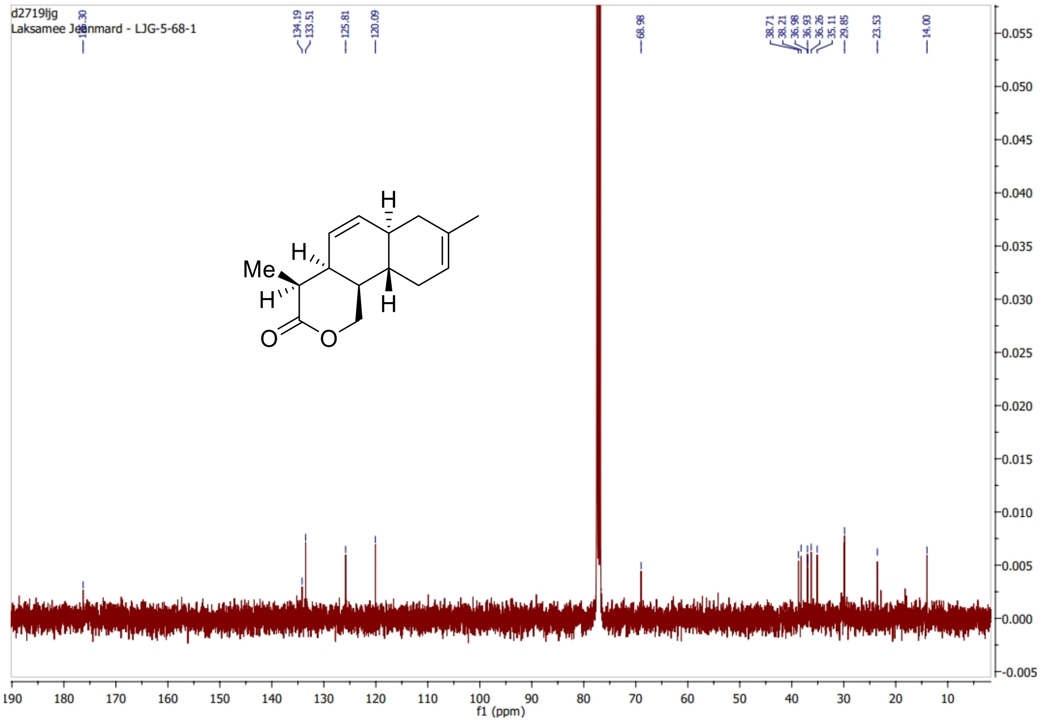

5) References

1) Y. Cui, H. Jiang, Z. Li, N. Wu, Z. Yang and J. Quan, *Org. Lett.*, 2009, **11**, 4628–4631.

2) J. M. Kelly and F. J. Leeper, *Tetrahedron Letters*, 2012, **53**, 819–821.

3) J.-E. Baeckvall and A. Ericsson, *J. Org. Chem.*, 1994, **59**, 5850–5851
